# Revision of the Neotropical diving beetle genus *Hydrodessus* J. Balfour-Browne, 1953 (Coleoptera, Dytiscidae, Hydroporinae, Bidessini)

**DOI:** 10.3897/zookeys.580.8153

**Published:** 2016-04-12

**Authors:** Kelly B. Miller

**Affiliations:** 1 Department of Biology and Museum of Southwestern Biology, University of New Mexico, MSC03 2020, Albuquerque, NM 87131-0001, USA

**Keywords:** Water beetles, taxonomy, classification, Neotropical, Hydrodessus, Dytiscidae, Coleoptera

## Abstract

The Neotropical diving beetle genus *Hydrodessus* J. Balfour-Browne, 1953 (Coleoptera: Dytiscidae: Hydroporinae: Bidessini) is revised. Thirty species are recognized. The following new species are described: *Hydrodessus bimaculatus* **sp. n.** (Venezuela), *Hydrodessus brevis* **sp. n.** (Venezuela), *Hydrodessus concolorans* **sp. n.** (Venezuela), *Hydrodessus continuus* **sp. n.** (Venezuela), *Hydrodessus disjunctus* **sp. n.** (Suriname), *Hydrodessus fasciatus* **sp. n.** (Brazil), *Hydrodessus imparilis* **sp. n.** (Ecuador), *Hydrodessus keithi* **sp. n.** (Brazil, Colombia, Ecuador), *Hydrodessus kurti* **sp. n.** (Suriname), *Hydrodessus kylei* **sp. n.** (Suriname, Venezuela), *Hydrodessus laetus* **sp. n.** (Venezuela), *Hydrodessus latotibialis* **sp. n.** (Peru), *Hydrodessus maculatus* **sp. n.** (Guyana, Venezuela), *Hydrodessus morsus* **sp. n.** (Venezuela), *Hydrodessus palus* **sp. n.** (Venezuela), and *Hydrodessus tenuatus* **sp. n.** (Suriname). The following new synonyms are established: *Hydrodessus fragrans* Spangler, 1985 = *Hydrodessus biguttatus* (Guignot, 1957) **syn. n.** and *Hydrodessus robinae* Spangler, 1985 = *Hydrodessus octospilus* (Guignot, 1957), **syn. n.** One species is transferred from *Hydrodessus* to *Amarodytes* Régimbart, *Amarodytes soekhnandanae* (Makhan, 1994), **comb. n.** Habitus photographs (dorsal and lateral) and photos of the ventral surfaces are provided for most species. Line drawings of male and female genitalia and other diagnostic features are also provided along with distribution maps.

## Abstract (es)

El género neotropical de escarabajos aquáticos *Hydrodessus* J. Balfour-Browne, 1953 (Coleoptera: Dytiscidae: Hydroporinae: Bidessini) es revisado. Se reconocen treinta especies. Se describen las siguientes nuevas especies: *Hydrodessus bimaculatus* **sp. n.** (Venezuela), *Hydrodessus brevis* **sp. n.** (Venezuela), *Hydrodessus concolorans* **sp. n.** (Venezuela), *Hydrodessus continuus* **sp. n.** (Venezuela), *Hydrodessus disjunctus* **sp. n.** (Suriname), *Hydrodessus fasciatus* **sp. n.** (Brazil), *Hydrodessus imparilis* **sp. n.** (Ecuador), *Hydrodessus keithi* **sp. n.** (Brazil, Colombia, Ecuador), *Hydrodessus kurti* **sp. n.** (Suriname), *Hydrodessus kylei* **sp. n.** (Suriname, Venezuela), *Hydrodessus laetus* **sp. n.** (Venezuela), *Hydrodessus latotibialis* **sp. n.** (Peru), *Hydrodessus maculatus* **sp. n.** (Guyana, Venezuela), *Hydrodessus morsus* **sp. n.** (Venezuela), *Hydrodessus palus* **sp. n.** (Venezuela), y *Hydrodessus tenuatus* **sp. n.** (Suriname). Se establecen los siguientes nuevos sinónimos: *Hydrodessus fragrans* Spangler, 1985 = *Hydrodessus biguttatus* (Guignot, 1957), **syn. n.** y *Hydrodessus robinae* Spangler, 1985 = *Hydrodessus octospilus* (Guignot, 1957), **syn. n.** Una especie se transfiere de *Hydrodessus* a *Amarodytes* Regimbart, *Amarodytes soekhnandanae* (Makhan, 1994), **comb. n.** Se proporcionan fotografías del hábito (dorsal y lateral) y de las superficies ventrales para la mayoría de las especies. También se presentan dibujos de los genitales masculinos y femeninos y otras características diagnósticas, junto con mapas de distribución.

## Introduction

*Hydrodessus* Balfour-Browne, 1953, was described to include a new species that Balfour-Browne (1953) thought might be an “…abnormal member of Bidessini…,” but he was not certain. Young (1969) placed *Hydrodessus* in Bidessini, but it was later removed from that tribe (along with *Amarodytes* Régimbart) by Biström (1988) who placed it as *incerta sedis* with respect to tribe since members of the group lack bisegmented lateral lobes, the presence of which was then regarded as the only reliable synapomorphy of that tribe (Biström 1988). *Amarodytes* was later returned to Bidessini (Miller 2001) since at least some of its members have a spermathecal spine, bisegmented lateral lobes (in at least some species, see Benetti and Régil Cueto 2004), and crusher lobes of the proventriculus with five prominences, each of which characterizes members of Bidessini according to Miller et al. (2006). *Hydrodessus* was still, however, *incerta sedis* with respect to tribe (Nilsson 2001). A recent phylogenetic analysis by Miller and Bergsten (2014) resulted in *Hydrodessus* related to *Peschetius* and some *Amarodytes*, and this clade sister to the rest of Bidessini. The clade *Peschetius* + some *Amarodytes* + *Hydrodessus* does not have a known morphological synapomorphy, but this clade + other Bidessini (Bidessini in the broadest sense) has the distinctive synapomorphies of a spermathecal spine (absent or reduced in some *Hydrodessus*) and the crusher lobes of the proventriculus with five prominences (though not surveyed in all taxa, including most Hydrodessus, Miller et al. 2006). Based on this, *Hydrodessus* is recognized here as a genus of Bidessini following Miller et al. (2006). Given this history, it should be clear that much work remains needed to clarify relationships among these taxa. An important first step is to make better known the species in the group, which is the goal of this paper.

In general, members of this group are rarely collected with most specimens in collections found using lights at night. Only a few species have been collected in long series, though some of these series do include many species A few specimens have been collected from forest streams or stream margins, but little to nothing else is known of the biology of most *Hydrodessus* species.

New species have been described regularly over several years (Balfour-Browne 1953; Guignot 1957; Spangler 1966; 1985; Young 1970; Makahn 1994). Fortunately, these descriptions have largely been in the context of the group as a whole with keys and comparative diagnoses such that new species have largely been confidently identified as such. Discovery of a large number of new species, especially as the result of recent collecting in northern South America, and re-examination of the known species in light of the new discoveries have made clear, however, the need for a broad review of the genus. The goal of this project is to describe, key and illustrate all species in the genus, including 16 new ones.

## Materials and methods

**Dissections.** Examination of male genitalia is critical for many *Hydrodessus* species determinations. Males were dissected by first relaxing the specimen in near boiling water. The genital capsule was then removed by inserting a pin with the apex bent into the side of the apex of the abdomen and hooking the base of the median lobe and pulling it out. The genitalia were then further disarticulated in a drop of glycerin on a microscope slide to isolate the median lobe and lateral lobes from other structures. All structures were then placed into a genitalia vial in glycerin and mounted on the pin with the specimen. Male genitalia were examined in glycerin.

Female genitalia were examined by first relaxing a specimen in near boiling water. A pin was then inserted into the end of the abdomen and moved along the suture between abdominal ventrites VI and VII and between tergites VII and VIII. The lateral junction of these sclerites was then cut with microscissors. Fine microforceps were then inserted into the abdomen and the female internal genital structures were grasped and the entire internal abdominal apex removed. These structures were then placed into a small glass tube with a 10% KOH solution. This tube was then placed in near boiling water to heat the KOH for about 10 minutes to macerate the soft tissues. The remaining structures were removed and placed in a weak acetic acid solution and then rinsed in much distilled water. Structures were stained using an aqueous solution of Chlorazol Black®. Structures were then placed into a genitalia vial in glycerin and mounted on the pin with the specimen. Female genitalia were examined in water. Examination in glycerin is not preferable since structures collapse, but in water they expand and are easily visible.

Female genitalia are not described for all species here either because females are not available, the genitalia are damaged due to previous attempts to dissect the specimen, or female specimens are determined to be too rare or valuable to risk a dissection attempt which is often somewhat destructive to the specimen.

**Measurements.** Measurements were taken with an ocular scale on a Zeiss Discovery V8 dissecting microscope. Emphasis was placed on getting the diagnostic minimum and maximum measurements of structures rather than finding the average or taking a random sample. Measurements include: 1) total length (TL), 2) greatest width across elytra (GW), 3) greatest width of pronotum (PW), 4) greatest width of head (HW), and 5) distance between eyes (EW). The ratios TL/GW and HW/EW are also provided.

**Descriptions.** Descriptions are based on examined specimens, except in the cases of *Hydrodessus amazonensis* Spangler and *Hydrodessus nanayensis* Spangler each of which is known only from type specimens which were not located. In these cases, the published descriptions were modified to conform to the descriptions included here for the other species.

**Drawings.** Illustrations were made using a drawing tube on a Zeiss Discovery V8 dissecting microscope. Sketches were first done in pencil then scanned, placed into Adobe Illustrator and “inked” digitally using vector-based graphics.

**Material.** *Hydrodessus* specimens are not common in collections, and only a few have larger numbers of specimens or series. Primary type specimens were examined for all species except *Hydrodessus amazonensis* Spangler, *Hydrodessus nanayensis* Spangler, *Hydrodessus angularis* Young, *Hydrodessus surinamensis* Young, *Hydrodessus biguttatus* Guignot, and *Hydrodessus siolii* J. Balfour-Browne. Paratypes were examined for some of these, and, in some cases, comparisons of specimens with descriptions were adequate to delimit species limits. Specimens were borrowed from several collections including the following:

FSCA
 Florida State Collection of Arthropods, University of Florida, USA (P. Skelley) 


KBMC
 Kelly B. Miller Collection, Museum of Southwestern Biology, University of New Mexico, USA 


MIZA
 Museo del Instituto de Zoología Agrícola Francisco Fernández Yépez, Universidad Central de Venezuela, Maracay, Venezuela (L. Joly) 


MSBA
Museum of Southwestern Biology Division of Arthropods, University of New Mexico, Albuquerque, NM, USA (K.B. Miller) 


MZSP
 Museu de Zoologia da Universidade de São Paulo, São Paulo, Brasil (S. Casari) 


NZCS
 National Zoological Collection of Suriname, Paramaribo, Suriname (P. Ouboter) 


RMNH
Naturalis Biodiversity Center and Leiden University, The Netherlands (H. Huijbregts) 


SEMC
 Snow Entomological Collection, University of Kansas, Lawrence, Kansas, USA (A.E.Z. Short) 


USNM
 United States National Collection of Insects, Smithsonian Institution, Washington, DC, USA (T. Erwin) 


Label data for primary type specimens is reported verbatim. All other label data, including for paratypes, is reported in a standardized format. All paratypes of new species have attached a blue label with a black line border bearing the species name.

### Taxonomic characters

*Coloration*. Most *Hydrodessus* have even coloration on the head and pronotum and maculae on the elytra, though the head and pronotum are often (not always) a different color. Some species are immaculate, or nearly so, on the elytra. The color pattern may be well-delimited or only vaguely present. The basic pattern on the elytron in most species is a large pale macula near the anterior margin that extends from the lateral margin to near the suture, another pale, subtriangular macula subapically, and the apex of the elytra pale, but there is much variation with some species without certain maculae and others with pale regions enlarged or different in shape. The ventral surface is usually approximately concolorous on most ventrites with the legs, elytral epipleuron and apex of the abdomen lighter in color.

*Body shape*. *Hydrodessus* have considerable variability in body shape from elongate and relatively slender to short and robust. Most specimens have the lateral body outline distinctly and strongly discontinuous between the pronotum and elytron. A few have this discontinuity less pronounced.

*Surface sculpturing*. Most specimens of *Hydrodessus* have most surfaces relatively densely punctate. A few are shiny with more sparse punctation, and a few have some microreticulation, particularly on the dorsal surface of the head and the pronotum.

*Head*. Head shape is variable from broad to rounded to slightly elongate. The anterior clypeal margin is typically not strongly modified. Usually it is broadly rounded but varies from subtruncate to somewhat produced. Some species, including *Hydrodessus angulatus* have the anterior clypeal margin anteriorly produced and somewhat beaded. The eyes are a little variable in size, but not greatly.

*Pronotum*. The pronotum of many species is cordate with the greatest width near the anterior margin, a distinct constriction posterad of the middle, and the posterolateral angles acute. A few species have the pronotum somewhat less cordate with the greatest width only slightly anterad of the middle, and a few species have the lateral pronotal margins more evenly curved.

*Elytra*. The elytron varies in relative length and width and degree of curvature of the lateral margins. Together, the elytral apices range from moderately rounded to pointed with osp. n.cies (*Hydrodessus biguttatus*) having the elytral apices slightly but distinctly dehiscent. The elytron laterally is variable with most species having the elytral/epipleural carina distinctly descending at the humeral angle, though in a couple species the carina extends directly posterad from the humeral angle. In many species with a descending carina, a secondary carina is developed at the humeral angle and extends posteriorly along the lateral surface of the elytron. This secondary carina, if present at all, varies from short, rounded, and limited to the area adjacent to the humeral angle to well-developed, sharply carinate and extending for much of the length of the elytron.

*Prosternum*. The prosternum medially ranges from nearly flat to distinctly carinate. The prosternal process is an important, variable character between species of *Hydrodessus*. It ranges from moderately broad to extremely broad. The apex may be narrowly rounded to broadly truncate. In most species the blade of the process is longitudinally distinctly impressed. The lateral margins may be curved, subparallel or medially constricted to posteriorly convergent.

*Metasternum*. The anteromedial portion of the metaventrite in *Hydrodessus* extends anteriorly between the mesocoxa as the metasternal process. This process extends anteriorly to meet the prosternal process, and the apex may be broad and meet the prosternal process broadly, or more narrowly rounded and only interfacing narrowly with the prosternal process. The surface of the process may be flat to distinctly longitudinally impressed. The lateral margins of the process are distinctive in all species, and in many species these margins extend posteriorly on the surface of the metaventrite as a pair of carinae. These carinae may be well developed and extend posteriorly to the posterior margin of the metaventrite at or near the anterior limit of the metacoxal lines. In some species the metaventrite carinae and the metacoxal lines form a continuous carina. In others, the metaventrite carina meets the posterior margin mediad of the metacoxal lines. The carinae may be straight or variously curved, they may be only slightly divergent posteriorly, or strongly so. In many taxa they do not extend across the entire metasternum, and in some they extend across the metaventrite only as lines of impunctate surface between the otherwise punctate regions of the sclerite.

*Legs*. The legs of *Hydrodessus* are relatively long. Variable features include the relative width of the pro- and mesotibiae and the shape of the metatrochanter and degree to which it is offset from the metafemur. The metacoxae vary in the degree of punctation on the surface and the relative width of the medial portion (distance between the metacoxal lines). The posteroapical surface of the metafemur is characterized by a series of spinous setae that are apically somewhat hooked, and increase in length apically.

*Abdomen*. The surface of the abdomen is somewhat variable in degree of punctation, and the apex of abdominal ventrite VI is somewhat variable in degree of curvature of the margin. It varies from rounded to relatively pointed.

*Female genitalia*. The internal female reproductive tract has an overall configuration typical of Hydroporinae (i.e. two genital openings with separate spermathecal and fertilization tracts). The length of the spermathecal and fertilization ducts varies between species and are quite long in many species. Species generally do not have a distinctive differentiation between the receptacle asp. n.rmatheca. Some species have a distinctive spermatheca spine, but others have a reduced spermatheca and do not have a spine.

*Male genitalia*. The male median and lateral lobes of the aedeagus are the most dispositive diagnostic structures in *Hydrodessus*. The lateral lobes are single-segmented and variable in shape, but are bilaterally symmetrical. The median lobe is variable in shape in both dorsal and ventral aspect. Most species have the median lobe bilaterally symmetrical, but a few species are distinctly asymmetrical.

*Other sexually dimorphic features*. Males have the pro- and mesotarsi somewhat more broadly expanded laterally with the ventral surface bearing several large adhesive setae. Females have the ventral surfaces with long, filamentous setae only. Some females of some species are more alutaceous on dorsal and ventral surfaces. Females of some species have the elytra distinctly expanded and lobate subapically with a corresponding impressed area on each side of abdominal ventrite VI or shorter and apically more rounded. Males of these species have the elytra evenly curved to a pointed apex and ventrite VI unmodified.

## Taxonomy

### Hydrodessus

Taxon classificationAnimaliaColeopteraDytiscidae

J. Balfour-Browne, 1953

Figs 1–7 , 8–10 , 11–13 , 14–16 , 17–19 , 20–22 , 23–25 , 26–28 , 29–31 , 32–34 , 35–41 , 42–47 , 48–51

Hydrodessus J. Balfour-Browne, 1953: 55 (type species: Hydrodessus siolii J.Balfour-Browne, 1953: 56 by original designation); Young 1967: 80, 83; 1969: 2; Biström 1988: 36; Nilsson 2001: 236, 2013: 214.
Brinckius Guignot, 1957: 38 (type species: Brinckius biguttatus Guignot, 1957: 39 by original designation); Biström 1988: 37; Nilsson 2001: 236, 2013: 214; synonymy by Young 1969: 2.
Brinkius , Young 1967: 80, 83; 1969: 2 (incorrect subsequent spelling).

#### Diagnosis.

*Hydrodessus* are distinguishable from other Bidessini by the following combination: 1) the lateral lobes of the aedeagus comprised of a single segment (instead of two or three), 2) without basal pronotal striae, and 3) without prominent carinae on the disc of elytron and no large pores on dorsal and ventral surfaces. In addition, *Hydrodessus* do not have basal elytral striae, modifications to the anterior clypeal margin (except in one species), a transverse occipital line between the posterior margins of the eyes, nor a transverse carinae across the elytral epipleuron at the humeral angle.

#### Natural history.

Relatively little is know of the natural history of most members of the group. A great many museum specimens were collected at lights. Other specimens were collected from forest streams, often in low numbers. Occasionally, longer series have been found in tropical forest streams. Larvae and other aspects of their natural history have not been described.

#### Taxonomic history.

*Hydrodessus* has a complicated character combination, and because of this has had a history of ambiguous taxonomic placement. The genus was early placed in or near Bidessini, but not without reservation (Guignot 1957). Though Young (1967; 1969) classified it in Bidessini, Biström (1988) restricted the definition of that tribe to those Hydroporinae with bi- or trisegmented lateral lobes, which are single-segmented in *Hydrodessus* (and at least some *Amarodytes* (Benetti and Régil Cueto 2004)). *Hydrodessus* was subsequently placed *incerta sedis* with respect to tribe until Miller and Bergsten (2014) placed it back into Bidessini. This was based on a large phylogenetic analysis including many DNA sequence data and morphology which resulted in *Hydrodessus* together with *Peschetius* (previously placed in Bidessini by Miller et al. (2006)) and some *Amarodytes* in a clade, and this sister to other Bidessini. Miller et al. (2006) expanded the definition of Bidessini to include taxa with 1) a spermathecal spine, and 2) five lobes on the crusher teeth of the proventriculus, which resulted in *Peschetius* and *Amarodytes* included in the tribe, but *Hydrodessus* was not examined comprehensively at that time. Based on evidence gathered for this revision, at least some *Hydrodessus* have a spermathecal spine, though not all do, and some have five-lobed crusher teeth on the proventriculus, though not all were examined. Based on this, and on evidence from Miller and Bergsten (2014), the genus is recognized here in Bidessini, and related to *Peschetius* and (at least) some *Amarodytes*.

The first species of *Hydrodessus*, *Hydrodessus siolii* J. Balfour-Browne, was described along with the genus description (Balfour-Browne 1953). Subsequent to this, Guignot (1957) erected the new genus, *Brinckius* Guignot, with four new species. Spangler (1966) added two new species from Peru to *Hydrodessus*. In his treatment of the genera of New World Bidessini, Young (1967) was uncertain whether to synonymize *Brinckius* with *Hydrodessus*, though he keyed them out together. Nilsson (2001) regarded the synonymy of *Brinckius* with *Hydrodessus* to date to Young’s (1967) paper. However, Young (1967, 83) seemed to make it clear at that time that he could not “…decide… whether *Brinkius* [sic] of Guignot should be accorded recognition.” Even so, he soon (Young 1969) did synonymize *Brinckius* with *Hydrodessus* and provided a list of the included species. He then (Young 1970) added two more and provided a key to all the species. The next contribution was by Spangler (1985), who added five new species from Guyana and also provided a key to the species. Though not included in his concept of Bidessini, Biström (1988) listed the species. The last addition of species to the genus was three by Makhan (1994), bringing the total to 17 valid *Hydrodessus* species prior to this revision.

Monophyly of *Hydrodessus* as deliminated here has not been demonstrated, and all the known diagnostic features described here for the genus are plesiomorphies. Other distinctive characters (potential synapomorphies) are variable within the genus. Many species have a lateral carina on the elytron extending posteriorly from the humeral angle, but not all do, and some of those that do have it only weakly developed. Most also have longitudinal carinae on the metaventrite approximately continuous with the metacoxal lines, but not all do. These two characters are also not always in the same combinations. All species have a similar overall appearance, robust, laterally discontinuous between the pronotum and elytron, elongate, with a variety of color patterns, and a somewhat characteristic shape for the prosternal and metasternal processes, but these are not particularly convincing as synapomorphies. Future research should concentrate on carefully examining the monophyly of the group and its relationships with *Amarodytes* and *Peschetius*, and possibly some *Hypodessus* Guignot, as well. It seems likely that *Hydrodessus* may eventually need division into multiple genera.

#### Distribution.

*Hydrodessus* are characteristic mainly of northern South America from Ecuador and Peru to Brazil. The greatest known density of species is from southern Venezuela to Suriname. There are a few species extending south to Paraguay.

#### Key to the species of *Hydrodessus*

Two species, *Hydrodessus amazonensis* and *Hydrodessus nanayensis*, are problematic since no specimens were examined (the types were not found). *Hydrodessus nanayensis* is included in the key since, based on previous work, it appears to be very similar to (if not identical with) *Hydrodessus siolii*. The other species, *Hydrodessus amazonensis*, is not easily keyed with the characters included here since many of the states important for the key are not described for that species. It is included in the species treatments, however, and the male genitalia are relatively distinctive and diagnostic.

**Table:** 

1	Size very small (TL < 1.7 mm)	***Hydrodessus morsus* sp. n.**
–	Size larger (TL > 2.0 mm)	**2**
2(1)	Lateral elytral carina short (<1/4 elytral length) (Fig. 27B) or absent (Fig. 31B)	**3**
–	Lateral elytral carina long (≥1/4 elytral length) (Fig. 35B)	**17**
3(2)	Basal half of elytron approximately concolorous on disc, without distinct maculae (Fig. 27A), at most with diffuse, poorly-defined fasciae, though apical half of elytron often with maculae (as in Fig. 20A)	**4**
–	Basal half of elytron with distinctive maculae or fasciae (e.g. Fig. 22A)	**11**
4(3)	Dorsal and ventral surfaces nearly concolorous, without maculae, though color may vary somewhat in intensity across surfaces (as in Fig. 27A); carinae on metaventrite somewhat divergent posteriorly (as in Fig. 27C)	**5**
–	Dorsal and ventral surfaces red to red-brown, apical half of elytron with irregular, subtriangular maculae and apex of elytron pale orange to yellow, pronotum of many specimens lighter orange, lighter in color than elytron (as in Fig. 20A); carinae on metaventrite strongly divergent posteriorly (as in Fig. 20C)	**7**
5(4)	Size larger (TL > 3.0 mm)	***Hydrodessus pereirai* (Guignot)**
–	Size smaller (TL < 3.0 mm)	**6**
6(5)	Size smaller (TL = 2.0 mm); dorsal and ventral surfaces yellow (Fig. 27A)	***Hydrodessus palus* sp. n.**
–	Size larger (TL = 2.5 mm); dorsal and ventral surfaces red (Fig. 12A)	***Hydrodessus brevis* sp. n.**
7(4)	Eyes entire (Fig. 21A) or emarginate (Fig. 20A) in dorsal aspect; male and female body shape dimorphic, male apically evenly tapered, female shorter and apically broadly rounded (as in Fig. 20A)	**8**
–	Eyes entire; male and female similar in shape, both apically evenly tapered	**9**
8(7)	Eyes entire in dorsal aspect (Fig. 20A); male median lobe in lateral aspect broadly curved, basal portion elongate triangular, apex sinuate (Fig. 20D); median lobe in ventral aspect bilaterally symmetrical, apically narrowly rounded (Fig. 20E); lateral lobe moderately broad (Fig. 20F)	***Hydrodessus kurti* sp. n.**
–	Eyes emarginate in dorsal aspect (Fig. 21A); male median lobe in lateral aspect very broadly curved, basal portion very broadly triangular, apex somewhat sinuate (Fig. 21D); median lobe in ventral aspect bilaterally asymmetrical, apically broadly expanded and truncate (Fig. 21E); lateral lobe very broad (Fig. 21F)	***Hydrodessus kylei* sp. n.**
9(7)	Prosternal process with lateral margins subparallel, slightly concave, posteriorly broadly rounded (Fig. 32C); female with apicolateral margin of elytron developed into flange (as in Fig. 5B)	***Hydrodessus spanus* Spangler**
–	Prosternal process anteriorly with prominent, lateral lobes, posteriorly distinctly tapered to rounded apex (as in Fig. 28C); female without apicolateral flange along margin of elytron	**10**
10(9)	Male median lobe bilaterally symmetrical in ventral aspect, apex rounded (Fig. 28E)	***Hydrodessus peloteretes* Spangler**
–	Male median lobe bilaterally asymmetrical in ventral aspect, apex obliquely truncate (Fig. 17E)	***Hydrodessus imparilis* sp. n.**
11(3)	Prosternal process very broad (length/width < 1.8), lateral margins rounded, and broadly concave medially (as in Fig. 22C); carinae on metaventrite prominent, extending posteriorly to posterior margin (as in Fig. 22C)	**12**
–	Prosternal process narrower (length/wdith > 2), lateral margins variable, subparallel to sinuate, narrowly longitudinally concave medially (as in Fig. 33C); cariane on metaventrite indistinct, not generally extending to posterior margin except as narrow impunctate area (as in Fig. 33C)	**13**
12(11)	Prosternal process very broad, apically broadly subtruncate (Fig. 22C); lateral pronotal margins broadly curved, greatest width medially (Fig. 22A)	***Hydrodessus laetus* sp. n.**
–	Prosternal process broad but elongate with lateral margins broadly rounded and apex acuminate (Fig. 30C); pronotal margins not broadly curved, greatest width posterior to middle (Fig. 30A)	***Hydrodessus rattanae* Makhan**
13(11)	Prosternal process anteriorly with prominent, laterally-projecting lobes, lateral carinae distinctly convergent to narrowed apex (Fig. 33C); metaventrite carinae slightly divergent posteriorly, area between carinae narrow, apices of carinae terminating slightly mediad of anterior apices of metacoxal lines (Fig. 33C)	***Hydrodessus surinamensis* Young**
–	Prosternal process anteriorly without or with weak, laterally-projecting lobes, lateral carinae subparallel to rounded apex (as in Fig. 19C); metaventrite carinae strongly divergent posteriorly, area between carinae broad posteriorly, apices of carinae terminating near anterior apices of metacoxal lines (as in Fig. 19C)	**14**
14(13)	Elytron with two large pale regions, one quadrate macula laterally at anterolateral margin and one subtriangular, subapical maculae (Fig. 19A); males and females dimorphic, female elytron with distinct subapical lateral lobe (Fig. 5)	***Hydrodessus keithi* sp. n.**
–	Elytral maculae not consisting of two large pale regions, either consisting of complex fasciae (as in Fig. 16A) or with multiple maculae, irregular in shape (as in Fig. 31A); males and females not dimorphic with female elytron without subapical lateral lobe	**15**
15(14)	Elytral pattern complex, fasciate (Fig. 16A); ventral surface evenly brown	***Hydrodessus fasciatus* sp. n.**
–	Elytral pattern simpler, with maculae subbasally near suture and lateral margin, submedially from lateral margin to near suture, and apically (as in Fig. 31A); ventral surface testaceous or yellow	**16**
16(15)	Prosternal process shallowly depressed medially (Fig. 31C); medial portion of metacoxae (between metacoxal lines) with shallow longitudinal channels which are unmargined (Fig. 31C); dorsal pattern maculate (Fig. 31A)	***Hydrodessus siolii* J. Balfour-Browne**
–	Prosternal process deeply depressed medially; medial portion of metacoxae with distinct longitudinal channels which are margined; dorsal pattern fasciate (Fig. 3)	***Hydrodessus nanayensis* Spangler**
17(2)	Posterior apices of metaventrite carinae located well mediad of anterior apices of metacoxal lines (Fig. 9C)	**18**
–	Posterior apices of metaventrite carinae near anterior apices of metacoxal lines, at most slightly mediad, but generally metaventrite carinae and metacoxal lines approximately continuous (Fig. 35C)	**20**
18(17)	Body elongate, slender (TL/GW = 2.3–2.4); apices of elytra together pointed and slightly, but distinctly dehiscent (Fig. 9A)	***Hydrodessus biguttatus* (Guignot)**
–	Body elongate, but generally somewhat more robust (TL/GW = 2.1–2.3); apices of elytra rounded or broadly pointed but not dehiscent	**19**
19(18)	Overall length longer (TL > 3.5 mm)	***Hydrodessus bimaculatus* sp. n.**
–	Overall length shorter (TL < 3.5mm)	***Hydrodessus disjunctus* sp. n.**
20(17)	Lateral elytral carina extending nearly to elytral apex (Fig. 35B); pronotum with anterolateral margins strongly angulate in many specimens (Fig. 35A1)	***Hydrodessus angularis* Young**
–	Lateral elytral carina not extending nearly to elytral apex (Fig. 18B), at most extending a little past half elytral length; pronotum with anterolateral angles evenly curved	**21**
21(20)	Prosternal process relatively slender (length/width > 2), lateral margins abruptly narrowed medially (Fig. 18C)	***Hydrodessus jethoeae* Makhan**
–	Prosternal process broad (length/width < 2), lateral margins not abruptly narrowed medially, instead process broad throughout length (Fig. 24C)	**22**
22(21)	Metaventral platform strongly constricted, length of metaventral platform long compared with narrowest distance between carinae immediately posteriad to mesocoxae (length/width of constriction > 5, greatest width/width of constriction > 2.5) (Fig. 24C)	**23**
–	Metaventral platfrom not strongly constricted, length of metaventral platform shorter compared with narrowest distance between carinae immediately posteriad to mesocoxae (length/width of constriction < 5, greatest width/width of constriction < 2.5) (Fig. 14C)	**26**
23(22)	With distinctive maculae on elytra subbasally, subapically, and apically, basal macula distinct, transverse, extending nearly to suture (Fig. 24A)	***Hydrodessus maculatus* sp. n.**
–	With elytral maculae indistinct, if present, mainly limited to subapical and apical indistinct pale areas, subbasal area of elytra of some specimens with indistinct, vague pale area	**24**
24(23)	Pro- and mesotibia slender, without subapical emargination along dorsal margin (Fig. 7B)	***Hydrodessus tenuatus* sp. n.**
–	Pro- and mesotibia broad, with subapical emargination along dorsal margin (Fig. 7A)	**25**
25(24)	Length > 3.0 mm	***Hydrodessus latotibialis* sp. n.**
–	Length < 3.0 mm	***Hydrodessus phyllisae* Spangler**
26(22)	Metacoxal lines broadly divergent anteriorly, approximately continuous with metaventrite/ metacoxal suture (Fig. 14C)	***Hydrodessus continuus* sp. n.**
–	Metacoxal lines divergent or not, but intersecting metaventrite/metacoxal suture at distinct angle (Fig. 13C)	**27**
27(26)	Greatest width of pronotum relatively narrow with respect to greatest width across elytra (EW/PW > 1.3) (Fig. 13A); dorsal surface with moderately distinct, but shallow, longitudinal grooves, best observed with oblique lighting (Fig. 13A)	***Hydrodessus concolorans* sp. n.**
–	Greatest width of pronotum relatively broad with respect to greatest width across elytra (EW/PW < 1.2) (Fig. 26A); dorsal surface without grooves (Fig. 26A)	**28**
28(27)	Prosternal process anteriorly with distinctly projecting lateral lobes, abruptly constricted medially (Fig. 11C)	***Hydrodessus brasiliensis* (Guignot)**
–	Prosternal process with lateral margins approximately continuously curved, without prominent lobes, not constricted (Fig. 26C)	***Hydrodessus octospilus* (Guignot)**

**Figures 1–7.**
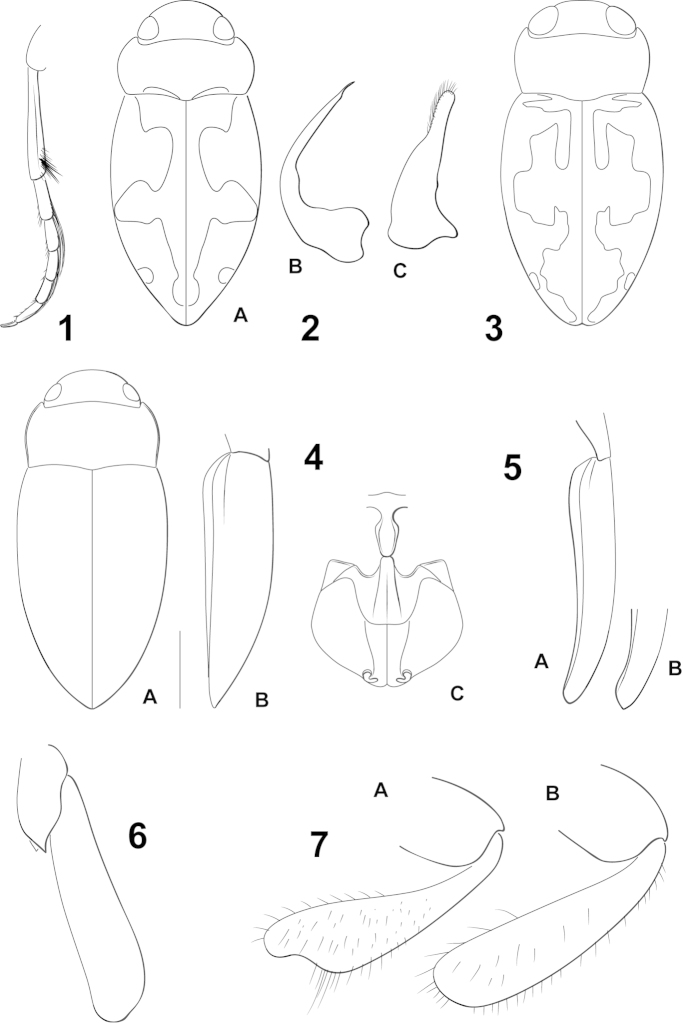
*Hydrodessus* species. **1** *Hydrodessus keithi*, left metathoracic leg, posterior aspect **2** *Hydrodessus amazonensis*, redrawn from Spangler (1966) **A** dorsal habitus **B** male median lobe, right lateral aspect C male right lateral lobe, right lateral aspect **3** *Hydrodessus nanayensis*, dorsal habitus, redrawn from Spangler (1966) **4** *Hydrodessus pereirai* **A** dorsal aspect **B** lateral aspect C prosternal process, mesoventrite, mesocoxae **5** *Hydrodessus keithi*, elytra, lateral aspect **A** male **B** female **6** *Hydrodessus biguttatus*, right metatrochanter and metafemur, anterior aspect **7** *Hydrodessus*, left protibia, anterior aspect **A** *Hydrodessus phyllisae* **B** *Hydrodessus tenuatus*. Scale bars = 1.0 mm for **4A**, **B** only.

**Figures 8–10.**
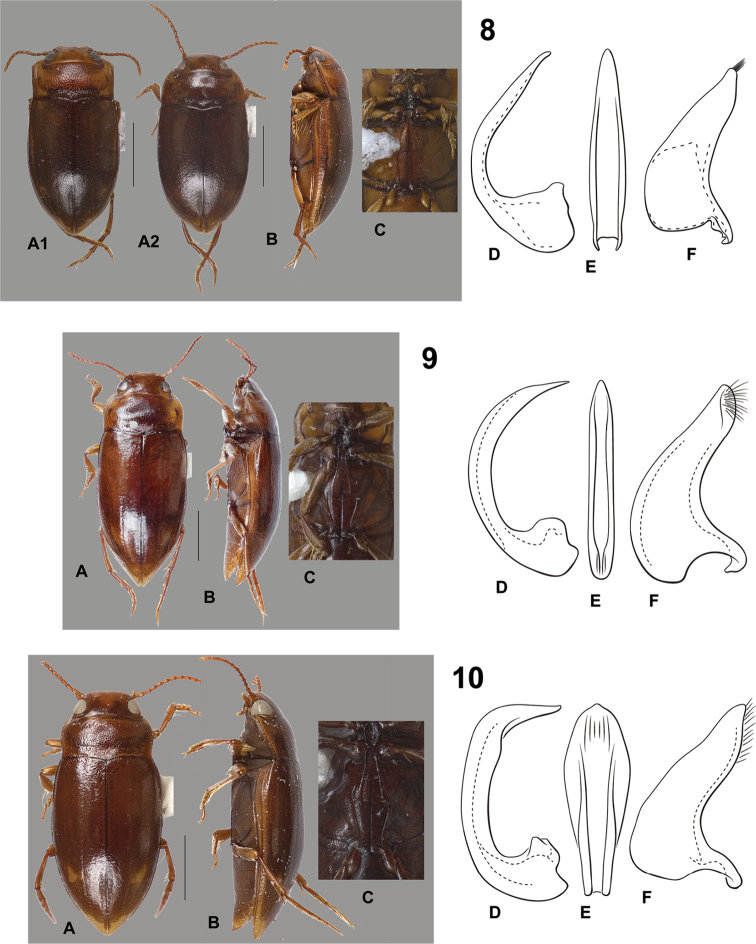
*Hydrodessus* species. **A** dorsal habitus **B** lateral habitus **C** ventral surfaces **D** male median lobe, right lateral aspect **E** male median lobe, ventral aspect **F** male right lateral lobe, right lateral aspect **8** *Hydrodessus angularis*: **A1** dorsal habitus of specimen with strongly angulate pronotum: **A2** dorsal habitus of specimens with less angulate pronotum **9** *Hydrodessus biguttatus* **10** *Hydrodessus bimaculatus*. Scale bars = 1.0 mm for **A** and **B** only.

**Figures 11–13.**
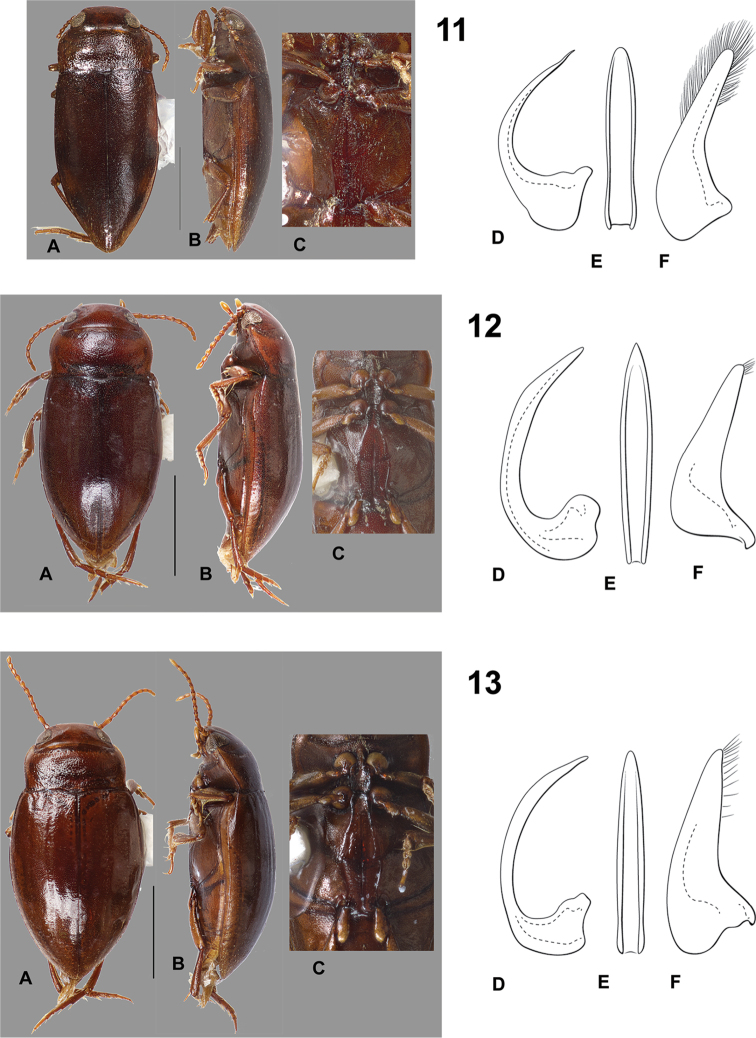
*Hydrodessus* species. **A** dorsal habitus **B** lateral habitus **C** ventral surfaces **D** male median lobe, right lateral aspect **E** male median lobe, ventral aspect **F** male right lateral lobe, right lateral aspect **11** *Hydrodessus brasiliensis* **12** *Hydrodessus brevis* **13** *Hydrodessus concolorans*. Scale bars = 1.0 mm for **A** and **B** only.

**Figures 14–16.**
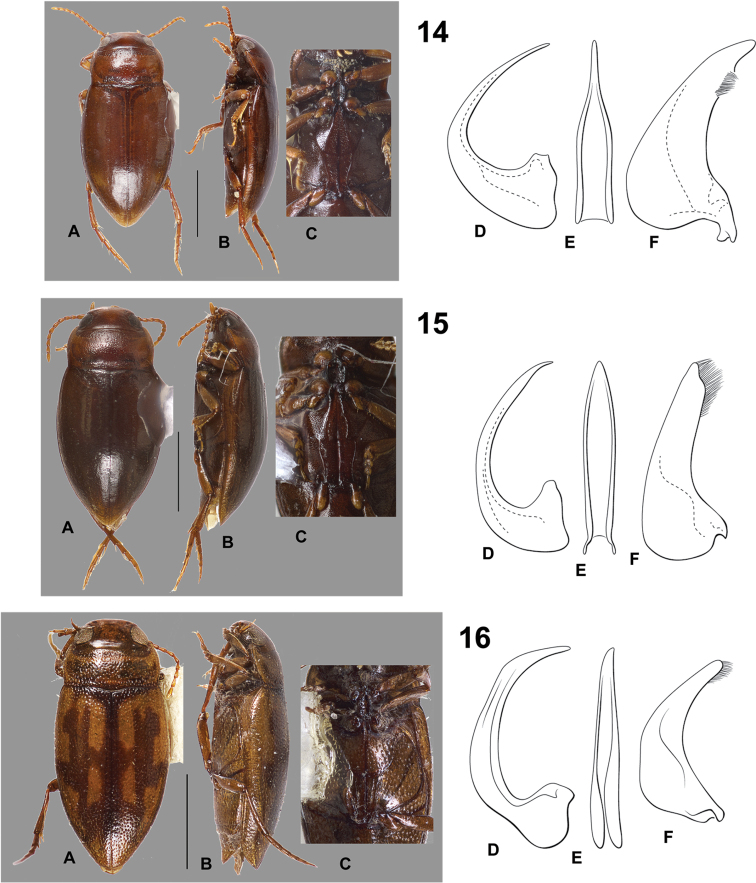
*Hydrodessus* species. **A** dorsal habitus **B** lateral habitus **C** ventral surfaces **D** male median lobe, right lateral aspect **E** male median lobe, ventral aspect **F** male right lateral lobe, right lateral aspect **14** *Hydrodessus continuus* **15** *Hydrodessus disjunctus* **16** *Hydrodessus fasciatus*. Scale bars = 1.0 mm for **A** and **B** only.

**Figures 17–19.**
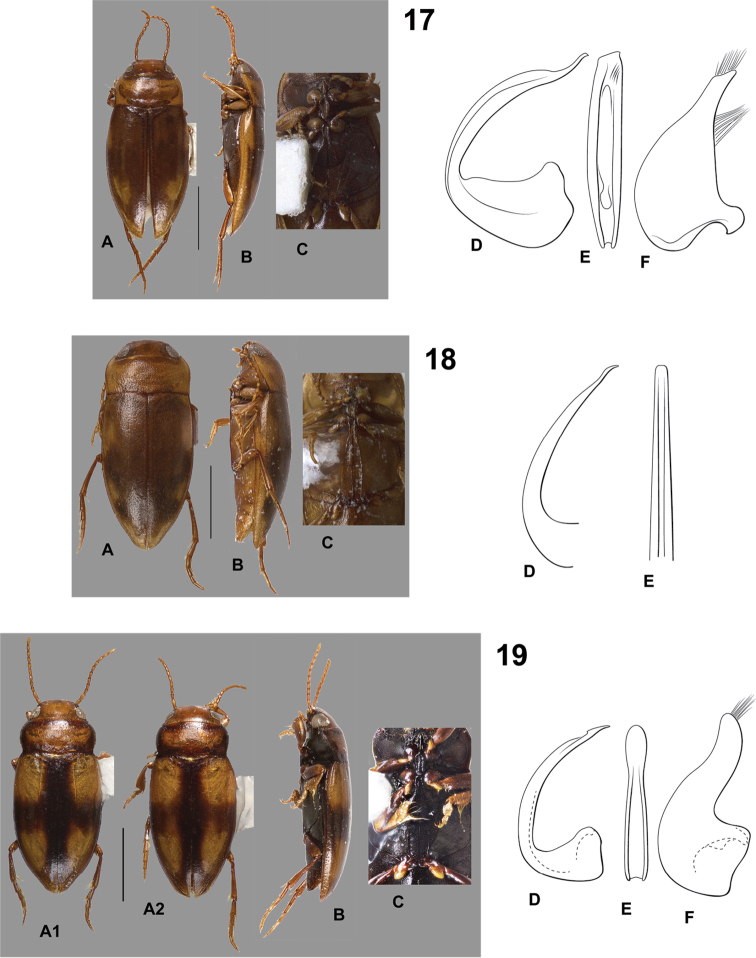
*Hydrodessus* species. **A** dorsal habitus **B** lateral habitus **C** ventral surfaces **D** male median lobe, right lateral aspect **E** male median lobe, ventral aspect **F** male right lateral lobe, right lateral aspect **17** *Hydrodessus imparilis* **18** *Hydrodessus jethoeae* **19** *Hydrodessus keithi* **A1** male **A2** female. Scale bars = 1.0 mm for **A** and **B** only.

**Figures 20–22.**
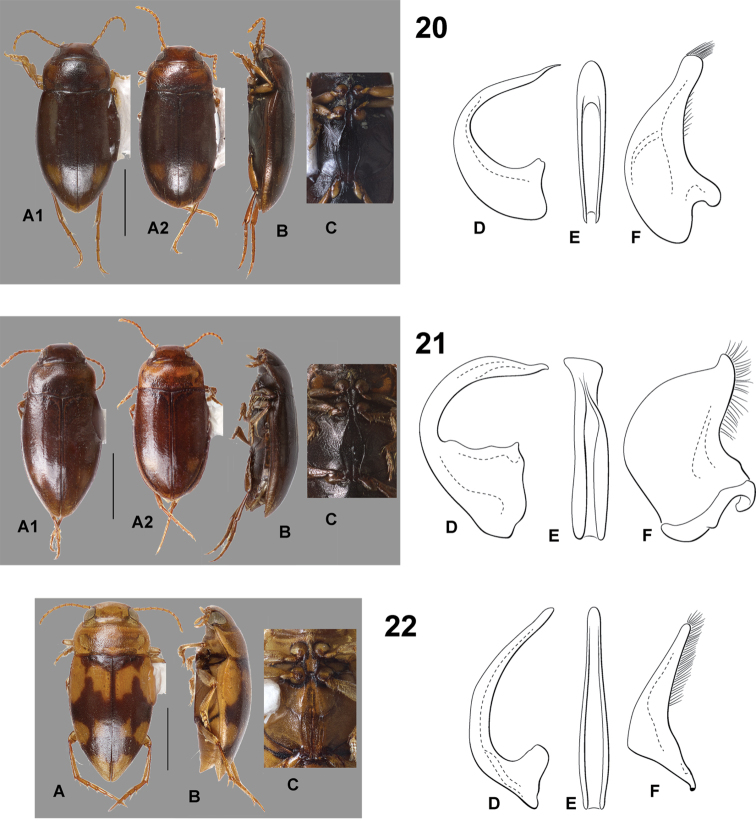
*Hydrodessus* species. **A** dorsal habitus **B** lateral habitus **C** ventral surfaces **D** male median lobe, right lateral aspect **E** male median lobe, ventral aspect **F** male right lateral lobe, right lateral aspect **20** *Hydrodessus kurti* **A1** male **A2** female **21** *Hydrodessus kylei* **A1** male **A2** female **22** *Hydrodessus laetus*. Scale bars = 1.0 mm for **A** and **B** only.

**Figures 23–25.**
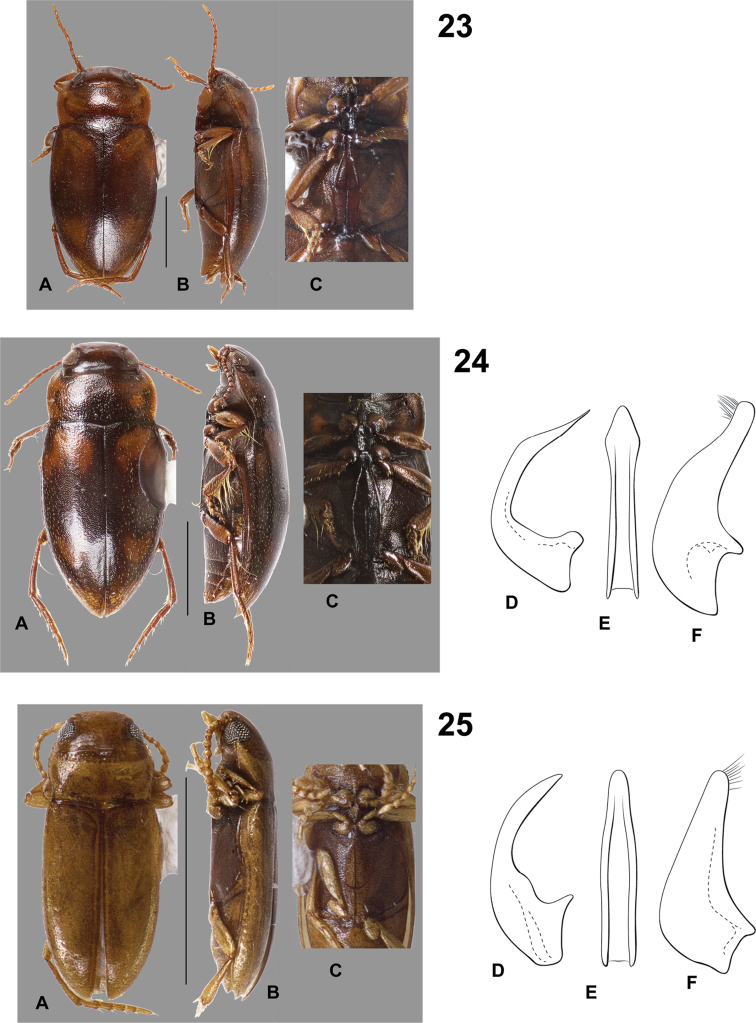
*Hydrodessus* species. **A** dorsal habitus **B** lateral habitus **C** ventral surfaces **D** male median lobe, right lateral aspect **E** male median lobe, ventral aspect **F** male right lateral lobe, right lateral aspect **23** *Hydrodessus latotibialis* **24** *Hydrodessus maculatus* **25** *Hydrodessus morsus*. Scale bars = 1.0 mm for **A** and **B** only.

**Figures 26–28.**
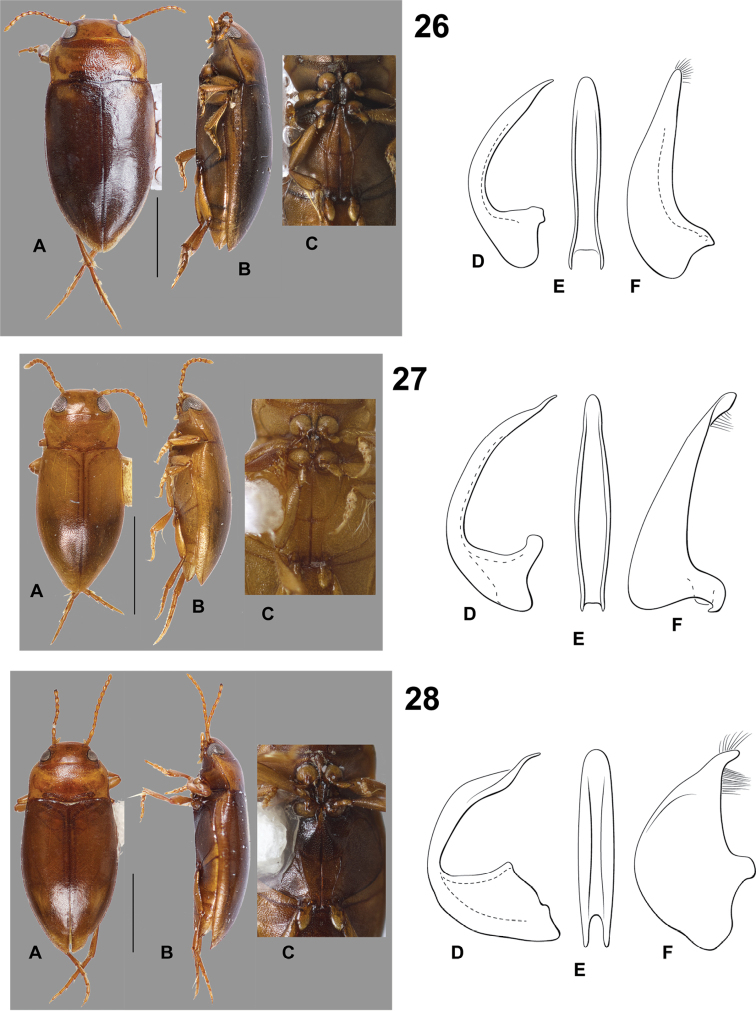
*Hydrodessus* species. **A** dorsal habitus **B** lateral habitus **C** ventral surfaces **D** male median lobe, right lateral aspect **E** male median lobe, ventral aspect **F** male right lateral lobe, right lateral aspect **26** *Hydrodessus octospilus* **27** *Hydrodessus palus* **28** *Hydrodessus peloteretes*. Scale bars = 1.0 mm for **A** and **B** only.

**Figures 29–31.**
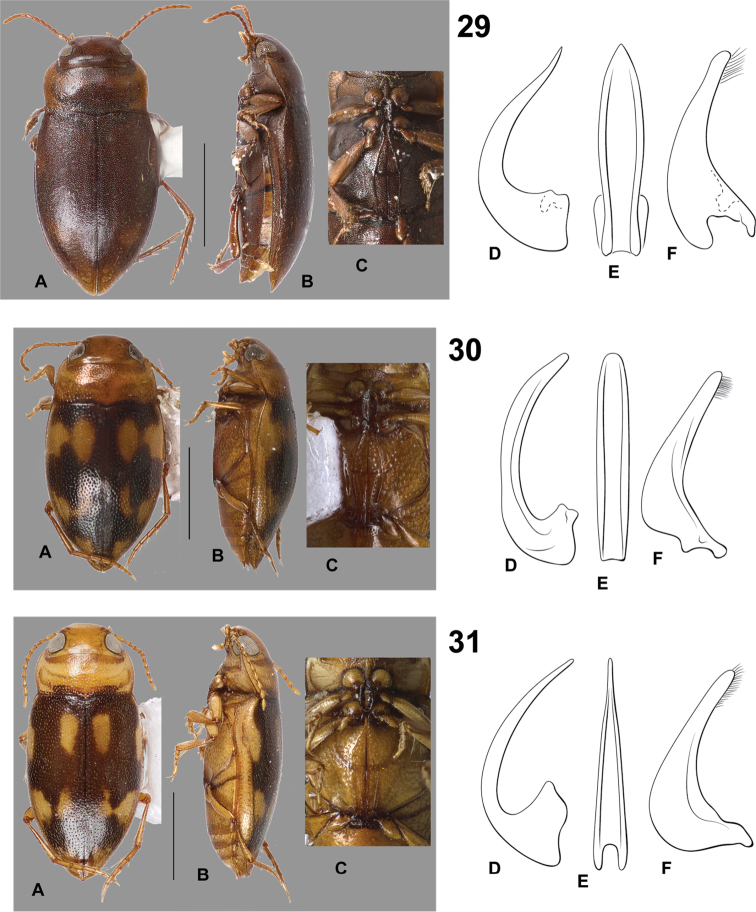
*Hydrodessus* species. **A** dorsal habitus **B** lateral habitus **C** ventral surfaces **D** male median lobe, right lateral aspect **E** male median lobe, ventral aspect **F** male right lateral lobe, right lateral aspect **29** *Hydrodessus phyllisae* **30** *Hydrodessus rattanae* **31** *Hydrodessus siolii*. Scale bars = 1.0 mm for **A** and **B** only.

**Figures 32–34.**
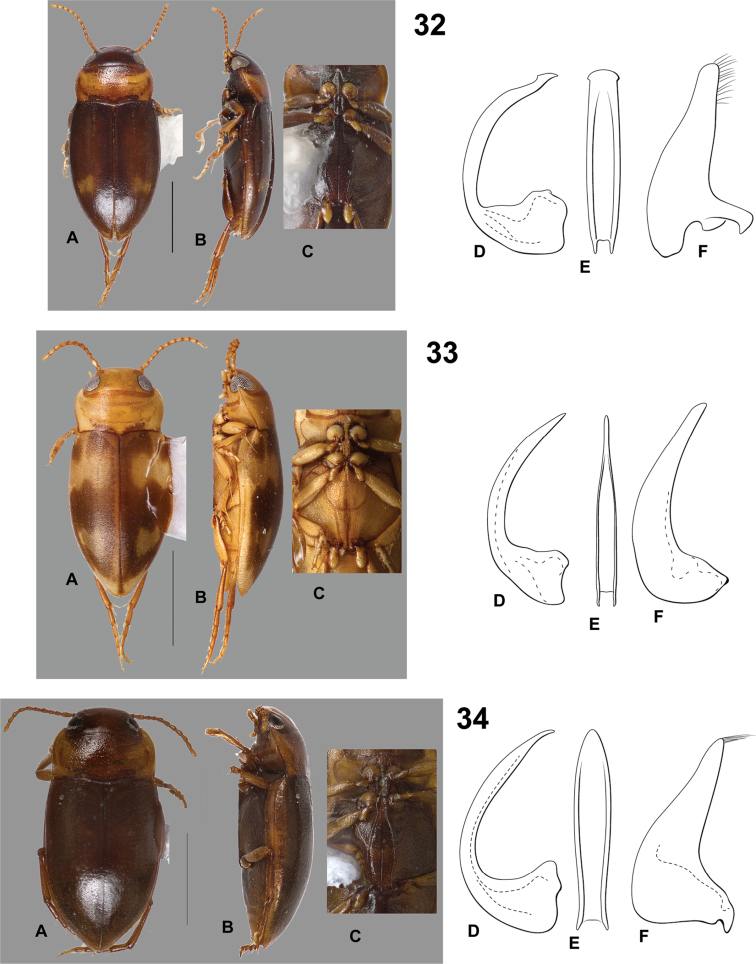
*Hydrodessus* species. **A** dorsal habitus **B** lateral habitus **C** ventral surfaces **D** male median lobe, right lateral aspect **E** male median lobe, ventral aspect **F** male right lateral lobe, right lateral aspect **32** *Hydrodessus spanus* **33** *Hydrodessus surinamensis* **34** *Hydrodessus tenuatus*. Scale bars = 1.0 mm for **A** and **B** only.

**Figures 35–41.**
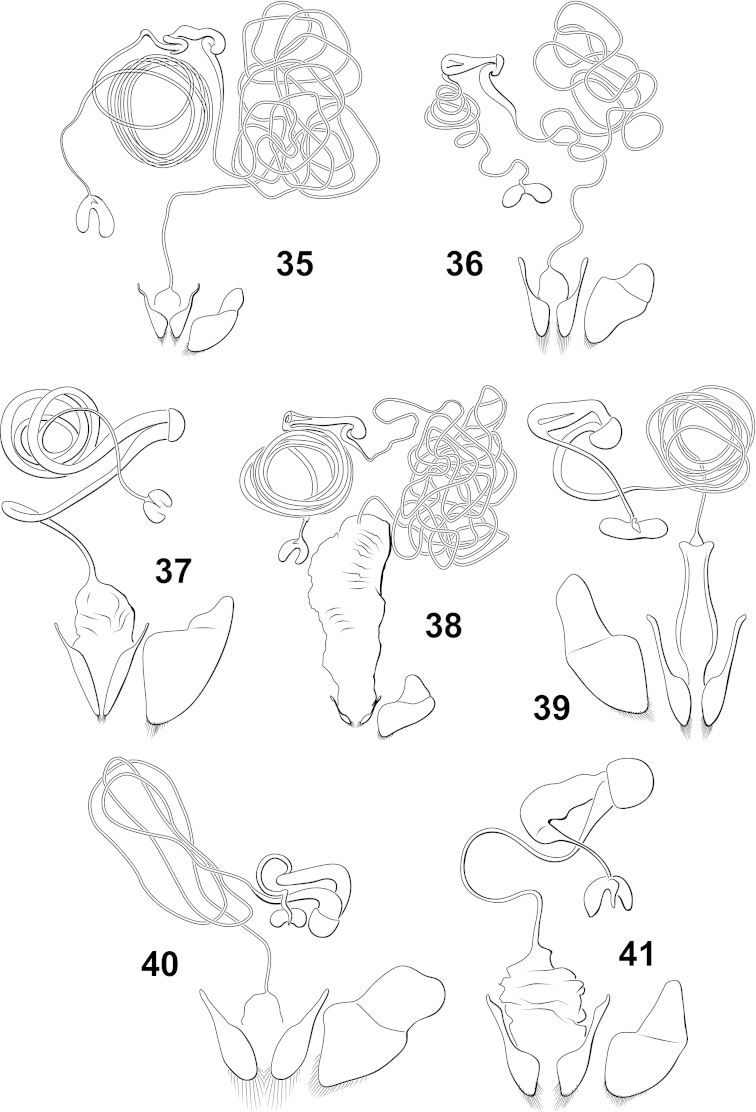
*Hydrodessus* species, female reproductive tract, ventral aspect. **35** *Hydrodessus angularis* **36** *Hydrodessus concolorans* **37** *Hydrodessus keithi* **38** *Hydrodessus kylei* **39** *Hydrodessus laetus* **40** *Hydrodessus maculatus* **41** *Hydrodessus phyllisae*.

### Hydrodessus amazonensis

Taxon classificationAnimaliaColeopteraDytiscidae

Spangler, 1966

Figs 2 , 44

Hydrodessus amazonensis Spangler, 1966: 380; Young 1969: 2; 1970: 157; Spangler 1985: 89; Biström 1988: 37; Nilsson 2001: 236.

#### Type locality.

Peru, near Ituitos, from the Amazonas.

#### Diagnosis.

This species is difficult to diagnose from others since specimens were not available for examination, but based on the description and illustrations by Spangler (1966) the species is elongate with broadly curved lateral pronotal margins (Fig. 2A), the elytra are patterned with testaceous and dark reddish-brown maculae (Fig. 2B), and the lateral elytral carina extend about 1/3 × length of elytron. The male genitalia in lateral aspect were illustrated by Spangler (1966). The median lobe is elongate triangular basally, relatively evenly curved medially with the apical portion straight and slender and the apex abruptly constricted and extremely slender and pointed (Fig. 2B). The lateral lobe is moderately narrow with the dorsal margin straight for most of its length and the apex rounded (Fig. 2C). The overall shape, color pattern and male genitalia should allow for specimens to be identified in the future.

#### Description.

*Measurements*. TL = 2.85 mm, GW = 1.25 mm. Body elongate, apically pointed, lateral outline strongly discontinous between pronotum and elytron (Fig. 2A).

*Coloration* (Fig. 2A). Head and pronotum testaceous. Elytra testaceous except dark reddish-brown medial stripe along suture, one incomplete transverse band basally, one complete transverse band medially, and a small lateral macula at apical 1/5 (Fig. 2A). Antennae, palps legs, and venter testaceous.

*Sculpture and structure*. Head finely, densely punctate, punctures separated by 1 × puncture diameter or less; anterior clypeal margin arcuately emarginate; labrum finely, densely punctate and finely alutaceous, margin narrowly emarginate; anterior margin fringed with setae. Pronotum broadly rounded, widest anterior of middle (Fig. 2A); fine lateral bead present throughout length; surface densely, moderately coarsely punctate, punctures larger than on head, separated by <1 × puncture diameter. Elytra elongate, apically pointed (Fig. 2A); lateral carina distinctive, extending about 1/3 length of elytron; surface microreticulate, appearing granulose and with few, fine punctures similar to pronotum. Prosternal process very slender between procoxae, apical portion 2 × width between procoxae, weakly concave longitudinally. Metaventrite microreticulate, granulose. Legs finely granulose; pro- and mesotibiae moderately broad; metatrochanter swollen apically; metacoxa microreticulate. Abdomen microreticulate, granulose.

*Male genitalia*. Median lobe in lateral aspect strongly curved medially, apical portion slightly curved, abruptly narrowed along dorsal margin subapically, apex narrowly pointed and slightly curved (Fig. 2B); lateral lobe in lateral aspect moderately broad in basal portion, apex slightly narrowed and straight to broadly rounded apex, with series of setae along medial surface apically (Fig. 2C).

*Female genitalia*. Females not described by Spangler (1966).

*Sexual dimorphism*. Male pro- and mesotarsi I–III more broadly expanded than female and ventrally with several large adhesive setae; female with sublateral carina absent basally.

*Variation*. According to Spangler (1966), specimens differ somewhat in size and coloration with some specimens having the dark coloration reduced or more enlarged. The presence of the sublateral carina is also variable, and it is absent in some specimens.

#### Distribution.

This species is known only from the type locality near Iquitos, “from the Amazonas,” Peru. (Fig. 44).

**Figures 42–47.**
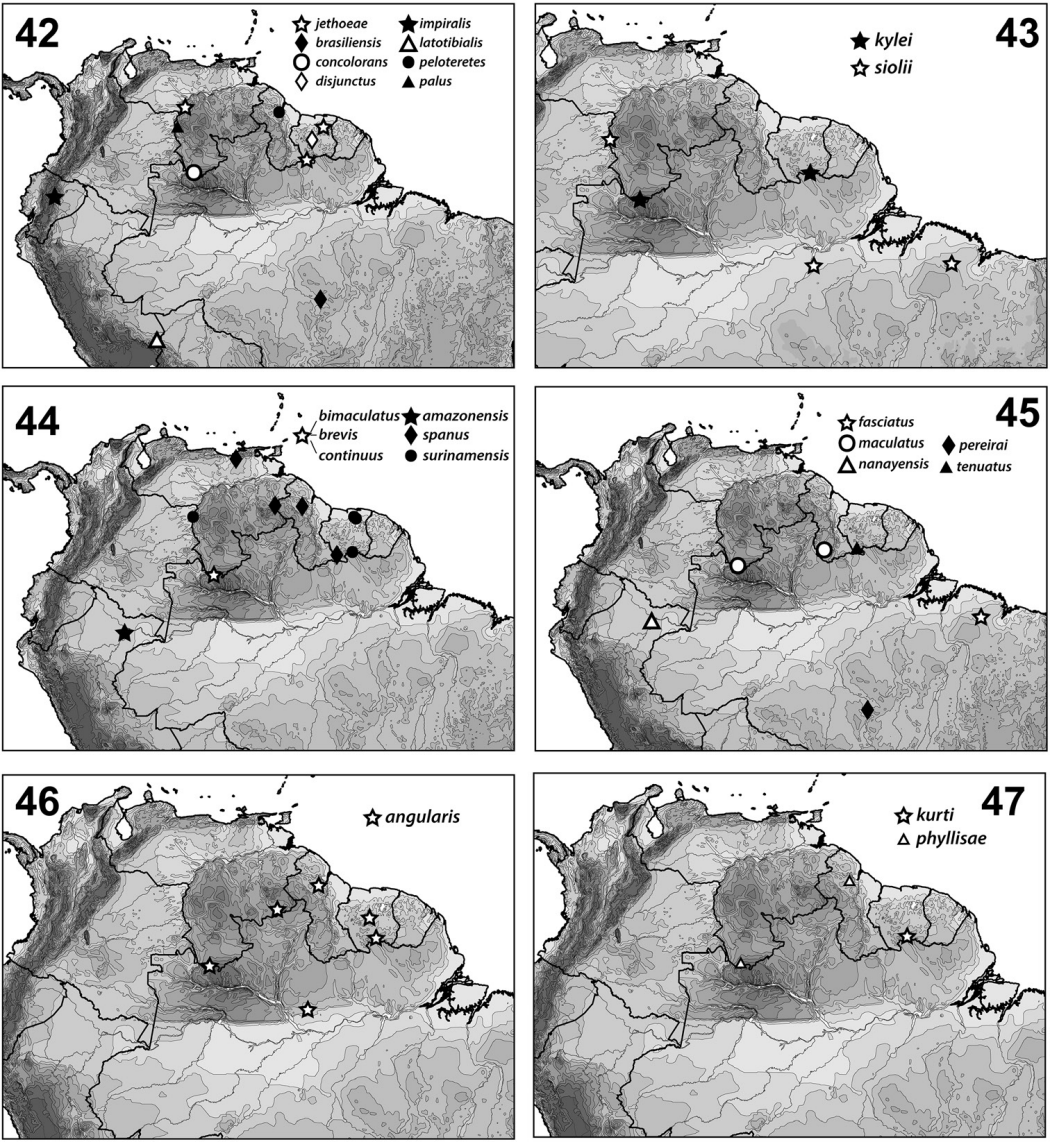
*Hydrodessus* species, distributions.

#### Habitat.

Nothing is known of the natural history of this species.

#### Discussion.

The specimens on which this species (and *Hydrodessus nanayensis* Spangler) were based were collected during the Catherwood Foundation expedition to Peru. The type material was not found in either the ANSP, where Spangler indicated the holotype was deposited (J. Weintraub, pers. comm.), the MZCZ (where many ANSP Coleoptera types were sent), or the USNM (where Spangler was working). Illustrations of the habitus and lateral aspect of the male genitalia are provided (redrawn in Fig. 2B,C), and the description of the species is extensive, though it excludes a number of important diagnostic features. The description presented here is based on Spangler’s (1966) description and his figures and later keys (Spangler 1985; Young 1970). The extremely curved lateral margins of the pronotum, the distinctive color pattern on the elytron, and the shapes of the male genitalia are distinctive (Figs 2B, C), but *Hydrodessus amazonensis* does not appear to correspond to any specimens examined during this study. Spangler (1966) indicates that the fine sublateral elytral carina is present in only two specimens he examined, the holotype and one other, suggesting that perhaps the series was mixed.

#### Specimens.

No specimens were examined of this species, and the treatement here is based on the description by Spangler (1966).

### Hydrodessus angularis

Taxon classificationAnimaliaColeopteraDytiscidae

Young, 1970

Figs 8 , 35 , 46

Hydrodessus angularis Young, 1970: 155; Spangler 1985: 88; Biström 1988: 37; Nilsson 2001: 236.

#### Type locality.

Suriname, Carolina Creek, 10km S Zanderij.

#### Diagnosis.

This is a very distinctive species which is dorsally nearly concolorous red (Fig. 8A1,A2) except some specimens have the head and pronotum lighter orange and some specimens have poorly defined pale regions basally and subapically on the elytron. The lateral elytral carina is sharp and long, extending more than 3/4 length of the elytron (Fig. 8B). Specimens are robust with the lateral margins broadly rounded (Fig. 8A). Many specimens (not all) have the anterolateral angles of the pronotum conspicuously flattened and produced laterally into distinct, broad angle (Fig. 8A1). The anterior clypeal margin is beaded and somewhat projecting. The prosternal process is broadly quadrate and apically broadly trunctate (Fig. 8C). The metaventrite carinae are distinctive and posteriorly divergent (Fig. 8C). The male median lobe is basally triangular with the apical portion curved basally and apically approximately linear with the apex slight curved dorsad and narrowly rounded (Fig. 8D). The median lobe in ventral aspect is moderately broad with the lateral margins broadest submedially and evenly convergent to broadly pointed apex (Fig. 8E). The lateral lobe is very broad with the lateral margins approximately convergent to rounded apex (Fig. 8F).

#### Description.

*Measurements*. TL = 2.9–3.2 mm, GW = 1.4–1.5 mm, PW = 1.3 mm, HW = 0.9 mm, EW = 0.5–0.6 mm, TL/GW = 2.0–2.1, HW/EW = 1.6–1.7. Body robust, broad, lateral margin only slightly discontinous between pronotum and elytron (Fig. 8A).

*Coloration* (Fig. 8A). Head and pronotum yellow. Elytra brown to yellow or red-brown, with subapical, small, triangular macula and apex yellow in many specimens with other specimens evenly brown. Antennae and palps yellow to yellow brown. Legs yellow. Venter yellow to orange.

*Sculpture and structure*. Head broad, relatively short, apically with clypeal margin projecting, medially broadly truncate and finely beaded; surface with inconspicuous, fine punctures; eyes moderately large. Pronotum with lateral margins broadly curved, greatest width near middle (Fig. 8A), some specimens with lateral margins more strongly flattened and distinctly angulate anterolaterally (Fig. 8A1); lateral bead fine anteriorly, slightly expanded near posterolateral angle; surface shiny with fine punctation medially, irregularly punctate to rugulose laterally. Elytra broad, lateral margins subparallel in anterior half; lateral carinae very well developed and prominent, extending more than ¾ length of elytron (Fig. 8C); elytral apex with slight constriction subapically; surface covered with fine punctures. Prosternum medially slightly carinate; prosternal process very broad, subquadrate, lateral margins subparallel, but widest at anterior margin, apex broadly truncate to broadly concave, medially strongly impressed (Fig. 8C). Metaventrite with metasternal process well developed, apically truncate, subapically constricted, medial surface slightly excavated, carinae well-developed, long, divergent posteriorly across metasternum, ending near anterior ends of metacoxal lines (Fig. 8C); Metaventrite covered with fine punctures. Legs with surfaces covered with fine punctures; pro- and mesotibiae moderately broad; metatibia with posteroapical brush of setae; metacoxa covered with fine punctures; metacoxal lines broadly separated, subparallel, but slightly curved and anteriorly somewhat divergent (Fig. 8C). Abdomen covered with fine punctures.

*Male genitalia*. Median lobe bilaterally symmetrical, in lateral aspect strongly curved medially, with base broad and subtriangular, apical portion more straight, with dorsal and ventral margins slightly expanded, narrowing to slender, narrowly rounded apex (Fig. 8D); in ventral aspect broad, lateral margins broadly curved, apically evenly convergent to pointed apex (Fig. 8E). Lateral lobe very broad basally, elongate, margins approximately evenly convergent to narrowly rounded apex which has small cluster of setae (Fig. 8F).

*Female genitalia*. Gonocoxosternite broad, posterolateral margin broadly curved, medial margin slightly concave, anterior portion small, lobate (Fig. 35). Gonocoxa with apical portion broadly triangular, apically narrowly rounded, anterior apodeme as long as apical portion and sinuate (Fig. 35). Bursa short and broad; spermathecal duct extremely long and slender, expanding near receptacle which is small; spermatheca elongate and twisted, without spermathecal spine; fertilization duct extremely long, slender, and coiled (Fig. 35).

*Sexual dimorphism*. Male pro- and mesotarsi I–III more broadly expanded than female and ventrally with several large adhesive setae.

*Variation*. The apical elytral maculae are indistinct in many specimens and are most conspicuous in teneral specimens. The most conspicuous variation is the degree of angulation of the lateral pronotal margins. Individuals from Suriname have the lateral pronotal margins strongly flattened and distinctly angulate (Fig. 8A1). Specimens from farther west, including Venezuela, have the lateral margins less strongly angulate (Fig. 8A2). The Suriname specimens also have the anterior margin of the clypeus more strongly concave that those specimens farther west. The specimens agree in other characters including the shape of the prosternal process, metasternum, and metacoxae and the shape of the male genitalia such that the variation in the lateral pronotal margin is here regarded as intraspecific variation.

#### Distribution.

*Hydrodessus angularis* is known from Amazonas, Brazil through Guyana and Suriname to southern Venezuela (Fig. 46).

#### Habitat.

Specimens have been collected from along a river margin, in a large sandy creek, a muddy oxbow pond, in detrital pools by a forest stream, and from lights at night. The species appears to be mainly associated with margins of forest rivers.

#### Discussion.

Although the holotype of this species (in Rijksmuseum van Natuurlijke Historie, Leiden) was not examined, there is little doubt as to the identity of the species. That said, many specimens do not have the anterior angles of the pronotum nearly as angulate as others. In some of these specimens the anterior margin of the clypeus is not as strongly margined. The more angulate specimens are generally found in the eastern part of the range. The male genitalia are identical, and other features, such as the well-marked lateral elytral carina, the shape of the prosternal process, metaventrite carinae, and metacoxae are also the same. Even so, a greater sampling may eventually reveal that more than one species is actually involved.

#### Specimens.

Holotype not examined. Other non-type specimens examined (84 total): **Brazil**; Amazonas, Ig.Tarumazinho, 46km N Manaus, 2.339°S 60.029°W, 6 Feb 1979, O. Flint (1, USNM). **Guyana**; Mazaruni-Potaro District, Takutu Mountains, 6.25°N, 59.083°W, 18 Dec 1983, blacklight forest clearing near streams, Earthwatch Research Expedition, P.J Spangler and W.E. Steiner (2, USNM). **Suriname**; Sipaliwini District, Camp 1, Upper Palumeu, 2.477°N, 55.629°W, 14 Mar 2012, large sandy creek, 275m, A. Short (4, KUNHM); Sipaliwini District, CSNR: near Kappel airstrip, 3.792°N, 56.150°W, 12 Aug 2013, uv light trap, 320m, A.E.Z. Short (7, KUNHM). **Venezuela**; Territorio Federal Amazonas, Cerro de la Neblina, basecamp, 0.833°N, 66.167°W, 21 Feb 1985, muddy oxbow pond, rainforest clearing, 140m, W.E. Steiner (6, USNM); Territorio Federal Amazonas, Cerro de la Neblina, basecamp, 0.833°N, 66.167°W, 20 Feb 2985, seined from rocks in rapids of Rio Baria, 140m, P.J. Spangler, P.M. Spangler, R. Faitoute and W. Steiner (23, USNM); Territorio Federal Amazonas, Cerro de la Neblina, basecamp, 0.833°N, 66.167°W, 20 Feb 2985, netted along margins of Rio Baria, 140m, P.J. Spangler, P.M. Spangler, R. Faitoute and W. Steiner (40, USNM); Bolivar State, Gran Sabana, Pauji, Esmeraldes, 4.471°N, 61.593°W, 16 Jul 2010, detrital pools by forested stream, 867m, Short, Tellez and Arias (1, KUNHM). KUNHM catalog numbers in Table 1.

**Table 1.** SEMC (University of Kansas) accession numbers for certain *Hydrodessus* specimens included in revision.

Species	KUNHM accession numbers
*Hydrodessus angularis*	SEMC0908225, SEMC0930584, SEMC0930585, SEMC1088259, SEMC1088302, SEMC1088325, SEMC1088329, SEMC1089613, SEMC1089618, SEMC1234318, SEMC1234323, SEMC1234327
*Hydrodessus biguttatus*	SEMC0913238
*Hydrodessus disjunctus*	SEMC1080468, SEMC1080471
*Hydrodessus jethoeae*	SEMC0854749, SEMC0915510
*Hydrodessus kurti*	SEMC1088337 , SEMC1088338, SEMC1088339, SEMC1088342, SEMC1088346, SEMC1088347, SEMC1088351
*Hydrodessus kylei*	SEMC0915690, SEMC1088262, SEMC1088263, SEMC1088284, SEMC1088286, SEMC1088295, SEMC1088296, SEMC1088298, SEMC1088303, SEMC1088316, SEMC1088321, SEMC1088322, SEMC1088328, SEMC1088330, SEMC1088331, SEMC1088332, SEMC1088334, SEMC1088335, SEMC1088344
*Hydrodessus maculatus*	SEMC0964975, SEMC0964987
*Hydrodessus octospilus*	KUNHM SEMC0964970, KUNHM SEMC0964971, KUNHM SEMC0964975, KUNHM SEMC0964984, KUNHM SEMC0964985, KUNHM SEMC0964986, KUNHM SEMC0964989, KUNHM SEMC0964991
*Hydrodessus palus*	SM0842821, SM0842840
*Hydrodessus rattanae*	SEMC1080472, SEMC1080473, SEMC1080474, SEMC1080475, SEMC1080476
*Hydrodessus siolii*	SM0842832, SM0843017, SM0843053, SM0843078, SM0843079, SM0843080, SM0843127, SM0843127, SM0843130, SM0843131, SM0843138, SM0843142, SM0843143, SM0843144, SM0843146, SM0843151, SM0843153, SM0843166, SM0843170, SM0843172, SM0843175, SM0843176, SM0843179, SM0843186, SM0843187, SM0843188, SM0843189, SM0843195, SM0843197, SM0843198, SM0843199, SM0843200, SM0843201, SM0843202, SM0843203, SM0843227, SM0843228, SM0843229, SM0843245, SM0843246, SM0843247, SM0843276, SM0843306, SM0843308, SM0843309, SM0843312, SM0843316, SM0843317, SM0843318, SM0843320, SM0843327, SM0843329, SM0843337, SM0843338, SM0843340, SM0843347, SM0843348, SM0843354, SM0843355, SM0843357, SM0843359
*Hydrodessus spanus*	MIZA0001487, SEMC0914432
*Hydrodessus surinamensis*	SM0843163, SM0843182, SM0843268, SM0843269, SM0843299
*Hydrodessus surinamensis*	SEMC1088261, SEMC1089221
*Hydrodessus tenuatus*	SEMC0915670

### Hydrodessus biguttatus

Taxon classificationAnimaliaColeopteraDytiscidae

(Guignot, 1957)

Figs 6 , 9 , 49

Brinckius biguttatus Guignot, 1957: 39.
Hydrodessus biguttatus , Young 1969: 2; 1970: 157; Spangler 1985: 88; Biström 1988: 37; Nilsson 2001: 236.
Hydrodessus fragrans Spangler, 1985: 82; Young 1969: 2; Biström 1988: 37; Nilsson 2001: 236. **syn. n.**

#### Type locality.

*Brinckius biguttatus* Guignot: Brazil, Pará State, Cachimbo. *Hydrodessus fragrans* Spangler: Guyana, Mazaruni-Potaro District, Takutu Mountains, 6°15'N 59°5'W.

#### Diagnosis.

This species is elongate and dorsally and ventrally nearly concolorous red, though some specimens have indistinct pale, subtriangular maculae subapically and the apex of the elytron more pale (Fig. 9A). The elytral apices are slightly dehiscent (Fig. 9A). The lateral elytral carinae are somewhat variable from about 1/4–2/5 length of elytron (Fig. 9B). The prosternal process is very broad and broadly excavated medially with the lateral margins subparallel (Fig. 9C). The metaventrite carinae are prominent, not medially constricted and posteriorly somewhat divergent, but the posterior apices are located distinctly mediad of the anterior apices of the metacoxal lines (Fig. 9C). The male median lobe in lateral aspect has the basal portion relatively small, the apical portion is elongate, slender and evenly and broadly curved (Fig. 9D). The apex is elongate and sharply pointed (Fig. 9D). The median lobe in ventral aspect has the margins nearly parallel to the convergent, narrowly rounded apex (Fig. 9E). The lateral lobe is broad, curved medially and apically broadly rounded (Fig. 9F). This species is most similar to *Hydrodessus bimaculatus* and *Hydrodessus disjunctus*. Those species do not have dehiscent elytral apices and the male genitalia are different (see under those species).

#### Description.

*Measurements*. TL = 3.9–4.6 mm, GW = 1.7–2.0 mm, PW = 1.4–1.7 mm, HW = 1.1–1.2 mm, EW = 0.7 mm, TL/GW = 2.3–2.4, HW/EW = 1.6–1.7. Body shape elongate, narrow, lateral outline strongly discontinuous, apically pointed with elytra dehiscent apically (Fig. 9A).

*Coloration* (Fig. 9A). Head and pronotum orange. Elytra red with small lateral pale macula, larger diffuse subtriangular subapical pale macula, and elytral apices yellow. Antennae, palps and legs orange. Venter orange on most surfaces, yellow-brown on mesocoxae and metasternum.

*Sculpture and structure*. Head broad, anterior clypeal margin broadly curved, slightly flattened dorsoventrally; surface covered with minute punctures; eyes large. Pronotum subcordate, widest slightly anterior to middle (Fig. 9A); lateral bead fine and continuous; surface shiny, covered with fine punctures. Elytra long, apices pointed and finely but distinctly dehiscent apically (Fig. 9A); lateral carina distinct, extending about 2/5 length of elytron (Fig. 9B); surface covered with fine punctures. Prosternum medially weakly tectiform and setose; prosternal process very broad, widest at anterior lobes, margins slightly convex, convergent to broadly truncate apex, broadly excavated medially (Fig. 9C). Metaventrite with anterior process prominent, apex trunctate, slightly expanded subapically, carinae distinctive anteriorly, moderately divergent becoming slightly less distinctive and broader posteriorly, converging with posterior margin well mediad of anterior apices of metacoxal lines (Fig. 9C); surface covered with fine punctation. Legs shiny, relatively impunctate; metatibia with distinctive brush of dense, elongate setae on postero-apical surface; pro- and mesotibiae moderately slender; metatrochanter distinctly offset, apically minutely bispinous (Fig. 6); metacoxa evenly covered with fine punctures; metacoxal lines broadly separated, broadly divergent anteriorly (Fig. 9C). Abdomen shiny, evenly covered with fine punctures; apex of VI broadly pointed.

*Male genitalia*. Median lobe bilaterally symmetrical, in lateral aspect broadly and evenly curved to narrow, narrowly rounded apex (Fig. 9D); in ventral aspect nearly parallel-sided throughout most of length, narrow, apically abruptly narrowed to narrowly rounded apex (Fig. 9E). Lateral lobe moderately broad basally, apically gradually narrowed, apex obliquely rounded with dense region of short setae (Fig. 9F).

*Female genitalia*. Not examined.

*Sexual dimorphism*. Male pro- and mesotarsi I–III slightly more broadly expanded than female and ventrally with several large adhesive setae. Some females specimens with fine dorsal microsculpturing which makes surface matte, other females and males dorsally shiny.

*Variation*. Specimens are conspicuously variable in size. There are relatively few specimens available to determine whether there is a geographic component to size variability, and other attributes (male genitalia, etc) do not evidently vary with size. There is some variation in the extent of elytral maculation. Given the variation, it is certainly possible that multiple species are involved, thought the diagnostic characters are consistent across the specimens examined.

#### Distribution.

This species has been collected from Para, Brazil north through Suriname and Guyana to southern Venezuela (Fig. 49).

**Figures 48–51.**
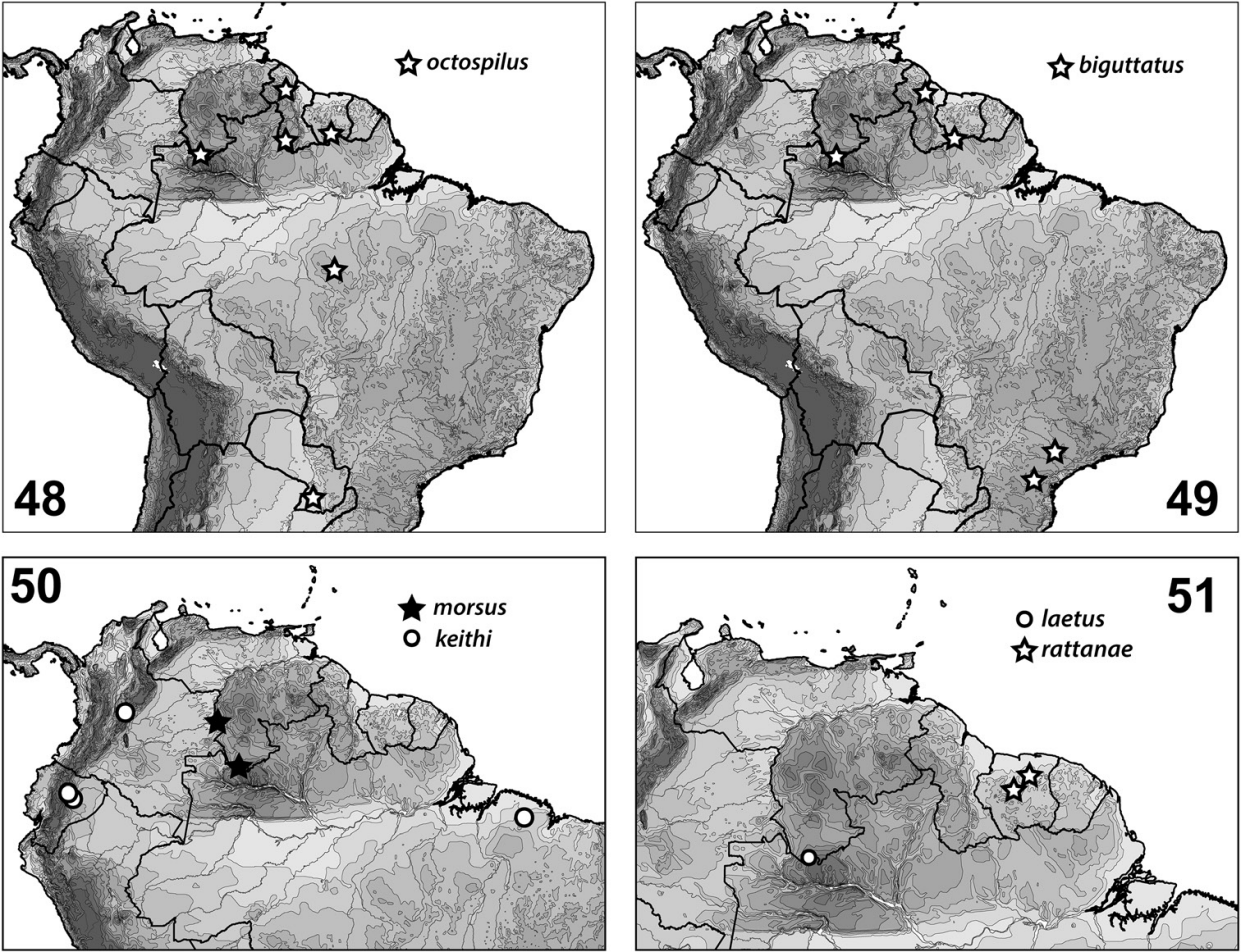
*Hydrodessus* species, distributions.

#### Habitat.

Specimens have been collected from blacklights in tropical forests and from the margins of a river and a flooded forest stream.

#### Discussion.

Although the holotype of *Hydrodessus biguttatus* was not found, a paratype specimen was examined and compared with the holotype and other material of *Hydrodessus fragrans*. The *Hydrodessus biguttatus* paratype is a male, and is dissected, but the genitalia are not with the specimen. Nevertheless, the specimen agrees well with specimens of *Hydrodessus fragrans*. In particular, these specimens all have the apices of the elytra distinctly dehiscent and the apex of the metatrochanter minutely but distinctly bispinous with a small spine at the dorsal apex and a slightly smaller spine at the ventral apex. Spangler (1985) diagnosed *Hydrodessus fragrans* from *Hydrodessus biguttatus* mainly on coloration and punctation, but these differences are well within the typical range of variation of species of *Hydrodessus*. For this reason, *Hydrodessus fragrans* Spangler, 1985 is placed as a junior synonym of *Hydrodessus biguttatus* (Guignot, 1957), **syn. n.**

This species, though widespread, is rarely collected and has not been collected in long series.

#### Specimens.

Holotype of *Hydrodessus biguttatus* not examined. Holotype of *Hydrodessus fragrans* examined, male in USNM labeled, “GUYANA: Mazaruni- Potaro District Takutu Mountains 6°15'N,59°5'W 16 December 1983/ EARTHWATCH Research Expedition: P. J. Spangler & W. E. Steiner Collectors/ At blacklight in forest clearing near streams/ HOLOTYPE *Hydrodessus fragrans* PJ Spangler [red label]/ BLNO 003803 [blue label with black line around margin].”

Other non-type specimens examined (6 specimens): **Brazil**, Para, Cachimba, 25.6°S 49.3°W, 1 Oct 1955, Pereira (1, MZSP, paratype of *Hydrodessus biguttatus*); São Paulo, Dona Antonio, 22.7°S 47.7°W, 14 Mar 1979, C.R. Owen (1, USNM). **Guyana**, Mazaruni-Potaro District, Takutu Mountains, 6.25°N, 59.083°W, 14 Dec 1983, blacklight in forest clearing near streams, P.J. Spangler, W.E. Steiner (2, USNM, including 1 paratype of *Hydrodessus fragrans*). **Suriname**, Sipaliwini District, Camp 1, on Kutari River, 2.175°N, 56.787°W, 22 Aug 2010, flooded forest stream, 228m, Short, Kadosoe (1, KUNHM, SEMC0913238). **Venezuela**, Amazonas, Cerro de la Neblina, basecamp, 0.833°N, 66.167°W, 27 Jan 1985, netted along margins of Rio Baria, 140m, P.J. Spangler, P.M. Spangler, R. Faitoute, W. Steiner (2, USNM).

### Hydrodessus bimaculatus sp. n.

Taxon classificationAnimaliaColeopteraDytiscidae

http://zoobank.org/C75BB071-15C2-4229-9AB1-4A654605D89F

Figs 10 , 44

#### Type locality.

Venezuela, Territoria Federal Amazonas, Cerro de la Neblina, basecamp, 0.833°N, 66.167°W.

#### Diagnosis.

This species is moderately elongate and dorsally and ventrally nearly concolorous red, except with small pale, subtriangular maculae subapically and the apex of the elytron is narrowly pale (Fig. 10A). The elytral apices are not dehiscent (Fig. 10A). The lateral elytral carinae extend about 1/4 length of the elytron (Fig. 10B). The prosternal process is very broad, broadly excavated medially, and slightly broader anteriorly (Fig. 10C). The metaventrite carinae are prominent, not medially constricted and posteriorly somewhat divergent, but the posterior apices are located distinctly mediad of the anterior apices of the metacoxal lines (Fig. 10C). The male median lobe in lateral aspect is relatively small basally with the apical portion slender, linear medially, abruptly curved subapically and with apex linear and narrowed to pointed apex (Fig. 10C). The median lobe in ventral aspect is bilaterally symmetrical and very broadly expanded medially (Fig. 10D). Apically the median lobe is abruptly broadly angulate (Fig. 10D). The lateral lobe is moderately broad, curved basally and apically broadly narrowed to narrowly rounded apex (Fig. 10F). This species is most similar to *Hydrodessus disjunctus* and *Hydrodessus biguttatus*. From *Hydrodessus biguttatus* it differs in the absence of dehiscent elytral apices and the shape of the male genitalia. From *Hydrodessus disjunctus* this species differs in size (*Hydrodessus bimaculatus* are longer, TL > 3.5 mm) and the male genitalia are different.

#### Description.

*Measurements*. TL = 3.8–3.9 mm, GW = 1.5–1.7 mm, PW = 1.4–1.5 mm, HW = 1.1 mm, EW = 0.6 mm, TL/GW = 2.2–2.3, HW/EW = 1.7. Body elongate, apically pointed, lateral margins strongly discontinuous between pronotum and elytron (Fig. 10A).

*Coloration* (Fig. 10A). Head and pronotum red. Elytra red, with diffuse, yellow macula subapically and with apex yellow. Antennae and palpi yellow-red. Legs yellow. Venter red-brown, lighter on epipleuron and apex of abdomen.

*Sculpture and structure.* Head broad; anterior clypeal margin broadly rounded; surface with fine microreticulation and with sparse, indistinct punctures; eyes large. Pronotum cordate, widest anterior to middle; lateral bead fine, continuous along margin; surface with fine microreticulation and punctation variable with some larger and some smaller punctures. Elytra elongate, apically pointed; lateral carina distinctive, extending about 1/4 length of elytron (Fig. 10A); surface covered with fine punctation. Prosternum medially tectiform and setose; prosternal process subquadrate, broad, broadest at anterior laterally-expanded angles, medially strongly impressed, apex broadly truncate (Fig. 10C). Metaventrite with anterior process broad, slightly expanded anteriorly, apically truncate; metasternal carinae distinct, diverging posteriorly (Fig. 10C); surface with fine punctures. Legs with most surfaces covered with fine punctures; pro- and mesotibiae moderately broad; metatibia with posteroapical brush of setae; metatrochanter not strongly offset, apically pointed; metacoxae covered with fine punctures; metacoxal lines moderately distinct, straight and distinctly divergent anteriorly (Fig. 10C). Abdomen covered with fine punctures; ventrite VI rounded with small, spinous, medioapical lobe.

*Male genitalia*. Median lobe bilaterally symmetrical, in lateral aspect strongly curved basally and subapically, straight medially, basal portion small, apical portion robust, apically straight and evenly narrowed to pointed apex (Fig. 10D); in ventral aspect basally narrow, medially and apically very broad and robust, lateral margins broadly curved, apically abruptly convergent to broadly angulate apex (Fig. 10E). Lateral lobe broad basally, elongate, moderately narrow apically, ventral margin sublinear, dorsal margin sinuate, margins convergent to narrowly rounded apex, apicodorsal margin with series of setae (Fig. 10F).

*Female genitalia*. Not examined.

*Sexual dimorphism*. Male pro- and mesotarsi I–III more broadly expanded than female and ventrally with several large adhesive setae.

*Variation*. Few specimens were examined and no significant variation was discovered.

#### Etymology.

This species is named *bimaculatus*, Latin for “two spots,” for the two maculae present apically on the elytra.

#### Distribution.

This species is known only from Cerro de la Neblina, Amazonas, Venezuela (Fig. 44).

#### Habitat.

*Hydrodessus bimaculatus* has been collected from “rocks in rapids” and “netted along margins” of the Rio Baria..

#### Specimens.

Holotype: ♂ in MIZA labeled, “VENEZUELA,T.F.Amaz. Cerro de la Neblina Basecamp, 140 m. 0°50'N, 66°10'W 28 January 1985/ seined from rocks in rapids of Rio Baria P.J. & P.M.Spangler, R.Faitoute,W.Steiner/ HYDRODESSUS CRAFTI [handwritten]/ HOLOTYPE *Hemibidessus bimaculatus* Miller, 2016 [red label with black line border].”

Paratype, 1 total. **Venezuela**; Amazonas, Cerro de la Neblina, basecamp, 140m, 0°50'N, 66°10'W, 20 Feb 1985, netted along margins of Rio Baria, P.J. and P.M. Spangler, R. Faitoute, W. Steiner (1, USNM).

### Hydrodessus brasiliensis

Taxon classificationAnimaliaColeopteraDytiscidae

(Guignot, 1957)

Figs 11 , 42

Brinckius brasiliensis Guignot, 1957: 40.
Hydrodessus brasiliensis , Young 1969: 2; 1970: 158; Spangler 1985: 88; Biström 1988: 37; Nilsson 2001: 236.

#### Type locality.

Brazil, Pará State, Cachimbo.

#### Diagnosis.

*Hydrodessus brasiliensis* is characterized by being concolorous dark red-brown (Fig. 11A). The lateral elytral carina is prominent, extending to about 1/2 length of elytron (Fig. 11B). The pronotum is about the same width as the greatest distance across the elytra (Fig. 11A). Ventrally, the prosternal process is broad but has distinctive laterally-directed lobes anteriorly and is constricted medially (Fig. 11C). The metaventral platform is not strongly constricted and the metaventrite carinae are moderately divergent posteriorly with the posterior apices ending near the anterior apices of the metacoxal lines (Fig. 11C). The male median lobe in lateral aspect is broadly triangular basally with the apical portion broadly curved, slender and apically slightly sinuate, slender and sharply pointed (Fig. 11D). The median lobe in ventral aspect is bilaterally symmatrical and nearly parallel-sided with the apex broadly rounded (Fig. 11E). The lateral lobe is relatively narrow, medially curved and has the apical portion gently tapered to broadly rounded apex (Fig. 11F). There is a setal margin extending around much of the apical half (Fig. 11F). The species is not particularly similar to others in the genus.

#### Description.

*Measurements*. TL = 3.0 mm, GW = 1.3 mm, PW = 1.2 mm, HW = 0.9 mm, EW = 0.5 mm, TL/GW = 2.3, HW/EW = 1.8. Body elongate, apically pointed, lateral outline moderately discontinuous between pronotum and elytron (Fig. 11A).

*Coloration* (Fig. 11A). Head, pronotum and elytra evenly dark red-brown, (Fig. 11A). Antennae, palps and legs red-brown. Venter dark red-brown throughout.

*Sculpture and structure*. Head broad, anterior clypeal margin broadly curved; surface shiny, covered with dense, fine punctures; eyes moderately large. Pronotum slightly cordate, widest anterior of middle (Fig. 11A); lateral bead very fine and continuous; surface with fine punctation, mediolaterally with punctures somewhat connected and irregular rugose. Elytra elongate, apex pointed, slightly constricted subapically (Fig. 11A); lateral carina sharp and distinct, extending to about half elytral length (Fig. 11B); surface covered with fine, relatively dense punctation. Prosternum medially tectiform and setose; prosternal process broad, distinctly constricted medially with prominent lateral lobes anteriorly, distinctly impressed longitudinally, apex broadly truncate (Fig. 11C). Metaventrite elongate, slightly impressed longitudinally, apex narrowly rounded; metasternal carinae distinctive, moderately closely approximated anteriorly, distinctly divergent posteriorly (Fig. 11C); other surfaces covered with fine, dense punctation. Legs covered with fine punctures on most surfaces; metatibia with distinctive brush of dense, elongate setae on postero-apical surface; pro- and mesotibiae moderately broad; metatrochanter distinctly offset, apically rounded; metacoxa evenly covered with fine punctures; metacoxal lines relatively closely approximated, subparallel (Fig. 11C). Abdomen evenly covered with fine punctures; apex of VI medially broadly pointed.

*Male genitalia*. Median lobe bilaterally symmetrical, in lateral aspect with basal portion broad, medially broadly curved and slender, apically slender and narrowed to slightly but distinctly sinuate, sharply pointed apex (Fig. 11D); in ventral aspect moderately broad, lateral margins subparallel to rounded apex (Fig. 11E). Lateral lobe moderately broad basally, apically narrow with margins subparallel to narrowly rounded apex, apical margins with distinctive series of setae (Fig. 11F).

*Female genitalia*. Not examined.

*Sexual dimorphism*. Only the male holotype was examined.

*Variation.* Only the male holotype was examined.

#### Distribution.

*Hydrodessus brasiliensis* is known only from Cochimbo, Para, central Brazil (Fig. 42).

#### Habitat.

Nothing is known of the habitat of the species.

#### Discussion.

Only the male holotype specimen was examined of this species.

#### Specimens.

The holotype male specimen is in MZSP labeled, “Type [red label with black line border]/ ♂/Brasilien, Para Cochimbo X.1955 Pereira [black line border]/ 31921/ F. Guignot det., 1956 Brinckius brasiliensis sp. n. Type ♂ [handwritten].”

### Hydrodessus brevis sp. n.

Taxon classificationAnimaliaColeopteraDytiscidae

http://zoobank.org/6FB0D768-395B-4B76-B580-E6CF8A9813CF

Figs 12 , 44

#### Type locality.

Venezuela, Amazonas, Cerro de la Neblina, basecamp, 0.833°N, 66.167°W.

#### Diagnosis.

This species has the lateral elytral carina relatively short, present just at the humeral angle, and the body overall approximately concolorous dark red (Fig. 12A). *Hydrodessus brevis* is similar to *Hydrodessus palus* in shape, and other structures, but that species is pale yellow, a bit smaller (TL = 2.0 mm) with *Hydrodessus brevis* larger (TL = 2.5 mm). The male genitalia differ, as well, with the median lobe of *Hydrodessus brevis* broader and apically not sinuate. *Hydrodessus brevis* is extremely similar to *Hydrodessus pereirai*, but that species is considerably larger (TL = 3.9 mm). The male genitalia of *Hydrodessus pereirai* are unknown so were not compared.

#### Description.

*Measurements*. TL = 2.6 mm, GW = 1.2 mm, PW = 1.0 mm, HW = 0.7 mm, EW = 0.5 mm, TL/GW = 2.2, HW/EW = 1.5. Body shape elongate, apically pointed, lateral margin distinctly discontinous between pronotum and elytron (Fig. 12A).

*Coloration* (Fig. 12A). Head and pronotum red. Elytra red, distinctly darker than head and pronotum, lighter apically, elytra immaculate (Fig. 12A). Antennae, palps and legs yellow to red-yellow. Venter red, lighter laterally on prothorax, elytral epipleuron and apex of abdomen.

*Sculpture and structure*. Head broad, anterior clypeal margin broadly rounded; surface shiny with fine, indistinct punctures; eyes small. Pronotum slightly cordate, broadest slightly anterior of middle (Fig. 12A); lateral bead fine; surface shiny and covered with very fine punctures. Elytra elongate, apically pointed, lateral margins slightly and broadly curved (Fig. 12A); lateral carina distinct but short, about 1/5 length of elytra (Fig. 12B); surface covered with fine punctures. Prosternum medially carinate and setose; prosternal process very broad, broadest at anterior lateral lobes, lateral carinae evenly convergent to broadly truncate apex, deeply excavated medially (Fig. 12C). Metaventrite with anterior process moderately broad, apically truncate, medially somewhat excavated; metasternal carinae distinct, curved medially and diverging posteriorly, terminating near anterior ends of metacoxal lines (Fig. 12C); surfaces covered with fine punctures. Legs with most surfaces covered with fine punctation throughout; pro- and mesotibiae slender; metatibia with posteroapical brush of setae distinctive; metatrochanter distinctly offset, apically rounded; metacoxa covered with fine punctures; metacoxal lines subparallel, moderately closely approximated, slightly convergent anteriorly (Fig. 12C). Abdomen covered with fine punctation; ventrite VI apically narrowly rounded.

*Male genitalia*. Median lobe bilaterally symmetrical, in lateral aspect broadly curved, curvature more pronounced basally, basal region broad, rounded, apical portion slender, slightly expanded submedially along ventral margin, apex slender and narrowly rounded (Fig. 12D); in ventral aspect basally narrow, lateral margins evenly curved and apically evenly convergent to angulate apex (Fig. 12E). Lateral lobe slender, moderately broad basally, elongate triangular apically with lateral margins evenly convergent to rounded apex (Fig. 12F).

*Female genitalia*. Not examined.

*Sexual dimorphism*. Male pro- and mesotarsi I–III slightly more broadly expanded than female and ventrally with several large adhesive setae.

*Variation.* No significant variation was observed in the few specimens examined.

#### Etymology.

This species is named *brevis*, Latin for “short,” for the relatively short lateral elytral carina in specimens.

#### Distribution.

*Hydrodessus brevis* is known only from Cerro de la Neblina, Amazonas, Venezuela (Fig. 44).

#### Habitat.

The two specimens in the type series were collected from leaf pack from among rocks in a small rainforest stream.

#### Specimens.

The holotype male is in MIZA labeled, “VENEZUELA,T.F.Amaz. Cerro de la Neblina Basecamp. 140 m. 0°50'N, 66°10'W 18 February 1985/ From leaf pack among rocks in small stream in rainforest P.J. & P.M.Spangler, R.Faitoute, W.Steiner/ HOLOTYPE *Hydrodessus brevis* Miller, 2016 [red line with black line border].”

Paratypes, 4 total. **Venezuela**, Amazonas, Cerro de la Neblina, basecamp, 0.833°N, 66.167°W, 7 Feb 1985, leaf pack among rocks in small stream in rainforest P.J. Spangler, P.M. Spangler, R. Faitoute, W. Steiner (4, MIZA, USNM).

### Hydrodessus concolorans sp. n.

Taxon classificationAnimaliaColeopteraDytiscidae

http://zoobank.org/45E30202-D75A-4388-A08E-048A4450C50E

Figs 13 , 36 , 42

#### Type locality.

Venezuela, Amazonas, Cerro de la Neblina, basecamp, 0.833°N, 66.167°W.

#### Diagnosis.

This species is dorsally shiny and concolorous dark red (Fig. 13A). The lateral elytral carina extends about 1/3 length of elytron (Fig. 13B), the prosternal process is very broad and excavated with the lateral margins rounded (Fig. 13C). The metaventrite carinae are not closely approximated anteriorly (Fig. 13C). Specimens are similar to *Hydrodessus continuus*, but the metacoxal lines in *Hydrodessus concolorans* meet the metaventrite/metacoxal suture at a prominent angle (Fig. 13C). The pronotum width is relatively narrowed compared with the greatest width across the elytra (Fig. 13A, EW/PW > 1.3). The male median lobe in lateral aspect is triangular basally, but very slender and evenly curved through apical portion (Fig. 13D). The apex is slender and pointed (Fig. 13D). The median lobe in ventral aspect is relatively parallel-sided to narrowed and narrowly rounded apex (Fig. 13E). The lateral lobe is basally moderately broad with apical half elongate triangular and the apex narrowly rounded (Fig. 13F).

#### Description.

*Measurements*. TL = 2.6–3.1 mm, GW = 1.3–1.5 mm, PW = 1.0–1.2 mm, HW = 0.8–0.9 mm, EW = 0.5 mm, TL/GW = 2.0–2.1, HW/EW = 1.6. Body elongate, apically pointed, lateral outline distinctly discontinous between pronotum and elytron (Fig. 13A).

*Coloration* (Fig. 13A). Head and pronotum red. Elytron red, apically red to red-yellow; some specimens with pale subapical macula. Antennae and palps yellow-red. Legs yellow to yellow-red. Ventral surfaces yellow-red to yellow-brown, lighter on elytral epipleuron and abdominal apex.

*Sculpture and structure*. Head broad, anterior clypeal margin subtruncate; surface shiny and microreticulate with few scattered, fine punctures; eyes large. Pronotum slightly cordate, widest near middle (Fig. 13A); lateral bead fine; surface shiny with scattered punctures. Elytra elongate, apically pointed (Fig. 13A); lateral carina distinct, extending about 1/3 length of elytron (Fig. 13B); surface indistinctly microretriculate, with fine punctation on surface and two indistinct longitudinal impressed lines (Fig. 13C). Prosternum medially distinctly carinate and setose; prosternal proces large and quadrate, broad, lateral carinae subparallel, medially deeply excavated, apex broadly truncate (Fig. 13C). Metaventrite with anterior process broad, anteriorly truncate, slightly constricted anteapically, distinctly excavated medially; metasternal carinae very well developed, evenly diverging posteriorly, broadly expanded posteriorly, terminating near anterior ends of metacoxal lines (Fig. 13C); other surfaces finely punctate. Legs with most surfaces covered with fine punctation; pro- and mesotarsi moderately broad; metatibia with posteroapical brush of setae; metatrochanter not strongly offset, apex narrowly rounded; metacoxa covered with fine punctures; metacoxal lines conspicuous, broadly separated, divergent anteriorly (Fig. 13C). Abdomen covered with fine punctation; apex of abdominal ventrite VI pointed with small, medial, spinous lobe.

*Male genitalia*. Median lobe bilaterally symmetrical, in lateral aspect evenly and moderately broadly curved throughout, basal portion small, apical portion long and slender to pointed apex (Fig. 13D); in ventral aspect narrow, lateral margins subparallel, apically evenly convergent to narrowly rounded apex (Fig. 13E). Lateral lobe elongate triangular, basally moderately broad, apically with lateral margins evenly convergent to pointed apex (Fig. 13F).

*Female genitalia*. Gonocoxosternite broadly triangular, medial margin slightly convex, apicolateral margin slightly concave, apex broadly rounded, anterior portion broad, anteriorly very broadly rounded (Fig. 36). Gonocoxa with apical portion elongate triangular, medial margin medially angled, apex broadly rounded, anterior apodeme elongate, as long as apical portion (Fig. 36). Bursa short, broad; spermathecal duct very long and slender, expanded near receptacle which is semispherical; spermatheca bulbous, with distinctive spermathecal spine; fertilization duct very long and slender (Fig. 36).

*Sexual dimorphism*. Male pro- and mesotarsi I–III more broadly expanded than female and ventrally with several large adhesive setae.

*Variation*. Some specimens have pale subapical maculae on the elytra, especially teneral specimens, but most specimens do not have these maculae distinctly visible.

#### Etymology.

This species is named *concolorans*, Latin for “concolorous,” for the generally even coloration of specimens.

#### Distribution.

This species is known only from the type locality area, Cerro de la Neblina, Amazonas, Venezuela (Fig. 42).

#### Habitat.

Specimens have been collected from along the margins and from rocks in rapids in a forest river and from a muddy oxbow pond in a rainforest clearing.

#### Specimens.

The holotype male in MIZA is labeled, “VENEZUELA:T.F.Amaz. Cerro de la Neblina Basecamp. 140 m. 0°50'N, 66°10'W 27 January 1985/ Netted along margins of Rio Baria P.J. & P.M.Spangler, R.Faitoute,W.Steiner/ HOLOTYPE Hydrodessus concolorans Miller, 2016 [red label with black line border].”

Paratypes, 120 total. **Venezuela**, Amazonas, Cerro de la Neblina, basecamp, 0.833°N, 66.167°W, 27 Jan 1985, netted along margins of Rio Baria, 140m, P.J. Spangler, P.M. Spangler, R. Faitoute, W. Steiner (22, USNM, MSBA, MIZA, KUNHM); same except 21 Feb 1985, muddy oxbow pond, rainforest clearing, 140m, P.J. Spangler, P.M. Spangler, R. Faitoute, W. Steiner (11, USNM, MSBA, MIZA, KUNHM); same except 28 Jan 1985, seined from rocks in rapids of Rio Baria, 140m, P.J. Spangler, P.M. Spangler, R. Faitoute, W. Steiner (88, USNM, MSBA, MIZA, KUNHM).

### Hydrodessus continuus

Taxon classificationAnimaliaColeopteraDytiscidae

sp .n.

http://zoobank.org/96195017-8196-4428-97B1-2EAB256811D3

Figs 14 , 44

#### Type locality.

Venezuela, Amazonas, Cerro de la Neblina, 1km SE basecamp, 0.833°N, 66.167°W.

#### Diagnosis.

This species differs from others by being dorsally nearly concolorous but with indistinct paler regions subapically and apically (Fig. 14A), having the lateral elytral carina about 1/3 length of elytron (Fig. 14B), having the metaventrite platform (the area between the metaventrite carinae) not strongly constricted (Fig. 14C), and having the metacoxal lines approximately continuously curved with the suture between the metaventrite and metacoxae (Fig. 14C). Specimens are similar to *Hydrodessus concolorans* and *Hydrodessus octospilus* in general shape and coloration, but they have the metacoxal lines intersecting the metaventrite/metacoxal suture at an angle. The male median lobe is very broadly curved with an elongate triangular basal portion and the apical portion very slender, evenly curved, and apically sharply pointed (Fig. 14D). The median lobe in ventral aspect is broad with subparallel margins in the basal half (Fig. 14E). In the apical half it is strongly constricted to an elongate, slender apically narrowly rounded apex (Fig. 14E). The lateral lobe is very broad, evenly curved and tapered to the rounded apex (Fig. 14F). There is a dense series of setae in a cluster subapically on the dorsal margin (Fig. 14F).

#### Description.

*Measurements*. TL = 2.9–3.0 mm, GW = 1.4 mm, PW = 1.1 mm, HW = 0.8 mm, EW = 0.5 mm, TL/GW = 2.1–2.2, HW/EW = 1.7. Body elongate, apically pointed, lateral outline distinctly discontinous between pronotum and elytron (Fig. 14A).

*Coloration* (Fig. 14A). Head and pronotum red. Elytra red with large, indistinct pale yellow region subapically, apex diffusely yellow (Fig. 14A). Antennae and palps red-yellow. Legs yellow, metacoxa red. Venter red, lighter red-yellow on head, prothorax, elytral epipleuron and apex of abdomen.

*Sculpture and structure*. Head moderately broad, anterior clypeal margins broadly rounded; surface shiny, microreticulate with few sparse punctures; eyes large. Pronotum cordate, widest near anterior margin (Fig. 14A); lateral bead fine and distinct throughout length; surface shiny, covered with very fine, indistinct punctures. Elytra elongate, apically pointed (Fig. 14A); lateral carina distinctive but short, about 1/3 length of elytron (Fig. 14B); surface shiny with punctures very fine over entire surface, with two moderately impressed longitudinal lines on disc. Prosternum medially somewhat swollen, broadly rounded; prosternal process broad, widest at anterior angles, medially strongly excavated, especially apically, apex broadly truncate (Fig. 14C). Metaventrite with anterior process moderately broad, apically truncate, medially distinctly impressed; metasternal carinae distinctive across metasternum, slightly curved and moderately divergent posteriorly, ending near anterior ends of metacoxal lines (Fig. 14C); other surfaces finely punctate. Legs shiny, most surfaces with very fine, indistinct punctures; metatibia with posteroapical brush of setae distinctive; pro- and mesotibiae moderately broad; metatrochanter somewhat offset, apically somewhat rounded; metacoxa covered with fine punctures; metacoxal lines sinuate, anteriorly broadly divergent (Fig. 14C). Abdomen covered with fine punctures; VI apically narrowly rounded.

*Male genitalia*. Median lobe bilaterally symmetrical, in lateral aspect strongly curved medially, apical portion more linear, basal region large, transverse, apical region slender, apically sharply pointed (Fig. 14D); in ventral aspect broad in basal half, lateral margins slightly concave, medially constricted and apical half strongly convergent to slender, apically pointed apex (Fig. 14E). Lateral lobe very broad basally, broadly curved to narrowly rounded apex, with dense brush of setae subapically along dorsal margin (Fig. 14F).

*Female genitalia*. Not examined.

*Sexual dimorphism*. Male pro- and mesotarsi I–III more broadly expanded than female and ventrally with several large adhesive setae.

*Variation.* Few specimens were examined, and no significant variation was discovered.

#### Etymology.

This species is named *continuus*, Latin for “continuous,” for the metacoxal lines which are approximately continuously curved with the suture between the metaventrite and metacoxa.

#### Distribution.

This species is known only from Cerro de la Neblina, Amazonas, Venezuela.

#### Habitat.

One specimen was collected from the margin of a river and the other known specimens from a blacklight.

#### Specimens.

The holotype male is in MIZA labeled, “VENEZUELA,T.F.Amaz. Cerro de la Neblina 1 km SE Basecamp 0°50'N, 66°10'W 140 m., 22 Feb.1985/ Netted along margins of Rio Baria P.J. & P.M.Spangler, R.Faitoute,W.Steiner/ HOLOTYPE *Hydrodessus continuus* Miller, 2016 [red label with black line border].”

Paratype, 1 total. **Venezuela**, Amazonas, Cerro de la Neblina, basecamp, 0.833°N, 66.167°W, 6 Feb 1985, blacklight on bank of Rio Baria, 140m, W.E. Steiner (1, USNM).

### Hydrodessus disjunctus sp. n.

Taxon classificationAnimaliaColeopteraDytiscidae

http://zoobank.org/43923C82-FC77-4298-A853-1B0D1237E0A5

Figs 15 , 42

#### Type locality.

Suriname, Sipaliwini District, Tafelberg Summit near Augustus Creek Camp, 3.933°N, 56.183°W.

#### Diagnosis.

This species is moderately elongate and dorsally and ventrally nearly concolorous red, without maculae on the elytra (Fig. 15A). The elytral apices are not dehiscent (Fig. 15A). The lateral elytral carinae extend about 1/4 length of elytron (Fig. 15B). The prosternal process is very broad and apically broadly truncate, broadly excavated medially, and slightly broader anteriorly (Fig. 15C). The metaventrite carinae are prominent, not medially constricted and posteriorly somewhat divergent, but the posterior apices are located distinctly mediad of the anterior apices of the metacoxal lines (Fig. 15C). The male median lobe in lateral aspect is relatively small basally with the apical portion slender, broadly and evenly curved (Fig. 15D). The apical portion is slightly constricted subapically, slender and pointed apically (Fig. 15D). The median lobe in ventral aspect is bilaterally symmetrical and slightly broadly expanded medially with the apical portion evenly convergent to moderately broadly pointed apex (Fig. 15E). The lateral lobe is moderately broad and with the lateral margins subparallel to the obliquely truncate apex which is somewhat, but distinctly, emarginate subapically (Fig. 15F). This species is most similar to *Hydrodessus bimaculatus* and *Hydrodessus biguttatus*. From *Hydrodessus biguttatus* it differs in the absence of dehiscent elytral apices and the shape of the male genitalia. From *Hydrodessus bimaculatus* this species differs in size (*Hydrodessus bimaculatus* are longer, TL > 3.5 mm) and the male genitalia are different.

#### Description.

*Measurements*. TL = 2.7–2.8 mm, GW = 1.3–1.4 mm, PW = 1.1–1.2 mm, HW = 0.8 mm, EW = 0.4–0.5 mm, TL/GW = 2.1, HW/EW = 1.7. Body elongate, apically pointed, lateral outline distinctly discontinous between pronotum and elytron (Fig. 15A).

*Coloration* (Fig. 15A). Head and pronotum red to red-orange. Elytra evenly red with apex diffusely yellow (Fig. 15A). Antennae and palps red-yellow, antennomeres I–III darker red. Legs yellow-brown, metacoxa red-brown. Venter red, lighter red-yellow on head, prothorax, elytral epipleuron and apex of abdomen.

*Sculpture and structure*. Head broad, anterior clypeal margin broadly rounded, with fine marginal, flattened bead; surface shiny, microreticulate with few sparse punctures; eyes large. Pronotum subcordate, widest near anterior margin (Fig. 15A); lateral bead fine and distinct throughout length; surface shiny, covered with very fine, indistinct punctures, laterally somewhat rugose. Elytra elongate, apically pointed (Fig. 15A); lateral carina distinctive but short, extending about ¼ length of elytron (Fig. 15B); surface shiny with punctures very fine over entire surface, with one moderately impressed longitudinal line on disc. Prosternum medially somewhat swollen, rounded; prosternal process very broad, subquadrate, widest at anterior angles, deeply excavated medially, lateral carinate margins slightly convergent posteriorly, apex broad, broadly truncate (Fig. 15C). Metaventrite with anterior process broad, apically broadly rounded, medially flattened; metasternal carinae low and rounded but distinct, extending nearly across metasternum, lines constricted anteriorly, somewhat curved and slightly divergent posteriorly, terminating distinctly mediad of anterior ends of metacoxal lines (Fig. 15C); surfaces covered with fine punctures. Legs shiny, most surfaces with very fine, indistinct punctures; metatibia with posteroapical brush of setae distinctive; pro- and mesotibiae narrow; metatrochanter offset, apically rounded; metacoxa covered with fine punctures; metacoxal lines low and rounded, broadly separated, divergent anteriorly (Fig. 15C). Abdomen covered with fine punctures; VI apically broadly pointed.

*Male genitalia*. Median lobe bilaterally symmetrical, in lateral aspect narrow basally, slender and evenly and broadly curved, subapically slightly narrowed and curved to sharply pointed apex (Fig. 15D); in ventral aspect moderately narrow, lateral margins broadly curved, apically narrowly rounded (Fig. 15E). Lateral lobe broad basally, apically broad and straight, apically obliquely bilobed on dorsal margin, apex with dense fringe of setae (Fig. 15F).

*Female genitalia*. Not examined.

*Sexual dimorphism*. Male pro- and mesotarsi I–III more broadly expanded than female and ventrally with several large adhesive setae.

*Variation.* Only two specimens were examined, and no significant variation was discovered.

#### Etymology.

This species is named *disjunctus*, Latin for “separated,” for the distinctive distance separation between the posterior apices of the metaventrite carinae and the anterior apices of the metacoxal lines.

#### Distribution.

This species is known only from the type specimens from the Tafelberg in Sipaliwini District, Suriname (Fig. 42).

#### Habitat.

Specimens were collected from “forested creek margins.”

#### Specimens.

The holotype male is in NZCS labeled, “SURINAME: Sipaliwini District N3°55.600', W56°11.300', 600m CSNR: Tafelberg Summit nr Augustus Creek Camp forested creek margins leg. Short & Bloom: 22.viii.2013 SR13-082202B/ SEMC1080468 KUNHM-ENT [barcode label]/ HOLOTYPE *Hydrodessus disjunctus* Miller, 2016 [red label with black line border].”

Paratype, 1 total. **Suriname**, Sipaliwini District, Tafelberg Summit near Augustus Creek Camp, 3.933°N, 56.183°W, 22 Aug 2013, forested creek margins, Short and Bloom (1, KUNHM, SEMC1080471).

### Hydrodessus fasciatus sp. n.

Taxon classificationAnimaliaColeopteraDytiscidae

http://zoobank.org/B8868F1C-B780-43F1-B609-2E909A22238A

Figs 16 , 45

#### Type locality.

Brazil, Rio Gurupi, 12–15km E Caninde-Igarape Coraci.

#### Diagnosis.

This species is dorsally dark brown with distinctive, irregular fasciae on the elytra (Fig. 16A). The fasciate are somewhat linear-sided making the pale regions subrectangular (Fig. 16A). The lateral elytral carina is absent (Fig. 16B). The prosternal process is elongate and somewhat slender with the lateral margins subparallel (Fig. 16C). The anterior metaventrite process is moderately slender and medially impressed (Fig. 16C). The metaventrite carinae are distinct only anteriorly (Fig. 16C). The male median lobe in lateral aspect has a small basal region (Fig. 16D). The apical portion is evenly curved along the dorsal margin, but thickened subbasally and subapically along the ventral margin (Fig. 16D). The apex is elongate, slender and narrowly rounded apically (Fig. 16D). In ventral aspect the median lobe is slender, bilaterally asymmetrical and apically narrowly and obliquely rounded (Fig. 16E). The lateral lobe is moderately slender and broadly curved to a rounded apex (Fig. 16F). The species is perhaps most similar to *Hydrodessus siolii* in body shape and structure, but that species has a different color pattern and the male genitalia are distinctive in each species.

#### Description.

*Measurements*. TL = 2.7–2.8 mm, GW = 1.3 mm, PW = 1.0–1.1 mm, HW = 0.8–0.9 mm, EW = 0.4 mm, TL/GW = 2.1–2.2, HW/EW = 1.9–2.1. Body shape slender, elongate, apically pointed, lateral margins distinctly discontinuous between pronotum and elytron (Fig. 16A).

*Coloration* (Fig. 16A). Head and pronotum yellow-brown. Elytra fasciate, with longitudinal yellow maculae on red-brown background (Fig. 16A). Antennae and palpi yellow. Legs yellow brown. Ventral surfaces yello-brown, lighter on elytral epipleuron and apex of abdomen.

*Sculpture and structure*. Head moderately elongate, anterior clypeal margin broadly rounded; surface covered with few, sparse, fine punctures; eyes large. Pronotum subcordate, widest slightly anterior to middle; lateral bead fine; surface shiny with distinctive, moderately dense punctures. Elytra elongate, lateral margins subparallel in anterior 2/3 (Fig. 16A); lateral carina absent (Fig. 16B); surface similar to pronotum. Prosternum medially tectiform and setose; prosternal process elongate, slender, lateral margins subparallel, widest medially, longitudinally impressed, apex rounded (Fig. 16C). Metaventrite with anterior process short, slender, longitudinally impressed, apex narrowly rounded; metaventrite carina present mainly anteriorly along process, closely approximated, absent posteriorly (Fig. 16C); other surfaces sparsely punctate. Legs shiny, relatively impunctate; pro- and mesotibiae moderately broad; metatrochanter distinctly offset, apex rounded; metatibia with posteroapical brush of setae distinctive; metacoxa shiny and sparsely punctate; metacoxal lines broadly separated, anteriorly divergent (Fig. 16C). Abdomen shiny, sparsely punctate; ventrite VI apically somewhat narrowly rounded.

*Male genitalia*. Median lobe bilaterally slightly asymmetrical, in lateral aspect broadly curved, distinctly expanded in two places along ventral margin, submedially and subapically, apex narrowed to pointed apex (Fig. 16D); in ventral aspect narrow, lateral margins unevenly convergent to asymmetrical apex which is slightly curved to right (Fig. 16E). Lateral lobe moderately broad basally, elongate slender apically to rounded apex, with series of setae along apical margin (Fig. 16F).

*Female genitalia*. Not examined.

*Sexual dimorphism*. Male pro- and mesotarsi I–III only slightly more broadly expanded than female and ventrally with several large adhesive setae.

*Variation*. Very little variation was examined in the few specimens examined.

#### Etymology.

This species is named *fasciatus*, Latin for “striped,” for the fasciate color pattern on the elytra.

#### Distribution.

*Hydrodessus fasciatus* is known only from the type locality in Pará, Brazil (Fig. 45).

#### Habitat.

Nothing is known of the natural history of this species.

#### Specimens.

The holotype male is in FSCA labeled, “BRASIL:Para Rio Gurupi 12–15 km e. Caninde-Igarape Coraci xii.19.1965 #12 Borys Malkin/ *Hydrodessus fasciatus* Miller, 2016 [red label with black line border].”

Paratypes, 2 total. **Brazil**, Rio Gurupi 12–15 km E Caninde-Igarape Coraci, 19 Dec 1965, B. Malkin (2, FSCA).

### Hydrodessus imparilis sp. n.

Taxon classificationAnimaliaColeopteraDytiscidae

http://zoobank.org/B59FC24E-178C-49E1-ACAF-8B20769CA19E

Figs 17 , 42

#### Type locality.

Ecuador, Provincia de Napo, Limococha on Rio Napo, 0.737°S 78.111°W.

#### Diagnosis.

This species is dorsally largely red with the pronotum orange and the elytral apex, lateral margins, and a moderately well-defined macula at about 2/3 length of elytron (Fig. 17A). The lateral margin is more broadly orange near the humeral angle (Fig. 17A). Also, there are very weakly-defined longitudinal fasciae indistinctly present on the anterior half of the elytron (Fig. 17A). The prosternal process has well-developed lateral lobes anteriorly (Fig. 16C). The metaventrite carinae are together strongly constricted immediately posterad to the metaventral process and are strongly divergent posteriorly (Fig. 16C). The male median lobe is elongate triangular basally with a sharp bend at base of apical portion (Fig. 16D). The apical portion is slender and weakly curved to near apex which is very slender and distinctly sinuate with the apex sharply pointed (Fig. 16D). The median lobe in ventral aspect is subparallel but bilaterally asymmetrical with the apex obliquely truncate (Fig. 16E). The lateral lobe is broadly triangular with the apex obliquely truncate (Fig. 16F). There are two series of setae, apically and along the dorsal margin (Fig. 16F).

#### Description.

*Measurements*. TL = 2.9 mm, GW = 1.3 mm, PW = 1.1 mm, HW = 0.8 mm, EW = 0.5 mm, TL/GW = 2.2, HW/EW = 1.6. Body shape elongate, narrow, apically pointed, lateral margins slightly, evenly discontinuous between pronotum and elytron (Fig. 16A).

*Coloration* (Fig. 16A). Head yellow-red. Pronotum yellow. Elytra yellow-red, with diffuse, small yellow maculae anterolaterally and apicomedially and apex yellow (Fig. 16A). Antennae, palpi and legs yellow. Venter red-brown, lighter on epipleuron.

*Sculpture and structure*. Head broad, anterior clypeal margin broadly rounded; surface shiny with many fine punctures throughout; eyes small. Pronotum narrow, widest at posterior margins, lateral margins weakly curved (Fig. 16A); lateral bead very fine; surface medially similar to head, laterally shiny, irregularly rugulose. Elytra elongate, apically pointed, laterally very broadly curved (Fig. 16A); lateral carina distinctive, but very short, about 1/8 length of elytra (Fig. 16B); surface covereed with fine punctation. Prosternum medially slightly tectiform, setose; prosternal process moderately broad, widest at anterior lateral lobes, lateral margins slightly converging to rounded, thickened apex, with prominent lateral carinae and medial, longitudinally impressed area (Fig. 16C). Legs with surfaces covered with fine punctation; pro- and mesotibiae broad; metatibia with posteroapical brush of setae distinctive; metatrochanter slightly offset, apex slightly flattened and narrowly rounded; metacoxa covered with fine punctation; metacoxal lines moderately separated, evenly divergent anteriorly (Fig. 16C). Abdomen covered with fine punctures; ventrite VI apically rounded.

*Male genitalia*. Median lobe bilaterally asymmetrical, in lateral aspect with basal region elongate subtriangular, abruptly curved medially, slightly curved in apical half, gradually expanded along ventral margin, apically sinuate with apex abruptly narrowed and apex pointed (Fig. 16D); in ventral narrow basally, lateral margins broadly curved, left margin more strongly curved, apex obliquely truncate (Fig. 16E). Lateral lobe broad, ventral margin broadly curved, dorsal margin slightly curved, apically narrowed with apex obliquely subtruncate, apex with series of setae and dorsal margin with medial series of setae (Fig. 16F).

*Female genitalia*. Not examined.

*Sexual dimorphism*. Female not examined.

*Variation*. Only a single specimens of this species was examined.

#### Etymology.

This species is named *imparilis*, Latin for “unequal,” for the the bilaterally asymmetrical male median lobe.

#### Distribution.

This species is known only from the type locality in Provincia de Napo, Ecuador (Fig. 42).

#### Habitat.

The single known specimen was collected at a black light.

#### Specimens.

The male holotype is in FSCA labeled, “ECUADOR Napo Prov. Limococha On Rio Nap BLT 10.xi.1974 BADrummond III/ *Hydrodessus imparilis* Miller, 2016 [red label with black line border].”

### Hydrodessus jethoeae

Taxon classificationAnimaliaColeopteraDytiscidae

Makhan, 1994: 119

Figs 18 , 42

Hydrodessus jethoeae Makhan, 1994: 119; Nilsson 2001: 236.

#### Type locality.

Surinam, District Brokopondo, Brownsweg.

#### Diagnosis.

*Hydrodessus jethoeae* is not particularly similar to any other species. Specimens are elongate and posteriorly attenuate (Fig. 18A). The dorsal surface is vaguely fasciate with variegations of yellow, brown and dark brown (Fig. 18A). The lateral elytral carina is elongate, extending about 1/2 length of the elytron (Fig. 18B). The prosternal process has distinctive lateral lobes anteriorly and is posteriorly abruptly narrowed (Fig. 18C). The metaventrite carinae are distinctive, extending across the metaventrite (Fig. 18C). They are slightly constricted anteriorly and moderately divergent posteriorly (Fig. 18C).

#### Description.

*Measurements*. TL = 2.9–3.0 mm, GW = 1.5 mm, PW = 1.4 mm, HW = 1.2–1.3 mm, EW = 0.9 mm, TL/GW = 2.0, HW/EW = 1.7. Body elongate, apically attenuated, very narrowly rounded, lateral margin discontinuous between pronotum and elytron (Fig. 18A).

*Coloration* (Fig. 18A). Head and pronotum yellow-orange. Elytra orange with diffuse pale areas anterolaterally, mediolaterally, subapically and in broad V-shape at apex, also with diffuse, dark brown areas laterally and around subapical pale region. Antennae, palpi and legs yellow. Venter yellow, dark on some sutures, especially basal abdominal sutures.

*Sculpture and structure*. Head broad, anterior clypeal margin broadly curved; surface shiny with many minute punctures; eyes large. Pronotum cordate, widest near anterior margin (Fig. 18A); lateral bead fine, somewhat obscured posteriorly; surface shiny, covered with fine punctures. Elytra elongate, apically strongly narrowed (Fig. 18A); lateral carina distinctive anteriorly, extending to about ½ length of elytron, but becoming lower and more rounded posteriorly (Fig. 18B); surface shiny, covered with fine punctures. Prosternum medially rounded and setose; prosternal process relatively slender, widest at anterior laterally expanded lobes, posteriorly abruptly narrowed, then margins subparallel to rounded apex, lateral margins strongly carinate, longitudinally strongly excavated (Fig. 18C). Metaventrite with anterior process slender, anteriorly rounded, longitudinally somewhat excavated (Fig. 18C); metasternal carinae closely approximated anteriorly, divergent but not broadly, posteriorly extending to posterior margin of metaventrite near anterior ends of metacoxal lines (Fig. 18C); surface covered with fine punctation. Legs with surfaces covered with fine punctation; pro- and mesotibiae moderately broad; metatrochanter strongly offset and apically rounded; metatibia with posteroapical brush of setae; metacoxa covered with fine punctation; metacoxal lines approximated, parallel (Fig. 18C). Abdominal ventrites covered with fine punctation; VI apically broadly rounded.

*Male genitalia*. Median lobe of type broken, lateral lobes absent. Median lobe in lateral aspect slender, curved basally, slightly curved through apical portion, apex slender and slightly recurved and deflexed, apically finely rounded (Fig. 18D); in ventral aspect slender, lateral margins slightly convergent, apex rounded (Fig. 18E).

*Female genitalia*. Not examined.

*Sexual dimorphism*. None examined.

*Variation*. Among the three specimens examined there is some minor variation in extent and pattern of coloration on the elytron.

#### Distribution.

In addition to the type locality in Brokopondo District, Suriname, this species is known from two sites, one in Bolivar State, Venezuela and another in Sipaliwini District, Suriname (Fig. 42).

#### Habitat.

*Hydrodessus jethoeae* has been collected from a river margin and at a UV light.

#### Discussion.

The type specimen had been dissected for examination in this study, and the base of the male median lobe and the lateral lobes were damaged and could not be illustrated. Other than the male type specimen, only two female specimens are known for this species, they are very similar to each other and distinct from all other species. They were also collected quite some distance from each other. Despite the lack of knowledge of males, it seems likely that future association of specimens with this species will not be problematic.

#### Specimens.

Holotype: ♂ in RMNH labeled, “Suriname District Brokopondo Brownsweg 7.8.1984 leg. D.Makhan/ Hydrodessus jethoeae det. D. Makhan 1994/ Holotype [red label].”

**Suriname**; Sipaliwini District, Camp 1, on Juari River, 2.175°N, 56.788°W, 19 Aug 2010, uv light, Short and Miller (1 female, KUNHM, SEMC0915510). **Venezuela**; Bolivar State Rio, Caripito, nr. Rio Orinoco, river margin, 6.58694°N; 67.02912°W 12.i.2009, Short & Miller VZ09-0112-02A/ [barcode label] (1 female, KUNHM, SEMC0854749).

### Hydrodessus keithi sp. n.

Taxon classificationAnimaliaColeopteraDytiscidae

http://zoobank.org/65C18926-114A-4DD1-9B99-CBE25C1030FC

Figs 1 , 5 , 19 , 37 , 50

#### Type locality.

Ecuador, Pastaza, Provinica Tzapino, 32km NE Tigueno, 1.183°N, 77.233°W.

#### Diagnosis.

*Hydrodessus keithi* has very characteristic coloration with the pronotum redi with testaceous margins and the elytra dark testaceous with distinctive maculae (Fig. 19A). There is a large subrectangular yellow macula at the humeral angle and a large subtriangular yellow macula at about 3/4 length of the elytron (Fig. 19A). The ventral surfaces are black. The lateral elytral carina is short and present only at the humeral angle (Fig. 19B). The prosternal process is relatively slender with moderately well-developed lateral lobes anteriorly (Fig. 19C). The metaventrite carinae are distinctive and strongly divergent posteriorly (Fig. 19C). The male median lobe in lateral aspect is slender and broadly curved with the apex subapically constricted on the ventral margin and apically sharply pointed (Fig. 19D). In ventral aspect, the apex is bilaterally symmetrical, broadly expanded and broadly rounded (Fig. 19E). The lateral lobe is large, broad and broadly sinuate with the apex broadly rounded (Fig. 19F). Males and females are dimorphic with the female apicolateral margin of the elytron distinctly flanged (Figs 5B, 19A1).

#### Description.

*Measurements*. TL = 2.6–2.9 mm, GW = 1.2–1.3 mm, PW = 1.0–1.1 mm, HW = 0.8 mm, EW = 0.5 mm, TL/GW = 2.1–2.2, HW/EW = 1.6. Body elongate, lateral margin conspicuously discontinuous between pronotum and elytron (Fig. 19A).

*Coloration* (Fig. 19A). Head yellow to yellow-brown, darker anterolateraly. Pronotum medially broadly yellow, laterally and posteriorly dark red. Elytron medially with broad, longitudinal black region subtending suture, medially with black or red-black region connecting to lateral margin of elytron, otherwise yellow, elytral coloration appearing as four, large, yellow maculae. Antennae and palpi yellow. Legs yellow except coxae, including metacoxae, black. Venter black except abdominal ventrites V and VI lighter, red-yellow and elytral epipleuron lighter yellowish apically.

*Sculpture and structure*. Head apically broadly subtrunctate, clypeus somewhat swollen laterally near eyes; surface covered with fine punctures; eyes large, conspicuous. Pronotum cordate, broadest near anterior margin (Fig. 19A), lateral bead slender; surface covered with fine punctures, somewhat rugulose anterolateraly. Elytra together elongate, apically slightly pointed (Fig. 19A); lateral carina inconspicuous, rounded, extending about ¼ length of elytron (Fig. 19B); elytral surface evenly covered with fine punctures. Prosternum medially carinate, with fine, long setae on each side of carina; prosternal process broad, lateral margins somewhat sinuate, broadest anteriorly with prominent lateral lobes, apex trunctate, longitudinally excavated (Fig. 19C). Metaventrite with anterior process prominent, parallel-sided, long; metasternal carinae inconspicuous, low, represented posteriorly by impunctate line, extending to near anterior ends of metacoxal lines (Fig. 19C); other surfaces covered with fine punctures. Legs with surfaces covered with fine punctures; metatibia with distinctive brush of dense, elongate setae on postero-apical surface; pro- and mesotibiae conspicuously broad; metatrochanter moderately offset, apex angulate; metacoxa evenly covered with fine punctures; metacoxal lines moderately broadly separated, subparallel and anteriorly slightly divergent (Fig. 19C). Abdomen evenly covered with fine punctures.

*Male genitalia*. Median lobe bilaterally symmetrical, in lateral aspect broadly curved, with basal portion short and subtriangular, apical portion elongate slender and broadly curved, apically with ventral margin broadly sinuate, subapically expanded, and with apex slender and pointed (Fig. 19D); in ventral aspect slender, medially constricted, apically expanded and apex broadly rounded (Fig. 19E). Lateral lobe broadly sinuate, broad basally, apical portion more slender and evenly curved ventrad, apex narrowly rounded and with a small cluster of setae (Fig. 19F).

*Female genitalia*. Gonocoxosternite triangular, medial margin straight, apical portion small (Fig. 37). Gonocoxa with apical portion slender, elongate triangular, anterior apodeme short (Fig. 37). Bursa short and broad; spermathecal duct slender, moderately short; receptacle semispherical; spermatheca undifferentiated from fertilization duct, without spermathecal spine, this combined structure extremely long and coiled, tapering to apex of fertilization duct (Fig. 37).

*Sexual dimorphism*. Male pro- and mesotarsi I–III more broadly expanded than female and ventrally with several large adhesive setae; female with elytron prominently expanded and lobate subapically (Figs 5B, 19A2), male evenly curved (Figs 5A, 19A1); male abdominal seternite VI evenly rounded across surface, apex with minute pointed lobe apically, female with prominent lateral depression on each side of VI.

*Variation*. Specimens are somewhat variable in coloration with some relatively lighter and others relatively darker.

#### Etymology.

This species is named *keithi* in honor of the author’s brother, Keith B. Miller.

#### Distribution.

*Hydrodessus keithi* has been found in Ecuador, Colombia and central Brazil (Fig. 50).

#### Habitat.

This species has been collected from blacklight traps. Nothing else is known about their habitat.

#### Specimens.

The holotype is in USNM labeled, “ECUADOR,Past. Prov.,Tzapino, 22May76ele.400m Jeffrey Cohen blacklight trap/ 1°11'S–77°14'W 32KmNE Tigueno/ ECUADOR-PEACE CORPS. SMITHSONIAN INSTITUTE AQUATIC INSECT SURVEY/ HOLOTYPE *Hydrodessus keithi* Miller, 2016 [red label with black line border].”

Paratypes, 24 total. **Brazil**, Para, Rio Gurupi, 12–15km E Caninde Igarape Coraci, 19 Dec 1965, B. Malkin (1, FSCA). **Colombia**, Meta, Villavicencio, National University Biological Station, 4.15°N, 73.633°W, 8 Jan 1973, blacklight trap, C.R. Gilbert (1, USNM). **Ecuador**, Napo, Limocha on Rio Napo, 0.737°S 78.111°W, 10 Nov 1974, BLT, B.A. Drummond (4, FSCA); Provincia Tzapino, Pastaza, 32km NE Tigueno, 1.183°N, 77.233°W, 22 May 1976, blacklight trap, Ecuador Peace Corps Smithsonian Institute Aquatic Insect Survey, 400m, J. Cohen (18, USNM).

### Hydrodessus kurti sp. n.

Taxon classificationAnimaliaColeopteraDytiscidae

http://zoobank.org/3BDC3EC9-D90F-4A23-8A4F-475AE8B45E53

Figs 20 , 47

#### Type locality.

Suriname, Sipaliwini District, Camp 1, Upper Palumeu, 2.477°N, 55.629°W.

#### Diagnosis.

This is a red species with the head and pronotum often somewhat lighter red and with moderately well-defined pale maculae on the elytra (Fig. 20A). There is one macula subapically that is triangular and a narrow macula at the apex (Fig. 20A). The carinae on the metaventrite are broadly divergent posteriorly with a prominent constriction immediately posterad of the metaventral process (Fig. 20C). This species is sexually dimorphic in body shape with the male apically broadly pointed (Fig. 20A1) and the female apically subtruncate to very broadly pointed (Fig. 20A2). The species is most similar to *Hydrodessus kylei* which has a similar sexual dimorphism. That species has the eyes conspicuously emarginate (best seen in dorsal aspect). The male genitalia are different, as well. The median lobe in *Hydrodessus kurti* is bilaterally symmetrical with the apex rounded in ventral aspect (Fig. 20E). In lateral aspect the median lobe is broadly curved with the apex very slender, sinuate and very sharply pointed (Fig. 20D). The median lobe in *Hydrodessus kylei* is bilaterally asymmetrical with the apex obliquely truncate in ventral aspect. In lateral aspect the median lobe is similarly broadly curved but apically somewhat more robust (Fig. 21D). The lateral lobe is considerably narrower in *Hydrodessus kurti* (Fig. 20F) than in *Hydrodessus kylie* (Fig. 21F).

#### Description.

*Measurements*. TL = 2.6–2.7 mm, GW = 1.3 mm, PW = 1.0 mm, HW = 0.7–0.8 mm, EW = 0.5 mm, TL/GW = 2.0–2.1, HW/EW = 1.5–1.6. Body moderately robust, apically pointed, lateral outline distinctly discontinous between pronotum and elytron (Fig. 20A).

*Coloration* (Fig. 20A). Head red. Pronotum red to orange-red laterally, medially and along anterior margin with large, diffuse dark red area. Elytra red with subapical pale macula, apex pale (Fig. 20A). Antennae and palps yellow-orange to yellow. Legs yellow-orange to yellow, metacoxa dark red. Venter dark red on most surfaces, lighter orange on, prothorax, elytral epipleuron and apex of abdomen.

*Sculpture and structure*. Head moderately broad, anterior clypeal margins broadly rounded; surface shiny, microreticulate with few sparse punctures; eyes large. Pronotum slightly cordate, widest near anterior margin (Fig. 20A); lateral bead very fine and distinct throughout length; surface shiny, covered with fine punctures. Elytra elongate, apically pointed (Fig. 20A); lateral carina distinctive but short, about 1/5 length of elytron (Fig. 20B); surface shiny with punctures fine over entire surface, with one moderately impressed longitudinal line medially on disc. Prosternum medially somewhat swollen, rounded; prosternal process moderately broad, widest at anterior angles, narrowed posteriorly with posterior portion with lateral carinate margins subparallel, medially longitudinally excavated, apex truncate (Fig. 20C). Metaventrite with anterior process moderately broad, apex rounded, medially slightly impressed with lateral margins broadly beaded; metasternal carinae distinctive across metasternum, though rounded and less distinctive posteriorly, slightly curved and distinctly divergent posteriorly, posterior terminus distinctly mediad of anterior ends of metacoxal lines (Fig. 20C); metaventrite covered with fine punctation. Legs shiny, most surfaces with very fine, indistinct punctures; metatibia with posteroapical brush of setae distinctive; pro- and mesotibiae broad; metatrochanter somewhat offset, apically narrowly rounded; metacoxa covered with fine punctures; metacoxal lines distinctive, broadly divergent anteriorly (Fig. 20C). Abdomen covered with fine punctures; VI apically laterally somewhat compressed with medially apex pointed.

*Male genitalia*. Median lobe bilaterally symmetrical, in lateral aspect moderately broad basally, medially strongly curved, slender, apically slender and apex slightly but distinctly sinuate, very slender and sharply pointed (Fig. 20D); in ventral aspect narrow basally, lateral margins slightly divergent apically to broadly rounded apex (Fig. 20E). Lateral lobe broad basally, apically broadly curved, apex straightened, broad, and apically broadly rounded, with distinct cluster of setae apically and along dorsal margin (Fig. 20F).

*Female genitalia*. Not examined.

*Sexual dimorphism*. Male pro- and mesotarsi I–III more broadly expanded than female and ventrally with several large adhesive setae. Females with posterolateral margins of elytra expanded laterally and broadly lobate (Fig. 20A2), males with elytral margins not lobed (Fig. 20A1). Female abdominal ventrite VI not as laterally compressed as in male, and less strongly pointed medially.

*Variation.* Specimens vary somewhat in depth of coloration. In particular, the medial darkened region of the pronotum is variable with some specimens having that area smaller and others larger.

#### Etymology.

This species is named *kurti* in honor of the author’s brother, Kurt B. Miller.

#### Distribution.

*Hydrodessus kurti* is known only from the type locality in southern Suriname (Fig. 47).

#### Habitat.

The type series was collected from a large, sandy creek.

#### Specimens.

The holotype male is in NZCS labeled, “SURINAME: Sipaliwini District N 2.47700°, W 55.62941°,275 m Camp 1, Upper Palumeu leg.A.Short; large sandy creek 14.iii.2012; SR12-0314-01A 2012 CI-RAP Survey/ SEMC1088337 KUNHM-ENT/ *Hydrodessus kurti* Miller, 2016 [red line with black line border].”

Paratypes, 6 total. **Suriname**, Sipaliwini District, Camp 1, Upper Palumeu, 2.477°N, 55.629°W, large sandy creek, 275m, A. Short (6, KUNHM, SEMC1088338, SEMC1088339, SEMC1088342, SEMC1088346, SEMC1088347, SEMC1088351).

### Hydrodessus kylei sp. n.

Taxon classificationAnimaliaColeopteraDytiscidae

http://zoobank.org/8465C02F-74C1-4F9D-8344-DF05443E0505

Figs 21 , 38 , 43

#### Type locality.

Venezuela, Amazonas, Cerro de la Neblina, basecamp, 0.833°N, 66.167°W.

#### Diagnosis.

*Hydrodessus kylei* is the only known *Hydrodessus* species with distinctly emarginate eyes (best seen in dorsal aspect) (Fig. 21A). This is a red species with the head and pronotum often somewhat lighter red and with moderately poorly-defined pale maculae on the elytra (Fig. 21A). There is one macula subapically that is triangular and a narrow lighter region apically (Fig. 21A). The carinae on the metaventrite are broadly divergent posteriorly with a prominent constriction immediately posterad of the metaventral process (Fig. 21C). This species is sexually dimorphic in body shape with the male apically broadly pointed (Fig. 21A1) and the female apically subtruncate to very broadly pointed (Fig. 21A2). The species is most similar to *Hydrodessus kurti* which has a similar sexual dimorphism but does not have emarginate eyes. The male genitalia are also different. The median lobe in *Hydrodessus kurti* is bilaterally symmetrical with the apex rounded in ventral aspect (Fig. 20E). In lateral aspect the median lobe is broadly curved with the apex very slender, sinuate and very sharply pointed (Fig. 20D). The median lobe in *Hydrodessus kylei* is bilaterally asymmetrical with the apex obliquely truncate in ventral aspect (Fig. 21E). In lateral aspect the median lobe is similarly broadly curved but apically somewhat more robust (Fig. 21D). The lateral lobe is considerably broader in *Hydrodessus kylei* (Fig. 21F) than in *Hydrodessus kurta* (Fig. 20F).

#### Description.

*Measurements*. TL = 2.7–2.8 mm, GW = 1.3 mm, PW = 0.9–1.1 mm, HW = 0.7 mm, EW = 0.3–0.5 mm, TL/GW = 2.1–2.2, HW/EW = 2.0–2.2. Body robust, broad, apically pointed, lateral outline slightly discontinuous between pronotum and elytron (Fig. 21A).

*Coloration* (Fig. 21A). Head dark red. Pronotum dark red, yellow-red anteriorly. Elytra dark red with diffuse, yellow-red macula subapically and with apex yellow-red (Fig. 21A). Antennae, palps and legs brown. Venter dark red-brown, lighter on elytral epipleuron and abdominal ventrites V-VI.

*Sculpture and structure*. Head broad, anterior margin broadly rounded; surface covered with microreticulation and very fine punctures; eyes large, laterally with distinctive concavity. Pronotum slightly cordate, widest near middle (Fig. 21A); surface shiny with fine microreticulation and irregular punctation with some larger and smaller. Elytra elongate, apex pointed; lateral carina distinctive, but short, only present near humeral angle (Fig. 21B); surface shiny with fine microreticulation and fine punctures. Prosternum medially carinate with long, fine setae; prosternal process moderately broad, widest at anterior lateral lobes, lateral carinae convergent posteriorly to rounded apex, medially longitudinally excavated (Fig. 21C). Metaventrite with anterior process moderately narrow, anteriorly truncate, constricted subapically, medially somewhat excavated; metasternal carinae distinctive, broadly divergent posteriorly, ending near anterior ends of metacoxal lines (Fig. 21C); surfaces covered with dense, fine punctation. Surfaces covered with fine punctation; pro- and mesotibiae not broad; metatibia with posteroapical brush of setae; metatrochanter only slightly offset, apically narrowly rounded. Metacoxa covered with dense, fine punctation; metacoxal lines prominent, broadly separate, distictly divergent anteriorly (Fig. 21C). Abdominal ventrites covered with dense, fine punctation; ventrite VI apically evenly rounded.

*Male genitalia*. Median lobe bilaterally asymmetrical, in lateral aspect very strongly curved, with base extremely large and triangular, apical portion strongly curved, dorsal margin somewhat expanded, apex slightly sinuate and narrowly rounded (Fig. 21D); in ventral aspect broad basally, basal half with lateral margins subparallel, left margin straight to near apex, right margin strongly constricted submedially, margin divergent medially, apex broadly expanded and strongly obliquely truncate (Fig. 21E). Lateral lobe extremely broad, ventral margin very strongly curved, dorsal margin concave, apex a narrowly rounded lobe directed posteriorly (Fig. 21F).

*Female genitalia*. Gonocoxosternite transversely broad, apex broadly angulate, anterior portion moderately large, subtriangular, anterior apex rounded (Fig. 38). Gonocoxa slender, apical portion slender and apically narrowly rounded, anterior apodeme longer than apical portion, slender (Fig. 38). Bursa extremely large, elongate and broad; spermathecal duct extremely long and slender, expanded near receptacle which is semispherical; spermatheca elongate and slender, with distinctive spermathecal spine; fertilization duct extremely long, slender, and coiled (Fig. 38).

*Sexual dimorphism*. Male pro- and mesotarsi I–III more broadly expanded than female and ventrally with several large adhesive setae. The female elytral apex is more broadly rounded, and subapically slightly lobed on each side than in male.

*Variation*. The subapical and apical pale areas are variably distinctive between specimens.

#### Etymology.

This species is named *kylei* in honor of the author’s brother, Kyle B. Miller.

#### Distribution.

This species is found in Amazonas, Venezuela and in southern Suriname (Fig. 43).

#### Habitat.

Specimens have been collected along the margins of a forest river, from a large, sandy creek, and at UV light.

#### Specimens.

The male holotype is in MIZA labeled, “VENEZUELA,T.F.Amaz. Cerro de la Neblina Basecamp. 140 m. 0°50'N, 66°10'W 28 January 1985/ Netted along margins of Rio Baria P.J. & P.M.Spangler, R.Faitoute.W. Steiner/ HOLOTYPE *Hydrodessus kylei* Miller, 2016 [red line with black line border].”

Paratypes, 83 total. **Suriname**, Sipaliwini District, Camp 1, Upper Palumeu, 2.477°N, 55.629°W, 14 Mar 2012, large sandy creek, 275m, A. Short (18, KUNHM, museum numbers in Table 1); same except, Camp 1 on Kutari River, 2.175°N, 56.787°W, 19 Aug 2010, UV light, 275m, A. Short (1, KUNHM, SEMC0915690). **Venezuela**, Amazonas, Cerro de la Neblina Basecamp, 0.833°N, 66.167°W, 20 Feb 1985, Netted along margins of Rio Baria, 140m, P.J. Spangler, P.M.Spangler, R. Faitoute, W. Steiner (50, MIZA, USNM, MSBA, KUNHM); same except 27 Jan 1985 (1, USNM); same except 28 Jan 1985 (13, MIZA, USNM, MSBA, KUNHM).

### Hydrodessus laetus sp. n.

Taxon classificationAnimaliaColeopteraDytiscidae

http://zoobank.org/BC4DDDEE-954B-437F-AA83-05B425E4B244

Figs 22 , 39 , 51

#### Type locality.

Suriname, District Brokopondo, Brownsweg.

#### Diagnosis.

This species is robust and broadly rounded with a distinctive dorsal pattern of maculae and fasciae (Fig. 22A). The head and pronotum are yellow (Fig. 22A). The elytra are dark brown with yellow lateral margins and large, well-defined maculae subbasally, apically and at about 2/3 length of elytra (Fig. 22A). Specimens do not have a lateral elytral carina, the epipleural carina extends nearly straight from the humeral angle (Fig. 22B). The prosternal process is elongate oval with the apex broadly pointed (Fig. 22C). The metaventrite carinae are distinctive and moderately divergent posteriorly. The male median lobe is basally narrowly triangular (Fig. 22D). The apical portion is long and nearly evenly curved with the apex narrow (Fig. 22D). In ventral aspect the median lobe is bilaterally symmetrical with the lateral margins narrowed to narrowly rounded apex (Fig. 22E). The lateral lobe is elongate-triangular with a long series of setae along the dorsal margin (Fig. 22F). This species is similar to *Hydrodessus rattanae* in coloration, overall shape, lack of lateral elytral carinae, shape of the prosternal process and metasternum and other features. The male genitalia are diagnostic (Figs 22D–F). *Hydrodessus rattanae* is also more robust, not as attenuate posteriorly and the color pattern is a little different. The metacoxal lines and regions mediad to the metacoxae are different, too. In *Hydrodessus rattane* the metacoxal lines are shorter, somewhat more divergent anteriorly and there are deep, longitudinal grooves along the medial margin of each metacoxal lines (Fig. 30C) that are missing in *Hydrodessus laetus* (Fig. 22).

#### Description.

*Measurements*. TL = 2.9–3.0 mm, GW = 1.4–1.5 mm, PW = 1.2–1.3 mm, HW = 0.9 mm, EW = 0.5–0.6 mm, TL/GW = 2.0–2.1, HW/EW = 1.7. Body shape broad, posteriorly pointed, outline discontinous between pronotum and elytron (Fig. 22A), body somewhat depressed.

*Coloration* (Fig. 22A). Head and pronotum yellow. Elytra fasciate, brown to brown-red with large irregular yellow regions transversely near anterior margin and medially, apex yellow, macula distinctly delimited (Fig. 22A). Antennae, palps and legs yellow. Ventral sclerites yellow, black along some sutures including metacoxal / abdominal sclerite I, abdominal I / II and the anterior metasternal margin.

*Sculpture and structure*. Head broad, anteriorly broadly rounded; surface shiny with fine mesh of reticulation and few, scattered, fine punctures; eyes large. Pronotum with lateral margins broadly rounded, widest slightly anterior to middle (Fig. 22A); lateral bead fine; surface shiny with fine microreticulation, covered with fine punctures. Elytra broad, lateral margins broadly curved, apically pointed; lateral carina absent, elytral epipleural carina extends directly posteriorly from humeral angle (Fig. 22B); surface with fine microreticulation throughout and covered with fine punctures. Prosternum medially tectiform; prosternal process broad, subrectangular, lateral margins broadly curved, apically broad and truncate, medially broadly excavated (Fig. 22C). Metaventrite with anterior process short and broad, medially distinctly excavated, slightly constricted subapically, apically subtruncate; metasternal carina distinctive, straight and diverging posteriorly, terminating at anterior ends of metacoxal lines (Fig. 22C); surfaces covered with fine punctation. Legs with surfaces shiny, weakly and indistinctly punctate; metatibia with posteroapical brush of setae distinctive; pro- and mesotibiae moderately broad; metatrochanter not strongly offset, elongate, apically narrowly rounded; metacoxa covered with fine punctation; metacoxal lines robust, well marked, broadly separated, subparallel but slightly divergent anteriorly (Fig. 22C). Abdomen covered with fine punctation; apex of VI narrowly rounded.

*Male genitalia*. Median lobe bilaterally symmetrical, in lateral aspect broadly and evenly curved, except apical 1/3 which is relatively straight, basal portion small and subtriangular, apical portion slender to narrowly rounded apex (Fig. 22D); in ventral aspect slender, lateral margins slightly curved, slightly narrowed medially and apex slender and narrowly rounded (Fig. 22E). Lateral lobe slender, elongate, without broad basal region, apex evenly narrowed to narrowly rounded apex (Fig. 22F).

*Female genitalia*. Gonocoxosternite broadly triangular, medial margin linear, apicolateral margin evenly curved, anterior portion large, broad, apically broadly rounded (Fig. 39). Gonocoxa elongate, apical portion elongate, apex narrowly rounded, medial margin curved, anterior apodeme long, as long as apical portion, slender (Fig. 39). Bursa bilaterally symmetrical, elongate slender, medially expanded, apically truncate; spermathecal duct extremely long and slender, expanded near receptacle which is semispherical; spermatheca bulbous with distinctive spermathecal spine; fertilization duct short and long (Fig. 39).

*Sexual dimorphism*. Male pro- and mesotarsi I–III more broadly expanded than female and ventrally with several large adhesive setae.

*Variation*. Specimens exhibit some minor variation in the extend of the maculae on the elytron.

#### Distribution.

This species is known from Venezuela (Fig. 51).

#### Habitat.

Specimens have been collected along a forest river and at lights.

#### Etymology.

This species is named *laetus*, Latin for “colorful,” for the attractive dorsal coloration of specimens.

#### Discussion.

See below under *Hydrodessus rattanae* for additional comments.

#### Specimens.

Holotype in MIZA labeled, “VENEZUELA,T.F.Amaz. Cerro de la Neblina Basecamp, 140 m. 0°50'N. 66°10'W 28 January 1985/ Netted along margin of Rio Baria P.J. & P.M Spangler, R.Faitoute.W.Steiner/ HOLOTYPE *Hydrodessus laetus* Miller, 2016 [red label with black line border].”

Paratypes, 5 total. **Venezuela**, Amazonas, Cerro de la Neblina, basecamp, 0.833°N, 66.167°W, 28 Jan 2985, netted along margins of Rio Baria, 140m, P.J. Spangler, P.M. Spangler, R. Faitoute and W. Steiner (2, USNM); same except 22 Feb 1985, blacklight in rainforest clearing near streams, 140m, P.J. Spangler, P.M. Spangler, R. Faitoute and W. Steiner (1, USNM); same except 6 Feb 2015, blacklight on bank of Rio Baria, 140m, W.E. Steiner (2, USNM).

### Hydrodessus latotibialis sp. n.

Taxon classificationAnimaliaColeopteraDytiscidae

http://zoobank.org/C25750D8-1F4B-422D-A309-AC40C800867E

Figs 23 , 42

#### Type locality.

Peru, Madre de Dios, Rio Tambopata Reserve, 30km SW Puerto Maldonado.

#### Diagnosis.

This species is part of a group including *Hydrodessus maculatus*, *Hydrodessus phyllisae* and *Hydrodessus tenuatus* that have the lateral elytral carina long (half or more the length of the elytron) (Fig. 23B), the prosternal process very broad (length/width < 2) (Fig. 23C), and the metaventral platform (the region between the metaventrite carinae) conspicuously constricted near the base of the metaventral process and fairly broadly divergent posteriorly (Fig. 23C). *Hydrodessus latotibialis* differs from *Hydrodessus maculatus* in having the elytra red with only indistinct, weakly defined pale regions on the elytron (Fig. 23A) and from *Hydrodessus tenuatus* in having the pro- and mesotarsi broad with a subapical emargination (Fig. 7B). From *Hydrodessus phyllisae*, this species differs in size. *Hydrodessus phyllisae* are smaller (TL < 2.7 mm) than *Hydrodessus latotibialis* (Tl > 2.9 mm). Also, specimens are more shiny than *Hydrodessus phyllisae* which are dorsally more matte. Unfortunately, male specimens of *Hydrodessus latotibialis* were not available, so the usually definitive male gentalia were not examined for comparison.

#### Description.

*Measurements*. TL = 3.0–3.2 mm, GW = 1.5 mm, PW = 1.2–1.3 mm, HW = 0.9 mm, EW = 0.5–0.6 mm, TL/GW = 2.0–2.1, HW/EW = 1.7. Body shape moderately robust, apically rounded, lateral margins distinctly discontinuous between pronotum and elytron (Fig. 23A).

*Coloration* (Fig. 23A). Head dark orange. Pronotum orange. Elytron dark orance with broad, indistinct pale areas anteriorly, subapically and at apex. Antennae, palps, and legs orange. Ventral surfaces dark orange.

*Sculpture and structure*. Head broad, anterior margin broadly rounded medially; surface covered with minute punctures; eyes moderately small. Pronotum subcordate, widest slightly anterior of middle (Fig. 23A); lateral bead fine; surface shiny with fine punctures. Elytra elongate, apically rounded (Fig. 23A); lateral carina extending posteriorly to about 1/2 length of elytron (Fig. 23B); surface shiny, covered with fine punctures. Prosternum medially carinate, setose; prosternal process moderately broad, subrectangular but widest at anterior laterally-expanded lobes, lateral margins slightly concave, subparallel, apex truncate, longitudinally strongly impressed (Fig. 23C). Metaventrite with anterior process moderately large, apically rounded, distinctly subapically constricted; metasternal carinae approximated anteriorly, posteriorly well-marked, strongly and evenly divergent across metasternum, ending near anterior terminus of metacoxal lines (Fig. 23C); other surfaces covered with fine punctures. Legs with most surfaces covered with fine punctures; metatibia with distinctive brush of dense, elongate setae on postero-apical surface; pro- and mesotibiae broad, with broad subapical emargination on dorsal margin (Fig. 7B); metatrochanter apically rounded but with small, sharp point; metacoxa evenly covered with fine punctures; metacoxal lines well developed, anteriorly slightly divergent but nearly subparallel (Fig. 23C). Abdomen shiny, evenly covered with fine punctures; apex of VI rounded.

*Male genitalia*. Only females were examined.

*Female genitalia*. Not examined.

*Sexual dimorphism*. Only females were examined.

*Variation*. No signficant variation was detected.

#### Etymology.

This species is named *latotibialis* from the Latin, *lato*, meaning “broad,” and *tibialis*, meaning “tibia,” for the relatively broad mesotibia in specimens.

#### Distribution.

This species is known only from one locality in Tambopata Reserve, Peru (Fig. 42).

#### Habitat.

The type specimens were collected from subtropical moist forest.

#### Discussion.

Two female specimens were examined of this species. Although ordinarily it is ill advised to describe new species of Dytiscidae based only on female specimens, this species appears sufficiently distinct that there should be little difficulty in associating specimens with this species in the future.

#### Specimens.

The holotype and one paratype were examined. The holotype female is in USNM labeled, “PERU: Madre de Dios: Rio Tambopata Res: 30 air km.SW Pto.Maldonado,290m 16–20 XI 1979 J.B.Heppner subtropical moist forest/ HYDRODESSUS sp. P.J.S. [handwritten]/ HOLOTYPE *Hydrodessus latotibialis* Miller, 2016 [red label with black line border].”

Paratype, 1 total. **Peru**, Madre de Dios, Rio Tambopata Reserve, 30km SW Puerto Maldonado, 290m, 16–20 Nov 1979, subtropical moist forest, J.B. Heppner (1, USNM).

### Hydrodessus maculatus sp. n.

Taxon classificationAnimaliaColeopteraDytiscidae

http://zoobank.org/49783BC9-6EA4-45E9-A9BE-071517EE9CDF

Figs 24 , 40 , 45

#### Type locality.

Venezuela, Territorio Federal Amazonas, Cerro de la Neblina, basecamp, 0°50'N 66°10'W.

#### Diagnosis.

This is a distinctive, elongate, dorsally maculate species (Fig. 24A). The dorsal base color is dark black with red areas medially and laterally on the pronotum and as moderately distinctive, irregular maculae subbasally, subapically and apically on the elytron (Fig. 24A). The lateral elytral carina is distinctive to about 1/2 length of elytron (Fig. 24B). The prosternal process is broad with subparallel lateral margins (Fig. 24C). The metaventrite carinae are prominent, constricted anteriorly and evenly divergent posteriorly (Fig. 24C). The male median lobe in lateral aspect is relatively narrow basally and abruptly curved at base of apical portion (Fig. 24D). The apical portion is relatively straight and medially distinctly expanded along ventral margin with the apex elongate, slender and sharply pointed (Fig. 24D). The median lobe in ventral aspect is slender to a distinct subapical lateral expansion with the apex convergent to a rounded apex (Fig. 24E). The lateral lobe is moderately slender and curved to rounded apex (Fig. 24F). The series of apical setae are on the ventral margin rather than the dorsal as in other species (Fig. 24F).

#### Description.

*Measurements*. TL = 3.0–3.1 mm, GW = 1.4 mm, PW = 1.2 mm, HW = 0.8–0.9 mm, EW = 0.5 mm, TL/GW = 2.2, HW/EW = 1.7. Body shape moderately elongate, lateral margin distinctly discontinuous between pronotum and elytron (Fig. 24A).

*Coloration* (Fig. 24A). Head reddish. Pronotum yellow, reddish medially and along posteromedial margin. Elytra red-brown with three yellow regions (Fig. 24A), one sub-basally with large, irregular macula extending from lateral margin to near suture, one irregular macula at about 2/3 length, and one at apex.

*Sculpture and structure*. Head broad, anterior clypeal margin subtruncate; surface covered with fine punctures; eyes moderately large (Fig. 24A). Pronotum cordate (Fig. 24A), lateral margins broadly curved in anterior half, slightly convergent posteriorly in posterior half; lateral bead fine anteriorly, posteriorly obscured; surface covered with fine punctures. Elytra elongate, lateral margins subparallel for much of length, apex somewhat pointed (Fig. 24A); lateral carina distinct and conspicuous, extending to near half length of elytron (Fig. 24B); surface of elytron covered with fine punctures. Prosternum medially prominently carinate with fine setae on each side; prosternal process broad, broadest anteriorly, lateral margins slightly convex, strongly impressed medially, apex broadly truncate (Fig. 24C). Metaventrite with metasternal process well developed, conspicuously constricted subapically, surface slightly excavated, carinae well-developed, divergent posteriorly, extending to posterior margin of Metaventrite and ending near anterior ends of metacoxal lines (Fig. 24C); Metaventrite covered with fine punctures. Legs with surfaces covered with fine punctures; metatibia with posteroapical brush of setae; pro and mesotibiae moderately broad; metacoxa very densely covered with fine punctures; metacoxal lines well developed, straight and subparallel, only slightly divergent anteriorly (Fig. 24C). Abdomen densely, finely punctate; stermine VI apically broadly rounded.

*Male genitalia*. Median lobe bilaterally symmetrical, in lateral aspect moderately curved, with basal portion subtriangular, apical portion curved medially, more straight near apex, subapically somewhat expanded along ventral margin, strongly tapered to elongate, pointed apex (Fig. 24D); in ventral aspect with lateral margins subparallel for most of length, subapically distinctly expanded laterally and apex broadly triangular (Fig. 24E). Lateral lobe moderately broad basally, evenly tapered and slightly curved to rounded apex which has series of marginal setae (Fig. 24F).

*Female genitalia*. Gonocoxosternite with apical portion broadly triangular, medial margin slightly concave, apical portion very large and broadly lobed (Fig. 40). Gonocoxa broad, apically broadly rounded, evenly tapered anteriorly to short apodeme (Fig. 40). Bursa short, broad; spermathecal duct very slender and elongate, expanded to receptacle which is semispherical; spermatheca elongate, slender and curved, without spermathecal spine; fertilization duct short, slender (Fig. 40).

*Sexual dimorphism*. Male pro- and mesotarsi I–III more broadly expanded than female and ventrally with several large adhesive setae.

*Variation*. Specimens vary in coloration with some specimens darker and others lighter.

#### Etymology.

This species is named *maculatus*, Latin for “spotted,” for the maculate coloration on the elytra in specimens.

#### Distribution.

*Hydrodessus maculatus* is known from Amazonas, Venezuela and Region IX, Guyana (Fig. 45).

#### Habitat.

Specimens were collected “seined from rocks in rapids” and “netted along margins” of the Rio Baria. They have also been found in creeks and at a blacklight in a rainforest.

#### Specimens.

Holotype: ♂ in MIZA labeled, “VENEZUELA, T.F.Amaz. Cerro de la Neblina Basecamp, 140 m. 0°50'N, 66°10'W 20 February 1985/ Netted along margins of Rio Baria P.J. & P.M.Spangler, R.Faitoute,W.Steiner/ HOLOTYPE Hydrodessus maculatus Miller, 2016 [red label with black line border].”

Paratypes, 110 total. **Venezuela**; Amazonas, Cerro de al Neblina, basecamp, 0.833°N, 66.167°W, 21 Feb 1985, muddy oxbow pond, 140m, P.J. Spangler, P.M. Spangler, R. Faitoute and W. Steiner (51, USNM, KUNHM, MIZA, MSBA); same, but 20 Feb 1985, netted along margins of Rio Baria (51, USNM, KUNHM, MIZA, MSBA); same, but 20 Feb 1985, seined from rocks in rapids of Rio Baria (4, USNM); same, but 6 Feb 2013, blacklight in rainforest clearing near Rio Baria (1, USNM). **Guyana**; Region IX, road to Parabara, creek crossing at Mushal Wao, 2.161°N, 59.292°W, 1 Sep 2013, creek margins, 268m, Short, Isaacs, Salisbury (2, KUNHM, SEMC0964975, SEMC0964987).

### Hydrodessus morsus sp. n.

Taxon classificationAnimaliaColeopteraDytiscidae

http://zoobank.org/32C68BA3-78DC-464D-AD0C-28C975DA8156

Figs 25 , 50

#### Type locality.

Venezuela, Amazonas, Cerro de la Neblina, basecamp, 0.833°N, 66.167°W.

#### Diagnosis.

This is the smallest *Hydrodessus* (TL < 1.5 mm). In addition, this species differs in having a low and rounded lateral elytral carina (Fig. 25B), a relatively narrow and apically pointed prosternal process (Fig. 25C), and the metaventrite carinae poorly developed (Fig. 25C). Specimens are concolorous yellow and parallel-sided (Fig. 25A). The male median lobe in lateral aspect is moderately broad basally, weakly curved and apically pointed (Fig. 25D). In ventral aspect the median lobe is slightly constricted subapically and apically rounded (Fig. 25E). The lateral lobe is broad basally and with margins apically evenly convergent to rounded apex (Fig. 25F).

#### Description.

*Measurements*. TL = 1.4–1.6 mm, GW = 0.5–0.6 mm, PW = 0.5–0.6 mm, HW = 0.4–0.5 mm, EW = 0.3 mm, TL/GW = 2.4–2.6, HW/EW = 1.6–1.7. Body elongate, parallel–sided, lateral margin distinctly discontinuous between pronotum and elytron, dorsoventrally somewhat compressed (Fig. 25A).

*Coloration* (Fig. 25A). Body surfaces yellow throughout.

*Sculpture and structure*. Head elongate, anterior clypeal margin broadly rounded; surface finely punctate and shiny; eyes moderately large and large-faceted. Pronotum cordate, widest anterior to middle (Fig. 25A); lateral bead very fine; surface shiny with fine punctation. Elytra elongate, lateral margins subparallel (Fig. 25A); lateral carina indistinct, rounded, present only at humeral angle (Fig. 25B); surface covered with fine punctures. Prosternum relatively flat, not medially carinate; prosternal process elongate triangular, lateral carinae convergent to narrowly pointed apex, apically narrowly separated from Metaventrite (Fig. 25C). Metaventrite with anterior process narrowly triangular, metasternal carinae represented by low, rounded margin of medial flattened surface extending posteriorly to anterior ends of metacoxal lines (Fig. 25C); surface covered with fine punctation. Legs with surfaces largely shiny and impunctate; pro- and mesotibiae moderately broad; metatrochanter strongly offset and apically rounded; metatibia with posteroapical brush of setae; metacoxa covered with fine punctation; metacoxal lines indistinct, medially somewhat approximated, anteriorly divergent (Fig. 25C). Abdominal ventrites covered with fine punctation; VI apically broadly rounded.

*Male genitalia*. Median lobe bilaterally symmetrical, in lateral aspect broadly curved, with very broad basal portion, with medial expansion along ventral margin, apically with dorsal margin nearly stright, dorsal margin broadly curved to pointed apex (Fig. 25D); in ventral aspect broad, lateral margins slightly expanded medially, apically with margins slightly convergent to broadly rounded apex (Fig. 25E). Lateral lobe very broad basally, medially curved, apex broad with lateral margins straight and convergent to broadly rounded apex which has small cluster of marginal setae (Fig. 25F).

*Female genitalia*. Not examined.

*Sexual dimorphism*. Male pro- and mesotarsi I–III more broadly expanded than female and ventrally with several large adhesive setae.

*Variation*. Very little variation was observed among the few specimens examined.

#### Etymology.

This species is named *morsus*, Latin for “little bit,” for the small size of specimens.

#### Distribution.

This species is found only in Amazonas, Venezuela (Fig. 50).

#### Habitat.

Nearly all the known specimens were collected at black light.

#### Discussion.

This extremely small *Hydrodessus* has only weakly developed lateral elytral carina and metaventrite carinae. The prosternal process is also relatively narrow. Together, these make this species only poorly placed in *Hydrodessus*, but the male lateral lobes have a single segment, and the overall body shape is consistent with the variation present in the genus. Even so, it is certainly possible this species does not belong in *Hydrodessus*.

#### Specimens.

The holotype male is in MIZA labeled, “VENEZUELA,T.F.Amaz. Cerro de la Neblina Basecamp. 140 m. 0°50'N, 66°10'W 7 February 1985/ At black light on bank of Rio Baria W. E. Steiner, collector/ HOLOTYPE *Hydrodessus morsus* Miller, 2016 [red line with black line border].”

Paratypes, 5 total. **Venezuela**, Amazonas, Cerro de la Neblina, basecamp, 0.833°N, 66.167°W, 7 Feb 1985, black light on bank of Rio Baria, 140m, W.E. Steiner (4, MIZA, USNM); Amazonas, San Fernando de Atabapo, El Pozo, 4.024°N, 67.684°W, 8 Apr 1988, M. Aleman (1, USNM).

### Hydrodessus nanayensis

Taxon classificationAnimaliaColeopteraDytiscidae

Spangler, 1966

Figs 3 , 45

Hydrodessus nanayensis Spangler, 1966: 382; Young 1969: 2; 1970: 158; Spangler 1985: 89; Biström 1988: 37; Nilsson 2001: 236.

#### Type locality.

Peru, near Ituitos, from the Nanay.

#### Diagnosis.

This species is very similar to (or possibly identical with) *Hydrodessus siolii*. Putative differences based on information presented by Spangler (1966; 1985) include a more deeply impressed prosternal process, a more distinctly grooved medial metacoxal region (between the metacoxal lines) and a fasciate color pattern in *Hydrodessus nanayensis* (Fig. 3). Since the type specimen of *Hydrodessus nanayensis* was not examined, other potentially diagnostic features were unavailable.

#### Description.

*Measurements*. TL = 2.95 mm, GW = 1.35 mm. Body elongate, apically narrowly rounded, lateral outline distinctly discontinous between pronotum and elytron (Fig. 3).

*Coloration* (Fig. 3). Head and pronotum testaceous. Elytra testaceous except dark reddish-brown medial stripe along suture and three approximately transverse bands, one basal band extending across 2/3 elytral width, a medial band nearly reaching elytral margin and with elongate expansion medially narrowly separated from expansions of basal and apical bands, and apical maculate region (Fig. 3). Antennae, palps, legs and ventral surfaces testaceous.

*Sculpture and structure*. Head moderately broad, anterior clypeal margins arcuately emarginate; surface with fine, sparse, seta-bearing punctures, most dense between bases of eys, anterior portion nearly impunctate, microreticulate posterior to eyes. Pronotum cordate, widest near anterior margin (Fig. 3); lateral bead fine and distinct throughout length; surface with coarse, seta-bearing punctures separated by 1–2 × pore diameter, more coarse and dense near base. Elytra elongate, apically narrowly rounded (Fig. 3); lateral carina absent; surface finely alutaceous and densely, coarsely punctate, punctures separated by 1–2 × puncture diameter. Prosternal process slender between procoxae, width of apical portion about 2.5 × width between procoxae, weakly longitidinally concave. Legs alutaceous; metatrochanter somewhat swollen; metacoxa covered with coarse, dense, widely-spaced punctures. Abdomen with first abdominal ventrite with coarse, dense, widely-spaced punctures.

*Male genitalia*. Male unknown.

*Female genitalia*. Not described by Spangler (1966).

*Sexual dimorphism*. Male unknown.

*Variation*. Only a single female specimen has been described (Spangler 1966).

#### Distribution.

This species is known only from the type locality near Iquitos, “from the Nanay,” Peru (Fig. 45, Spangler 1966).

#### Habitat.

The single specimen was found “from the Nanay,” which is a large tropical river, though it is not clear that the specimen was specifically collected from the river or, instead, from the region.

#### Discussion.

The specimen on which this species was based was collected during the Catherwood Foundation expedition to Peru. The type material was not found in either the Academy of Natural Sciences of Philadelphia (ANSP, where Spangler indicated the holotype was deposited, J. Weintraub, pers. comm.), the MZCZ (where many ANSP Coleoptera types were sent), or the USNM (where Spangler was working). The species was described from a single female specimen, and an illustration of the habitus was provided (Fig. 3). The description of the species is extensive, though it excludes certain important diagnostic features. The description presented here is based on Spangler’s (1966) description and his figure as well as later keys (Spangler 1985; Young 1970). The shape of the beetle and the color pattern on the elytron are moderately distinctive (Fig. 3), and *Hydrodessus nanayensis* does not appear to correspond to any specimens examined during this study. The apparent loss of the type specimen or the fact that it is a female may make determining to what species this name refers difficult.

Young (1970) thought that *Hydrodessus nanayensis* is likely conspecific with *Hydrodessus siolii*, or, at most, a subspecies. The descriptions are very close and the shape and color pattern are very similar, but without examination of the type of *Hydrodessus nanayensis*, this cannot be determined conclusively.

#### Specimens.

No specimens were examined of this species, and the treatement here is based on the description by Spangler (1966).

### Hydrodessus octospilus

Taxon classificationAnimaliaColeopteraDytiscidae

(Guignot, 1957)

Figs 26 , 48

Brinckius octospilus Guignot, 1957: 39.
Hydrodessus octospilus , Young 1969: 2; 1970: 157; Spangler 1985: 89; Biström 1988: 37; Nilsson 2001: 236.
Hydrodessus robinae Spangler, 1985: 85; Biström 1988: 37; Nilsson 2001: 236.

#### Type locality.

*Brinckius octospilus*, Brazil, Para Province, Cachimbo. *Hydrodessus robinae*, Guyana, Mazaruni-Potaro District, Takutu Mountains, 6°15'N 59°5'W.

#### Diagnosis.

This is a relatively compact species with the dorsal coloration ranging from red to red-brown, sometimes with larger, indistinct pale areas or smaller, more distinctive pale regions (Fig. 26A). The lateral elytral carina is well-developed, extending beyond half the length of the elytron and with a distinct, impressed interruption at about half its length (Fig. 26B). The prosternal process is broad with the lateral margins subparallel and the apex broadly truncate (Fig. 26C). The metaventrite carinae are very well developed, not strongly constricted anteriorly, and evenly divergent posteriorly (Fig. 26C). The male median lobe in lateral aspect is triangular basally with the apical portion somewhat evenly curved with the apex subapically constricted and pointed (Fig. 26D). In ventral aspect the male median lobe is relatively broad with the lateral margins evenly convergent to a pointed apex (Fig. 26E). The lateral lobe is relatively narrow with the lateral margins straight and evenly convergent to the rounded apex (Fig. 26F).

#### Description.

*Measurements*. TL = 2.9 mm, GW = 1.4 mm, PW = 1.2 mm, HW = 0.9 mm, EW = 0.5 mm, TL/GW = 2.1, HW/EW = 1.6–1.7. Body shape moderately robust, apically pointed, lateral margins only somewhat discontinuous between pronotum and elytron (Fig. 26A).

*Coloration* (Fig. 26A). Head and pronotum orange-red. Elytron with base color red, with large, very diffuse pale areas anteriorly, subapically and at apex. Antennae and palps orange. Legs orange-red. Venter yellow-brown, red medially on surfaces, some areas nearly black including portions of prosternal and mesosternal processes and basal abdominal sutures.

*Sculpture and structure*. Head broad, anterior clypeal margin broadly curved; surface shiny with few, sparse minute punctures; eyes large. Pronotum subcordate, widest anterior of middle (Fig. 26A); lateral bead fine and continuous; surface shiny, covered with minute punctures, larger along anterior margin. Elytra moderately elongate, apically narrowly rounded (Fig. 26A); lateral carina distinctive and prominent, extending well beyond ½ length of elytron, slightly but distinctly impressed and interrupted near half its length; surface covered with minute punctures. Prosternum medially carinate and setose; prosternal process broad, with prominent anterolateral angles, lateral margins subparallel, apex broadly truncate, longitudinally strongly impressed (Fig. 26C). Metaventrite with anterior process moderately broad, laterally rounded, apex slightly truncated, medially flat; metasternal carinae flattened and broad, straight and divergent to posterior margin, terminating near anterior ends of metacoxal lines (Fig. 26C). Legs with most surfaces covered with fine punctures; metatibia with distinctive brush of dense, elongate setae on postero-apical surface; pro- and mesotibiae moderately broad; metatrochanter not strongly offset, apically pointed; metacoxa evenly covered with fine punctures; metacoxal lines broadly separated, somewhat sinuate and slightly divergent anteriorly (Fig. 26C). Abdomen shiny, evenly covered with fine punctures; apex of VI broadly pointed.

*Male genitalia*. Median lobe bilaterally symmetrical, in lateral aspect robust, moderately curved, basal portion broad, but not large, apical portion more straight, apex narrowed to slightly curved, nearly pointed apex (Fig. 26D); in ventral aspect broad, lateral margins broadly rounded, apex narrowed to narrowly rounded apex (Fig. 26E). Lateral lobe broad basally, apical portion elongate triangular, lateral margins straight and evenly convergent to rounded apex, with seta along apical margin (Fig. 26F).

*Female genitalia*. Not examined.

*Sexual dimorphism*. Male pro- and mesotarsi I–III slightly more broadly expanded than female and ventrally with several large adhesive setae. Female with abdominal ventrite VI slightly impressed on each side, apicomedially flattened and pointed; male with VI apically rounded, not impressed.

*Variation*. Specimens vary in extent of the dorsal maculae and intensity of dorsal coloration from nearly immaculate to distinctly maculate with larger pale regions.

#### Distribution.

This species is known from Guayana and southern Venezuela to Brazil and south to Paraguay (Fig. 48).

#### Habitat.

*Hydrodessus octospilus* has been collected from blacklights and forested creek and river margins.

#### Discussion.

Examination of the male holotype specimens of *Hydrodessus octospilus* and *Hydrodessus robinae* indicates that these two names refer to the same species. Spangler (1985) erected *Hydrodessus robinae* in part based on it having a longer lateral elytral carina compared with *Hydrodessus octospilus*, but this is really not the case. The type specimen of a *Hydrodessus octospilus* has the lateral carina extending distinctly beyond half the length of the elytron similar to the type of *Hydrodessus robinae*. Also, the male genitalia of the two holotypes are extremely similar. Two female specimens from Paraguay (FSCA) are here assigned to this species. Though this is well south of the range of other, more definite *Hydrodessus octospilus*, they do appear to be *Hydrodessus octospilus*.

#### Specimens.

The *Hydrodessus octospilus* male holotype in MZSP was examined, labeled, “Type [red label with black line border]/ Brasilien, Para Cochimbo X.1955 Pereira [black line border]/ F. Guignot det., 1956 Brinckius octospilus n.sp. Type ♂ [handwritten]/ 31904.”

The *Hydrodessus robinae* male holotype in USNM was examined, labeled, “GUYANA: Mazaruni- Potaro District Takutu Mountains 6°15'N,59°5'W 17 December 1983/ EARTHWATCH Research Expedition; P.J. Spangler. R.A. Faitoute/ HOLOTYPE Hydrodessus robinae PJ Spangler [red label]/ BLNO 003806 [blue label with black line around margin].”

Additional non-type material examined (15 total). **Guyana**, Mazaruni-Potaro District, Takutu Mountains, 6.25°N, 59.083°W, 17 Dec 1983, blacklight in forest clearing near streams, P.J. Spangler, W.E. Steiner (1, USNM, *Hydrodessus robinae* paratype); Region IX, road to Parabara, creek crossing at Mushal Wao, 2.161°N, 59.292°W, 1 Sep 2013, creek margins, 268m, Short, Isaacs, Salisbury (8, KUNHM, collection number in Table 1). **Paraguay**, Paraguari, Arroyo Minas, Parque Nacional Ybycui, 25 Jul 1981, R. Cave (1, FSCA); same except 25kmb SE Ybycui, Arroyo Minas in Parque Nacional Ybycui, 24 Jan 1981, R.D. Cave (1, FSCA). **Venezuela**, Amazonas, Cerro de la Neblina, basecamp, 0.833°N, 66.167°W, 20 Feb 1985, netted along margins of Rio Baria, 140m, P.J. Spangler, P.M. Spangler, R. Faitoute, W. Steiner (2, USNM); same except 6 Feb 1985, blacklight on bank of Rio Baria, W.E. Steiner (1, USNM); same except 27 Jan 1985 (1, USNM).

### Hydrodessus palus sp. n.

Taxon classificationAnimaliaColeopteraDytiscidae

http://zoobank.org/D8C3FC2E-1B5E-4F6E-946D-7F2B2E5C797F

Figs 27 , 42

#### Type locality.

Venezuela, Amazonas State, Communidad Cano Gato, Rio Sipapo, 4.981°N, 67.739°W.

#### Diagnosis.

This species has the lateral elytral carina relatively short, present just at the humeral angle (Fig. 27B), and the body overall nearly concolorous except somewhat darker on the apical half of the elytron (Fig. 27A). *Hydrodessus palus* is similar to *Hydrodessus brevis* in shape, and other structures, but that species is dark red (Fig. 12A) and *Hydrodessus palus* is pale yellow (Fig. 27A), and that species is a bit larger (TL = 2.5 mm) with *Hydrodessus palus* smaller (TL = 2.0 mm). The male genitalia differ, as well, with the median lobe of *Hydrodessus palus* more slender and apically sinuate (Fig. 27E).

#### Description.

*Measurements*. TL = 2.1–2.2 mm, GW = 0.8–0.9 mm, PW = 0.8 mm, HW = 0.6 mm, EW = 0.3 mm, TL/GW = 2.3, HW/EW = 1.9–2.0. Body very elongate, lateral margin distinctly discontinuous between pronotum and elytron (Fig. 27A).

*Coloration* (Fig. 27A). Body surfaces all yellow.

*Sculpture and structure*. Head broad, apically rounded; anterior clypeal margin broadly curved; surface with few, fine punctures; eyes large. Pronotum cordate, widest anterior to middle (Fig. 27A); lateral bead slender anteriorly, obscured posteriorly; surface shiny with fine punctures. Elytra elongate, laterally evenly rounded, apically pointed; lateral carina indistinct, rounded, present only near humeral angle (Fig. 27B); elytral surface shiny with fine punctation. Prosternum evenly rounded medially, weakly tectiform; prosternal process elongate, broad near base, extending laterally in rounded lobes, posteriorly slender, lateral carinae convergent posteriorly, medially longitudinally somewhat depressed, apex narrowly rounded (Fig. 27C). Metaventrite with anterior process slender, carinae not strongly developed, posteriorly diverging, represented by low, broad ridges ending near anterior ends of metacoxal lines (Fig. 27C); covered with irregular punctures. Legs with surfaces covered with fine punctures; pro- and mesotibiae moderately broad; metatrochanter strongly offset from metafemur, apex distinctly pointed; metatibia with posteroapical brush of setae; metacoxa covered with irregular punctures; metacoxal lines broadly separated, slightly curved, nearly straight, anteriorly slightly divergent (Fig. 27C). Abdomen covered with irregular punctation; abdominal ventrite VI apically evenly rounded.

*Male genitalia*. Median lobe bilaterally symmetrical, in lateral aspect somewhat curved, with basal portion narrowly triangular, apical portion very slender, broadly curved, apex slightly sinuate and narrowly pointed (Fig. 27D); in ventral aspect moderately slender, lateral margins broadly curved, constricted subapically with apex narrowly rounded (Fig. 27E). Lateral lobe moderately broad basally, long and elongate triangular with lateral margins evenly convergent to narrowly rounded apex which has a series of marginal setae (Fig. 27F).

*Female genitalia*. Not examined.

*Sexual dimorphism*. Male pro- and mesotarsi I–III more broadly expanded than female and ventrally with several large adhesive setae.

*Variation*. Only two specimens were examined, and there is no significant variation between them.

#### Etymology.

This species is named *palus*, Latin for “pale,” for the overall yellow coloration of specimens.

#### Distribution.

*Hydrodessus palus* is known only from the type locality in northwestern Amazonas, Venezuela (Fig. 42).

#### Habitat.

The two known specimens were collected from a sandy forest stream with considerable plant material (leaves, branches, etc) in the margins.

#### Specimens.

The male holotype specimen is in MIZA labeled, “VENEZUELA: Amazonas State 4°58.838'N, 67°44.341'W; 95m Communidad Caño Gato, on Rio Sipapo; 16.i.2009; leg. Short, Miller, Camacho, Joly, & García VZ09-0116-01X; along stream/ SM0842821 KUNHM-ENT [barcode label]/ HOLOTYPE Hydrodessus palus Miller, 2016 [red label with black line border].”

Paratypes, 1 total. **Venezuela**, Amazonas, Communidad Caño Gato, on Rio Sipapo, 4.980°N, 67.739°W, 16 Jan 2009, along stream, 95m, Short, Miller, Camacho, Joly, Garcia (1, KUNHM, SM0842840).

### Hydrodessus peloteretes

Taxon classificationAnimaliaColeopteraDytiscidae

Spangler, 1985

Figs 28 , 42

Hydrodessus peloteretes Spangler, 1985: 80; Biström 1988: 37; Nilsson 2001: 236.

#### Type locality.

Guyana, Mazaruni-Potaro District, Takutu Mountains, 6°15'N, 59°5'W.

#### Diagnosis.

This species is largely red dorsally (Fig. 28A). The pronotum is yellow and the elytral apex is yellow as is a poorly-defined macula at about 2/3 length of elytron (Fig. 28A). The pronotum is not as broadly curved as most species, and is broadest near the posterior angles with its greatest width distinctly less than the greatest width across the elytra (Fig. 28A). The prosternal process is relatively narrow with distinctive lateral lobes anteriorly (Fig. 28C). The metaventrite carinae are strongly divergent posteriorly and each carina is expanded near the posterior apex (Fig. 28C). The male median lobe is broadly triangular basally, sharply curved near base of apical portion, and apically sinuate (Fig. 28D). In ventral aspect the median lobe is bilaterally symmetrical with the lateral margins subparallel and the apex rounded (Fig. 28E). The lateral lobe is very broad with the apex narrowly lobate (Fig. 28F).

#### Description.

*Measurements*. TL = 2.7 mm, GW = 1.3 mm, PW = 1.0 mm, HW = 0.7 mm, EW = 0.5 mm, TL/GW = 2.2, HW/EW = 1.6. Body shape elongate, lateral margin evenly concavely curved between pronotum and elytron, apically pointed (Fig. 28A).

*Coloration* (Fig. 28A). Head yellow-brown. Pronotum yellow. Elytron yellow brown with vague pale macula subapically and apex yellow. Antennae, palps and legs yellow. Venter yellow to yellow-brown, darker brown on Metaventrite and other thoracic ventrites.

*Sculpture and structure*. Head broad, anterior clypeal margin evenly curved; surface shiny, covered with minute punctures; eyes moderately small. Pronotum with lateral margins more strongly curved anteriorly, but pronotum widest at posterolateral angles; lateral bead very fine, but continuous; surface shiny, covered with minute punctures. Elytra elongate, apically pointed (Fig. 28A); lateral carina indistinct, rounded, evident only at humeral angle (Fig. 28B); surface covered with minute, dense punctation. Prosternum medially tectiform and setose; prosternal process broadest at anterior, laterally expanded lobes, lateral margins concave, distinctly convergent to rounded apex, longitudinally excavated (Fig. 28C). Metaventrite with anterior process moderately slender, apex narrowly truncate, distinctly expanded subapically with prominent lateral lobes, medially somewhat impressed; metasternal carina distinct anteriorly, closely approximated, posteriorly obsolete, represented by strongly diverging lines of impunctate surface (Fig. 28C); other surfaces covered with fine punctation. Legs with most surfaces covered with fine, irregular punctation; metatibia with distinctive brush of dense, elongate setae on postero-apical surface; pro- and mesotibiae moderately slender; metatrochanter somewhat offset, apically broadly pointed; metacoxa evenly covered with fine punctures; metacoxal lines broadly separated, broadly divergent anteriorly (Fig. 28C). Abdomen shiny, evenly covered with fine punctures, apically somewhat rugulose; apex of VI rounded.

*Male genitalia*. Median lobe bilaterally symmetrical, in laterl aspect very broadly curved, broad basally, strongly constricted medially, more expanded, but slender in apical half, sinuate with apex slender and pointed (Fig. 28D); in ventral aspect nearly parallel-sided, moderately broad with apex broadly rounded (Fig. 28E). Lateral lobe very broad, terminating in small, slender, slightly curved lobe, with two patches of setae, apically on lobe, and subapically along ventral margin (Fig. 28F).

*Female genitalia*. Not examined.

*Sexual dimorphism*. Only the male holotype examined.

*Variation*. Only the male holotype examined.

#### Distribution.

This species is known only from the Takutu Mountains of northern Guyana (Fig. 42).

#### Habitat.

The single known specimen was collected from a blacklight in a forest clearing near some streams.

#### Specimens.

The holotype male in USNM was examined, it is labeled, “GUYANA: Mazaruni- Potaro District Takutu Mountains 6°15'N,59°5'W 17 December 1983/ EARTHWATCH Research Expedition: P. J. Spangler & W. E. Steiner Collectors/ At blacklight in forest clearing near streams/ HOLOTYPE Hydrodessus peloteretes PJ Spangler [red label]/ BLNO 003804 [blue label with black line around margin].”

### Hydrodessus pereirai

Taxon classificationAnimaliaColeopteraDytiscidae

(Guignot, 1957)

Figs 4 , 45

Brinckius pereirai Guignot, 1957: 41.
Hydrodessus pereirai , Young 1969: 2; 1970: 157; Spangler 1985: 88; Biström 1988: 37; Nilsson 2001: 236.

#### Type locality.

Brazil, Pará State, Cachimbo.

#### Diagnosis.

Specimens of this species are among the largest *Hydrodessus* (TL = 3.9). The lateral elytral carina is distinctly present only at the humeral angle, though it can be traced further along the elytron out to about 1/4 ts length (Fig. 4B), the dorsal and ventral coloration is approximately evenly dark red (Fig. 4A), the lateral pronotal margins are broadly curved (Fig. 4A), the prosternal process is broad with distinctive lateral lobes anteriorly (Fig. 4C), the metaventrite carinae are moderately distinctive, not strongly constricted, and moderately divergent posteriorly (Fig. 4C), the metacoxal lines are broadly separated and somewhat divergent anteriorly (Fig. 4C). *Hydrodessus pereirai* is superficially very similar to *Hydrodessus brevis* in coloration, shape, and general structures, but that species has a maximum length of about 2.5mm. Unfortunately, males are not known for *Hydrodessus pereirai* so the usually definitive character system of male genitalia is not available for comparison.

#### Description.

*Measurements*. TL = 3.8 mm. Body elongate, apically pointed, lateral margin strongly discontinuous between pronotum and elytron (Fig. 4A).

*Coloration* (Fig. 4A). Head, pronotum and elytra evenly dark red. Antennae and palps yellow-red. Legs yellow-brown to yellow-red. Venter dark red, yellow-red at apex of abdomen.

*Sculpture and structure*. Head broad, anterior margin slightly flattened medially; surface covered with minute punctures; eyes moderately small. Pronotum cordate, widest anterior to middle (Fig. 4A); lateral bead fine, but distinct; surface covered with fine punctation. Elytra long, apically pointed; lateral carina distinctive, extending about 1/3 length of elytron (Fig. 4B); surface covered with minute punctation. Prosternum medially with broad, rounded ridge; prosternal process broad, broadest at anterior laterally expanded lobes, lateral margins slightly convergent to slightly curved, subtruncate apex, medially distinctly impressed (Fig. 4A). Metaventrite with anterior process elongate, moderately slender, flattened; metasternal carina distinctive, straight from sides of process, evenly divergent across Metaventrite (Fig. 4C); surfaces covered with minute punctures. Legs with most surfaces covered with fine punctation; pro- and mesotibiae slender; metatibia with distinctive brush of dense, elongate setae on postero-apical surface; metatrochanter distinctly offset, apically narrowly rounded; metacoxa with surface covered with minute punctures; metacoxal lines broadly separated, slightly sinuate and divergent anteriorly (Fig. 4C). Abdomen covered with minute punctures; abdominal ventrite VI with apex slightly pointed.

*Female genitalia*. Not examined.

*Sexual dimorphism*. Only one, female specimen (the holotype) was examined.

*Variation*. Only one, female specimen (the holotype) was examined.

#### Distribution.

The species is known only from the type locality in Para, central Brazil (Fig. 45).

#### Habitat.

Nothing is known of the habitat of this species.

#### Specimens.

Only the female holotype in MZSP was examined, labeled, “Type [red label with black border]/ Brasilien, Para Cachimbo X.1955 Pereira/ F. Guignot det., 1956 Brinckius Pereirai Type ♀ [handwritten]/ 31901.”

### Hydrodessus phyllisae

Taxon classificationAnimaliaColeopteraDytiscidae

Spangler, 1985

Figs 7A , 29 , 41 , 47

Hydrodessus phyllisae Spangler, 1985: 86; Biström 1988: 37; Nilsson 2001: 236.

#### Type locality.

Guyana, Mazaruni-Potaro District, Takutu Mountains, 6°15'N 59°5'W

#### Diagnosis.

This species is part of a group including *Hydrodessus maculatus*, *Hydrodessus latotibialis* and *Hydrodessus tenuatus* that have the lateral elytral carina long (half or more the length of the elytron) (Fig. 29B), the prosternal process broad (length/width < 2) (Fig. 29C), and the metaventral platform (the region between the metaventrite carinae) conspicuously constricted near the base of the metaventral process and broadly divergent posteriorly (Fig. 29C). *Hydrodessus phyllisae* differs from *Hydrodessus maculatus* in having the elytra red with only indistinct, weakly defined pale regions on the elytron (Fig. 29A), and from *Hydrodessus tenuatus* in having the pro- and mesotarsi broad with a subapical emargination (Fig. 7A). From *Hydrodessus latotibialis*, this species differs in size. *Hydrodessus phyllisae* are smaller (TL < 2.7 mm) than *Hydrodessus latotibialis* (Tl > 2.9 mm). Also, specimens are more matte than *Hydrodessus latotibialis* which are dorsally shiny. Unfortunately, male specimens of *Hydrodessus latotibialis* were not available, so the usually definitive male gentalia were not examined for comparison.

#### Description.

*Measurements*. TL = 2.5–2.6 mm, GW = 1.2 mm, PW = 1.0 mm, HW = 0.7 mm, EW = 0.4 mm, TL/GW = 2.1–2.2, HW/EW = 1.7–2.0. Body shape moderately robust, apically rounded, lateral margins distinctly discontinuous between pronotum and elytron (Fig. 29A).

*Coloration* (Fig. 29A). Head orange. Pronotum yellow. Elytron yellow brown with vague pale areas anteriorly, laterally, subapically and at apex. Antennae, palps, and legs yellow. Venter yellow-brown, lighter on prothorax and epipleuron.

*Sculpture and structure*. Head broad, anterior margin subtruncate medially; surface covered with minute punctures; eyes moderately small. Pronotum subcordate, widest near middle (Fig. 29A); lateral bead fine, somewhat obscured anteriorly; surface shiny with fine punctures. Elytra elongate, apically rounded (Fig. 29A); lateral carina distinct near humeral angle, extending as low, indistinct ridge posteriorly to about 1/2 length of elytron (Fig. 29B); surface shiny, covered with fine punctures. Prosternum medially carinate, setose; prosternal process moderately broad, subrectangular but widest at anterior laterally-expanded lobes, lateral margins slightly concave, subparallel, apex shallowly rounded, longitudinally strongly impressed (Fig. 29C). Metaventrite with anterior process moderately large, apically rounded, distinctly subapically constricted; metasternal carinae narrow anteriorly, posteriorly well-marked, strongly and evenly divergent across metasternum, ending near anterior terminus of metacoxal lines (Fig. 29C); other surfaces covered with fine punctures. Legs with most surfaces covered with fine punctures; metatibia with distinctive brush of dense, elongate setae on postero-apical surface; pro- and mesotibiae broad, with broad subapical emargination on dorsal margin; metatrochanter apically rounded but with small, sharp point; metacoxa evenly covered with fine punctures; metacoxal lines well developed, anteriorly slightly divergent but nearly subparallel (Fig. 29C). Abdomen shiny, evenly covered with fine punctures; apex of VI rounded.

*Male genitalia*. Median lobe bilaterally symmetrical, in lateral aspect abruptly and broadly curved, very broad basally, apical portion constricted, slightly expanded along ventral margin, and relatively straight to narrowly pointed apex (Fig. 29D); in ventral aspect moderately broad, lateral margins broadly curved, apex narrowly rounded (Fig. 29E). Lateral lobe broad basally, apical portion somewhat narrowed, evenly constricted to broadly rounded apex, with sparse setae apically (Fig. 29F).

*Female genitalia*. Gonocoxosternite broadly curved, apex narrowly rounded, medially deeply convex, anterior portion large and broad, anteriorly rounded (Fig. 41). gonocoxae with apical portion broad and short, apodemes elongate, slender and apically slightly expanded (Fig. 41). Bursa elongate and broad, membranous; spermathecal duct slender, moderately elongate; receptacle semispherical; spermatheca elongate and curved, not strongly differentiated, without spermathecal spine; fertilization duct short, slender and curved (Fig. 41).

*Sexual dimorphism*. Male pro- and mesotarsi I–III slightly more broadly expanded than female and ventrally with several large adhesive setae; female specimens examined are dorsally more alutaceous.

*Variation*. Specimens vary somewhat in intensity of coloration.

#### Distribution.

*Hydrodessus phyllisae* is known only from the Takutu Mountains of Guyana and Cerro de la Neblina in southern Amazonas, Venezuela (Fig. 47).

#### Habitat.

Specimens have been collected from blacklights and several forest habitats including muddy oxbow lakes, pools and leafpacks in whitewater streams, and stream margins.

#### Discussion.

Two female specimens from Paraguari, Paraguay (FSCA) resemble *Hydrodessus phyllisae* in many ways, but not such that they can be convincingly assigned to this species, and they are not included here as part of the concept of the species.

#### Specimens.

The holotype male in USNM was examined, it is labeled, “GUYANA: Mazaruni- Potaro District Takutu Mountains 6°15'N,59°5'W 16 December 1983/ EARTHWATCH Research Expedition: P. J. Spangler & W. E. Steiner Collectors/ At blacklight in forest clearing near streams / HOLOTYPE Hydrodessus phyllisae PJ Spangler [red label]/ BLNO 003805 [blue label with black line around margin].”

Other non-type specimens examined, 48 total. **Guyana**, Mazaruni-Potaro District, Takutu Mountains, 6.25°N, 59.083°W, 12 Dec 1983, R.A. Faitoute (2, KUNHM); same but 18 Dec 1983, berlese of leaf packs from rocky shaded stream, P.J. Spangler, W.E. Steiner, M. Levine (1, KUNHM); same but 17 Dec 1983, at blacklight in forest clearing near stream, P.J. Spangler, W.E. Steiner (2, USNM, including 1 paratype of *Hydrodessus phyllisae*). **Venezuela**, Amazonas, Cerro de la Neblina, 1km S basecamp, 0.833°N, 66.167°W, 19 Feb 1985, along small whitewater stream, pools of dead leaves and sticks, 140m, P.J. Spangler, P.M. Spangler, R. Faitoute, W. Steiner (24, USNM); same but Cerro de la Neblina, basecamp, 0.833°N, 66.167°W, 21 Feb 1985, rainforest clearing near Rio Baria, muddy oxbow pond, 140m, W.E. Steiner (13, USNM); same but Cerro de la Neblina, 1.5km S basecamp, 0.833°N, 66.167°W, 8 Feb 1985, small whitewater stream in rainforest, 250m, W.E. Steiner, R. Halling (1, USNM); same but Cerro de la Neblina, 1km S basecamp, 0.833°N, 66.167°W, 8 Feb 1985, netted along margins of Rio Baria, P.J. Spangler, P.M. Spangler, R. Faitoute, W. Steiner (1, USNM), same but Cerro de la Neblina, basecamp, 0.833°N, 66.167°W, 7 Feb 1985, at blacklight on bank of Rio Baria, 140m, W.E. Steiner (3, USNM).

### Hydrodessus rattanae

Taxon classificationAnimaliaColeopteraDytiscidae

Makhan, 1994: 118

Figs 30 , 51

Hydrodessus rattanae Makhan, 1994: 118; Biström 1988: 37; Nilsson 2001: 236.

#### Type locality.

Suriname, District Brokopondo, Brownsweg.

#### Diagnosis.

*Hydrodessus rattanae* is robust and broadly rounded with a distinctive dorsal pattern of maculae and fasciae (Fig. 30A). The head and pronotum are yellow (Fig. 30A). The elytra are brown with yellow lateral margins and large, well-defined maculae subbasally, apically and at about 2/3 length of elytra (Fig. 30A). Specimens do not have a lateral elytral carina, the epipleural carina extends nearly straight from the humeral angle (Fig. 30B). The prosternal process is elongate oval with the apex broadly pointed (Fig. 30C). The metaventrite carinae are distinctive and moderately divergent posteriorly (Fig. 30C). The male median lobe is basally narrowly triangular (Fig. 30D). The apical portion is long and nearly evenly curved with the apex narrow (Fig. 30D). In ventral aspect the median lobe is bilaterally symmetrical with the lateral margins narrowed to narrowly rounded apex (Fig. 30E). The lateral lobe is elongate-triangular with a long series of setae along the dorsal margin (Fig. 30F). This species is similar to *Hydrodessus laetus* in coloration, overall shape, lack of lateral elytral carinae, shape of the prosternal process and metasternum and other features. The male genitalia are diagnostic (Figs 30D–F). *Hydrodessus rattanae* is more robust, not as attenuate posteriorly and the color pattern is a little different (Fig. 30A). The metacoxal lines and regions mediad to the metacoxae are different, too (Fig. 30C). In *Hydrodessus rattane* the metacoxal lines are shorter, somewhat more divergent anteriorly and there are deep, longitudinal grooves along the medial margin of each metacoxal lines (Fig. 30C) that are missing in *Hydrodessus laetus* (Fig. 22C).

#### Description.

*Measurements*. TL = 2.6–2.7 mm, GW = 1.3–1.4 mm, PW = 1.1–1.2 mm, HW = 0.8–0.9 mm, EW = 0.5 mm, TL/GW = 1.9–2.0, HW/EW = 1.7–1.8. Body shape broad, posteriorly broadly, outline discontinous between pronotum and elytron (Fig. 30A), body somewhat depressed.

*Coloration* (Fig. 30A). Head and pronotum orange. Elytra fasciate, brown to brown-red with large irregular yellow regions transversely near anterior margin and medially, apex yellow, macula distinctly delimited, medial macula often separated into broad lateral marginal macula and smaller macula near suture (Fig. 30A). Antennae, palps and legs yellow. Ventral sclerites yellow, black along some sutures.

*Sculpture and structure*. Head broad, anteriorly broadly curved; surface shiny with fine punctures; eyes large. Pronotum with lateral margins broadly rounded, widest slightly anterior to middle (Fig. 30A); lateral bead fine; surface shiny with fine, indistinct microreticulation, covered with fine punctures. Elytra broad, lateral margins broadly curved, apically broadly; lateral carina absent, elytral epipleural carina extends directly posteriorly from humeral angle (Fig. 30B); surface with fine microreticulation throughout and covered with fine punctures. Prosternum medially tectiform; prosternal process broad, subrectangular, lateral margins subparallel, slightly constricted medially, apically broadly pointed, process medially deeply and broadly excavated (Fig. 30C). Metaventrite with anterior process short and broad, medially distinctly excavated, slightly constricted subapically, apically subtruncate; metasternal carina distinctive, straight and diverging posteriorly, posterior half indistinct, low and rounded, terminating at anterior ends of metacoxal lines (Fig. 30C); surfaces covered with fine punctation. Legs with surfaces shiny, weakly and indistinctly punctate; metatibia with posteroapical brush of setae distinctive; pro- and mesotibiae moderately broad; metatrochanter not strongly offset, elongate, apically narrowly rounded; metacoxa covered with fine punctation; metacoxal lines robust, well marked, narrowly separated, subparallael but slightly divergent anteriorly, longitudinally distinctly grooved mediad to metacoxal lines (Fig. 30C). Abdomen covered with fine punctation; apex of VI rounded.

*Male genitalia*. Median lobe bilaterally symmetrical, in lateral aspect robust, broadly and evenly curved, basal portion small and subtriangular, apical portion broad to rounded apex (Fig. 30D); in ventral aspect broad, lateral margins subparallel, apically slightly curved, and apex broadly rounded (Fig. 30E). Lateral lobe slender, elongate, without broad basal region, apex straight, evenly narrowed to rounded apex (Fig. 30F).

*Female genitalia*. Not examined.

*Sexual dimorphism*. Male pro- and mesotarsi I–III more broadly expanded than female and ventrally with several large adhesive setae.

*Variation*. Specimens exhibit some minor variation in the extend of the maculae on the elytron.

#### Distribution.

This species is known only from a couple localities in Suriname (Fig. 51).

#### Habitat.

A series of specimens was collected along the margins of a forest creek.

#### Discussion.

This species and *Hydrodessus laetus* are similar to each other and very different from many other species of *Hydrodessus* in the shape of the lateral margins of the elytron. The epipleural carina (between the epipleuron and dorsal surface of the elytron) extends posteriorly directly from the humeral angle. There is no other lateral carina. It remains to be seen whether these species are together monophyletic with the other members of *Hydrodessus*.

#### Specimens.

Holotype: ♂ in NZCS labeled, “Suriname District Brokopondo Brownsweg 7.8.1984 leg. D.Makhan/ Hydrodessus rattanae det. D. Makhan 1994/ Holotype [red label].” Other material examined, **Suriname**, Sipaliwini Dist, Tafelberg Summit nr Austustus Cr Camp, 3.933'N 56.183'W, 22 Aug 2013, forest creek margins, 600m, Short and Bloom (5, KUNHM, see accession numbers in Table 1).

### Hydrodessus siolii

Taxon classificationAnimaliaColeopteraDytiscidae

J. Balfour-Browne, 1953

Figs 31 , 43

Hydrodessus siolii J. Balfour-Browne, 1953: 56; Young 1967: 80; 1969: 2; 1970: 158; Spangler 1985: 88; Biström 1988: 37; Nilsson 2001: 236.

#### Type locality.

Brazil, Pará, Rio Cupari, Igarapé Ingatuba.

#### Diagnosis.

*Hydrodessus siolii* is a distinctive species with a pale head and pronotum and the elytra dark brown with the lateral margin yellow with distinctive, well defined yellow maculae (Fig. 31A). There is a prominent yellow macula on the elytral disc near the suture subbasally (Fig. 31A). There are a semiconnected pair of maculae at about 2/3 length of elytra (Fig. 31A). In some specimens the lateral macula is connected with the yellow lateral margin. Also, the elytral apex is yellow (Fig. 31A). The lateral elytral carina is absent (Fig. 31B). The prosternal process is elongate oval with the lateral margins slightly constricted medially (Fig. 31C). The prosternal process is elongate oval with the lateral margins somewhat constricted medially (Fig. 31C). The metaventrite carinae are indistinct and mainly marked by impunctate lines that are strongly divergent posteriorly (Fig. 31C). The male median lobe in lateral aspect is basally triangular (Fig. 31D). The apical portion is slender and evenly curved to a slender, narrowly rounded apex (Fig. 31D). In ventral aspect the median lobe is bilaterally symmetrical, apically convergent to elongate, slender, pointed apex (Fig. 31E). The lateral lobe is relatively slender, curved medially with the apical 1/3 relatively straight and slender to rounded apex (Fig. 31F). The species is perhaps most similar to *Hydrodessus fasciatus* in body shape and structure, but that species has a different color pattern and the male genitalia are distinctive in each species.

#### Description.

*Measurements*. TL = 2.7–3.1 mm, GW = 1.3–1.4 mm, PW = 1.1–1.2 mm, HW = 0.8–0.9 mm, EW = 0.4–0.5 mm, TL/GW = 2.1–2.2, HW/EW = 1.9. Body shape elongate, apically rounded, lateral outline discontinous between pronotum and elytron (Fig. 31A).

*Coloration* (Fig. 31A). Head and pronotum yellow. Elytron brown-yellow with diffuse yellow maculae anteromedially, medially and along margins anteriorly, mediolaterally, and at apex (Fig. 31A). Antennae, palps and legs yellow. Ventral surfaces yellow.

*Sculpture and structure*. Head moderately elongate; anterior clypeal marign broadly rounded; surface shiny, nearly impunctate; eyes large. Pronotum broadest slightly anterior of middle, lateral margins broadly curved (Fig. 31A); lateral bead fine; surface shiny, covered with moderately large, distinctive punctation. Elytra elongate, apically rounded (Fig. 31A); lateral carina absent, slight rounding of elytron near humeral angle (Fig. 31B); surface shiny, covered with moderately large, distinctive punctation. Prosternum medially rounded and setose; prosternal process moderately slender, elongate, lateral margins subparallel, widest subapically, apex rounded, longitudinally somewhat excavated (Fig. 31C). Metaventrite with anterior process slender, short, apically narrowly rounded; metasternal carinae distinct only anteriorly along margins of process, extending posteriorly in broadly divergent rounded margins, terminating near anterior ends of metacoxal lines (Fig. 31C); other surfaces covered with moderately large, distinct punctures. Legs with most surfaces shiny, impunctate; pro- and mesotibiae slender; metatibia with posteroapical brush of setae distinctive; metatrochater distinctly offset, apically rounded; metacoxa with surface shiny, covered with moderately large, distinctive punctation; metacoxal lines elongate, relatively closely approximated and subparallel, only slightly diverging anteriorly (Fig. 31C). Abdomen shiny, covered with distinctive punctures; ventrite VI apcially broadly rounded.

*Male genitalia*. Median lobe bilaterally symmetrical, in lateral aspect gently curved, curvature more pronounced basally, basal region broad, apical portion slender throughout length, apex slender and pointed (Fig. 31D); in ventral aspect slender, lateral margins evenly convergent to middle, then slightly constricted and apically slender to pointed apex (Fig. 31E). Lateral lobe moderately broad basally, elongate slender apically, apex rounded, with series of setae along dorsal margin (Fig. 31F).

*Female genitalia*. Not examined.

*Sexual dimorphism*. Male pro- and mesotarsi I–III slightly more broadly expanded than female and ventrally with several large adhesive setae. Females much more finely and densely punctate on all surfaces than males.

*Variation*. Specimens examined vary somewhat in the extend of maculation on the dorsal surface.

#### Distribution.

This species is known from central Brazil and southern Venezuela (Fig. 43).

#### Habitat.

This species was collected from “margem esquedra, entre detrito fibrosito” (Balfour-Browne 1953), or the “left bank, between fibrous detritous.” Specimens have also been collected along a sandy forest stream in marginal leaf pack.

#### Discussion.

The holotype (in BMNH) was not examined, but the male paratype (of one male and two female paratypes, Balfour-Browne 1953), which is now in the FSCA, was examined. Based on the description and the paratype examined, the identity of this species is clear.

#### Specimens.

Holotype not examined. Non-type specimens examined, 64 total. **Brazil**, Aldeia Aracu-Igarape, Gurupi-Umi, 50km E Caninde, 2°35'S 46°05W, 1–31 May 1963, B. Malkin (2, FSCA); Brazil, Pará, Boca Igarape Ingatuba, 3.723°S 55.404°W, 22 Oct 1948, H. Sioli (1, FSCA, paratype). **Venezuela**, Amazonas, Communidad Caño Gato, on Rio Sipapo, 4.981°N, 67.739°, 16 Jan 2009, along stream, 95m, Short, Miller, Camacho, Joly and García (61, KUNHM, MIZA, MSBA, USNM, museum numbers in Table 1).

### Hydrodessus spanus

Taxon classificationAnimaliaColeopteraDytiscidae

Spangler, 1985

Figs 32 , 44

Hydrodessus spanus Spangler, 1985: 83; Biström 1988: 37; Nilsson 2001: 236.

#### Type locality.

Guyana, Mazaruni-Potaro District, Takutu Mountains, 6°15'N 59°5'W.

#### Diagnosis.

This species has the elytron red with a moderately well-defined yellow macula at about 2/3 length of elytron (Fig. 32A). The pronotum is yellow and lighter in color than the elytron (Fig. 32A). The lateral carina on the elytron is low and rounded and mainly evident only near the humeral angle (Fig. 32B). The prosternal process is moderately narrow with the lateral carinae somewhat constricted medially and the apex rounded (Fig. 32C). The metaventrite carinae are only moderately distinct and clearly divergent posteriorly (Fig. 32C). The species is sexually dimorphic. Females have the anterolateral margin of the elytron flanged, unlike males. Females also have distinctive impressions on each side of abdominal ventrite VI. The male median lobe in lateral aspect is elongate triangular basally with the apical portion elongate, slender and curved with the apex distinctly sinuate and with a distinct angulation along the ventral margin subapically (Fig. 32D). In ventral aspect the apex is broadly rounded with distinct lateral teeth (Fig. 32E).

#### Description.

*Measurements*. TL = 2.7–2.8 mm, GW = 1.3 mm, PW = 1.1–1.3 mm, HW = 0.8–1.1 mm, EW = 0.5–0.8 mm, TL/GW = 2.1, HW/EW = 1.6–1.9. Body moderately robust, apically broadly pointed, lateral outline distinctly discontinuous between pronotum and elytron (Fig. 32A).

*Coloration* (Fig. 32A). Head brown, lighter posterolaterally and on clypeus. Pronotum yellow. Elytra brown to dark brown with submedial diffuse pale area and apex pale (Fig. 32A). Antennae and palps yellow. Legs yellow, dark red on ventral margins of femora. Venter dark red-black medially on prosternum, prosternal process, metasternum, metacoxae, abdominal ventrites, lighter laterally, becoming red to red-yellow on pronotal and elytral epipleura and laterally and apically on abdomen.

*Sculpture and structure*. Head broad, anterior clypeal margin broadly curved; surface shiny, covered with fine punctures; eyes moderately large. Pronotum subcordate, widest anterior of middle (Fig. 32A); lateral bead continuous and fine; surface with fine punctation, laterally with irregular rugosity. Elytra moderately elongate, apex broadly pointed (Fig. 32A); lateral carina low and rounded and only evident near humeral angle (Fig. 32B); surface covered with fine punctation. Prosternum medially tectiform and setose; prosternal process elongate, lateral margins distinctly constricted medially, shallowly impressed longitudinally, apex rounded (Fig. 32C). Metasternal process with apex truncated, medially flattened, subapically laterally constricted; metasternal carinae distinctive only along anterior process, extending posteriorly as line of impunctate surface (Fig. 32C); other surfaces covered with fine punctation. Legs covered with fine punctures on most surfaces; metatibia with distinctive brush of dense, elongate setae on postero-apical surface; pro- and mesotibiae moderately broad; metatrochanter distinctly offset, apically narrowly rounded; metacoxa evenly covered with fine punctures; metacoxal lines broadly separated, slightly curved, anteriorly somewhat divergent (Fig. 32C). Abdomen evenly covered with fine punctures; apex of VI slightly bisinuate, medially broadly pointed.

*Male genitalia*. Median lobe bilaterally symmetrical, in lateral aspect strongly and broadly curved, with basal region short and robust, apical portion strongly constricted, apically subsinuate, subapically slightly expanded and apex pointed (Fig. 32D); in ventral aspect robust and broad, lateral margins slightly curved and slightly divergent to broadly rounded, abruptly expanded apex (Fig. 32E). Lateral lobe basally broad, apical portion elongate triangular, apex broadly sub-truncate, apicodorsal margin with series of setae (Fig. 32F).

*Female genitalia*. Not examined

*Sexual dimorphism*. Male pro- and mesotarsi I–III more broadly expanded than female and ventrally with several large adhesive setae; female with elytron prominently expanded and lobate subapically, male evenly curved; male abdominal seternite VI evenly rounded across surface, apex with minute pointed lobe apically, female with prominent lateral depression on each side of VI.

*Variation*. Few specimens were examined, but they vary somewhat in the intensity of coloration.

#### Distribution.

*Hydrodessus spanus* are known from Guyana, Suriname and southeastern Venezuela (Fig. 44).

#### Habitat.

Specimens have been collected at a blacklight in a forest clearing near streams.

#### Specimens.

The holotype male in USNM was examined, labeled, “GUYANA: Mazaruni- Potaro District Takutu Mountains 6°15'N,59°5'W 17 December 1983/ EARTHWATCH Research Expedition: P. J. Spangler & W. E. Steiner Collectors/ At blacklight in forest clearing near streams/ HOLOTYPE Hydrodessus spanus PJ Spangler [red label]/ BLNO 003807 [blue label with black line around margin].”

Other non-type specimens examined, 3 total. **Suriname**, Sipaliwini District, Camp 1, Upper Palumeu, 2.175°N, 56.787°W, 19 Aug 2010, UV light, 228m, A.E.Z. Short (1, KUNHM, SEMC0914432). **Venezuela**, Bolivar, 85km SEE Dorado, 6.085°N, 61.399°W, 1 Nov 1982, E. Rubio, T. Borrego (1, KUNHM); Bolivar, San Ignacio, 9.567°N, 64.500°W, 8 Sep 1977, 1000m, B. Bechyne (1, MIZA, MIZA0001487).

### Hydrodessus surinamensis

Taxon classificationAnimaliaColeopteraDytiscidae

Young, 1970

Figs 33 , 44

Hydrodessus surinamensis Young, 1970: 153; Spangler 1985: 88; Biström 1988: 37; Nilsson 2001: 236.

#### Type locality.

Suriname, Carolina Creek, 10km S Zanderij.

#### Diagnosis.

*Hydrodessus surinamensis* has a characterstic coloration with the head and pronotum yellow and the elytra brown with distinct pale yellow maculae and lateral margins (Fig. 33A). The large subbasal macula exends to the lateral margin and, in a narrow subhumeral line, to the anterior margin (Fig. 33A). The apex of the elytron and a distinctive macula at about 3/4 elytral length are also yellow (Fig. 33A). The lateral elytral carina is short and distinctly only near humeral angle (Fig. 33B). The prosternal process is relatively narrow but has distinct laterally-directed lobes anteriorly (Fig. 33C). The metaventral process is narrowly rounded and the metaventrite carinae are indistinct, mainly represented by impunctate lines that diverge somewhat posteriorly (Fig. 33C). The male median lobe is simple and bilaterally symmetrical (Fig. 33E). In lateral aspect the median lobe is basally elongate triangular (Fig. 33D). The apical portion is shallowly curved to a pointed apex (Fig. 33D). In ventral aspect the median lobe is slender and narrowed medially to elongate, slender, pointed apex (Fig. 33E). The lateral lobe is slender and elongate-curved (Fig. 33F).

#### Description.

*Measurements*. TL = 2.3–2.5 mm, GW = 1.1 mm, PW = 0.9–1.0 mm, HW = 0.7–0.8 mm, EW = 0.4 mm, TL/GW = 2.2, HW/EW = 1.8. Body shape elongate, lateral margins strongly discontinous between pronotum and elytron (Fig. 33A).

*Coloration* (Fig. 33A). Head and pronotum yellow. Elytra brown to yellow-brown with three regions of yellow: 1) one large basal irregular macula extending medially to near suture, covering anterolateral region except small, round, brown spot, 2) one moderately large, subapical macula, and 3) apex. Antennae, palps, legs and other ventral surfaces yellow.

*Sculpture and structure*. Head broad, apically subtruncate in dorsal aspect, clypeal margin concave in anterodorsal aspect; surface very finely punctate; eyes large, conspicuous. Pronotum cordate, widest anterior of middle (Fig. 33A); lateral bead fine, obscured posteriorly; surface finely punctate. Elytra elongate, laterally evenly and broadly curved (Fig. 33A); lateral carina rounded, indistinct and limited to near humeral angle; elytral surface covered with fine punctures. Prosternum evenly rounded medially, not carinate; prosternal process elongate, broadest at base with laterally expanded lobes, posteriorly slender, lateral carinae proximate and covergent to narrowly rounded apex (Fig. 33C). Metaventrite with anterior process slender, carinae not strongly developed, posteriorly represented by low, rounded ridges ending distinctly mediad of anterior ends of metacoxal lines (Fig. 33C); surfaces covered with irregular punctures. Legs with surfaces covered with fine punctures; pro- and mesotibiae moderately broad; metatrochanter strongly offset from metafemur, apex broadly rounded; metatibia with posteroapical brush of setae; metacoxa covered with irregular punctures; metacoxal lines moderately broadly separated, straight and distinctly divergent anteriorly (Fig. 33C). Abdomen covered with irregular punctation; abdominal ventrite VI terminating in minute, medial, spinous lobe.

*Male genitalia*. Median lobe bilaterally symmetrical, in lateral aspect evenly but not strongly curved, with base small and subtriangular, apical portion elongate, slender, and evenly curved, apex slender and pointed (Fig. 33D); in ventral aspect slender and parallel sided in basal half, abruptly narrowed submedially and slender in apical half to narrowly rounded apex (Fig. 33E). Lateral lobe relatively narrow and evenly curved to slightly oblique apex (Fig. 33F).

*Female genitalia*. Not examined.

*Sexual dimorphism*. Male pro- and mesotarsi I–III more broadly expanded than female and ventrally with several large adhesive setae.

*Variation.* Specimens vary somewhat in extent of the maculae on the elytra surface.

#### Distribution.

This species is known from Suriname and Amazonas, Venezuela (Fig. 44).

#### Habitat.

This species has been collected from waterholes in a forest stream, tiny forest pools, large detrital pools, a large, sandy creek, and along a stream.

#### Discussion.

The holotype (in Rijksmuseum van Natuurlijke Historie, Leiden,Young 1970) was not examined, but nine paratypes (in FSCA) were, and the identity of this species is clear.

#### Specimens.

Specimens examined, 17 total. **Suriname**, Carolina Creek, 10km from Zanderij, 5.4°N, 55.183°W, 18 Nov 1962, waterhole in forest stream, B. Malkin (7, FSCA, paratypes); District XXV, Krakka-Phedra Road, 5.333°N, 55.086°W, 18 Nov 1962, tiny forest pool, B. Malkin (2, FSCA, paratypes); Sipaliwini District, Camp 1, Upper Palumeu, 2.477°N, 55.629°W, 14 Mar 2012, large sandy creek, 275m, A. Short (1, KUNHM, SEMC1088261); same except 10 Mar 2012, large detrital pools, 275m, A. Short (1, KUNHM, SEMC1089221). **Venezuela**, Amazonas, Communidad Caño Gato, on Rio Sipapo, 4.981°N, 67.739°, 16 Jan 2009, along stream, 95m, Short, Miller, Camacho, Joly and García (5, KUNHM, SM0843163, SM0843182, SM0843268, SM0843269, SM0843299).

### Hydrodessus tenuatus sp. n.

Taxon classificationAnimaliaColeopteraDytiscidae

http://zoobank.org/EB65B829-D15D-4650-9DD1-771D938969AC

Figs 7B , 34 , 45

#### Type locality.

Suriname, Sipaliwini Districct, Camp 1 on Kutari River, 2.175°N, 56.787°W.

#### Diagnosis.

This species is part of a group including *Hydrodessus maculatus*, *Hydrodessus latotibialis*, and *Hydrodessus phyllisae* that have the lateral elytral carina long (half or more the length of the elytron) (Fig. 34B), the prosternal process very broad (length/width < 2) (Fig. 34C), and the metaventral platform (the region between the metaventrite carinae) conspicuously constricted near the base of the metaventral process and fairly broadly divergent posteriorly (Fig. 34C). *Hydrodessus tenuatus* differs from *Hydrodessus maculatus* in having the elytra uniformly brown red (without maculae) and from *Hydrodessus latotibialis* and *Hydrodessus phyllisae* in having the pro- and mesotarsi relatively slender (not expanded with a subapical emargination). The male median lobe is basally triangular, curved at the base of the apical portion, and slender apically (Fig. 34D). The apex is slender and slightly curved to a pointed apex (Fig. 34D). In ventral aspect the laterla margins are broadly curved to moderatley rounded apex (Fig. 34E). The lateral lobe is broad basally, linear and evenly narrowed in apical half to rounded apex (Fig. 34F).

#### Description.

*Measurements*. TL = 2.8 mm, GW = 1.4 mm, PW = 1.2 mm, HW = 0.9 mm, EW = 0.5 mm, TL/GW = 2.0, HW/EW = 1.6. Body moderately robust, apically pointed, lateral outline moderately discontinous between pronotum and elytron (Fig. 34A).

*Coloration* (Fig. 34A). Head and pronotum orange. Elytra uniformly brown-red (Fig. 34A). Antennae and palps orange. Legs and ventral surfaces orange-red, darker near midline of metacoxa and on basal abdominal ventrite.

*Sculpture and structure*. Head moderately broad, anterior clypeal margins broadly rounded; surface shiny, microreticulate with numerous fine punctures; eyes large. Pronotum cordate, widest near anterior margin (Fig. 34A); lateral bead fine and distinct throughout length; surface shiny, covered with fine, indistinct punctures. Elytra elongate, apically pointed (Fig. 34A); lateral carina distinctive, elongate, extending about 3/5 length of elytron (Fig. 34B); surface shiny with punctures fine over entire surface, without lines on disc. Prosternum medially somewhat swollen, with fine medial carina; prosternal process moderately broad, widest at anterior angles, lateral margins slightly laterally compressed, deeply excavated medially, apically broadly truncate (Fig. 34C). Metaventrite with anterior process moderately broad, apically rounded, medially distinctly excavated; metasternal carinae distinctive across metasternum, anteriorly approximated, posteriorly distinctly divergent to posterior margin, terminating at anterior ends of metacoxal lines (Fig. 34C); metaventrite with lateral surface densely punctate. Legs shiny, most surfaces with very fine, indistinct punctures; metatibia with posteroapical brush of setae distinctive; pro- and mesotibiae moderately broad; metatrochanter somewhat offset, apically somewhat rounded; metacoxa densely punctate; metacoxal lines subparallel, anteriorly slightly divergent (Fig. 34C). Abdomen covered with fine punctures; VI apically narrowly rounded.

*Male genitalia*. Median lobe bilaterally symmetrical, in lateral aspect narrow basally, slender and evenly and broadly curved, medially slightly expanded, subapically slightly narrowed and curved to sharply pointed apex (Fig. 34D); in ventral aspect moderately broad, lateral margins broadly curved, apically broadly pointed (Fig. 34E). Lateral lobe broad basally, evenly narrowed apically to narrowly rounded apex, with small series of setae apically (Fig. 34F).

*Female genitalia*. Not examined.

*Sexual dimorphism*. Only the male holotype examined.

*Variation*. Only the male holotype examined.

#### Etymology.

This species is named *tenuatus*, Latin for “narrow,” for the relatively narrow mesotibia in specimens.

#### Distribution.

This species is known only from one locality in Suriname near the Kutari River (Fig. 45).

#### Habitat.

The one known specimen was collected at a UV light at night.

#### Specimens.

Only the holotype male was examined in NZCS labeled “SURINAME: Sipaliwini District 2°10.521'N, 56°47.244'W; 228 m Camp 1, on Kutari River leg. A.E.Z.Short; UV-light 19–24.vii.2010; SR10-0819-LT1 2010 CI-RAP Survey; SEMC0915670 KUNHM-ENT [barcode label]/ Hydrodessus sp. [handwritten] det. A.E.Z. Short 2011/ HOLOTYPE *Hydrodessus tenuatus* Miller, 2016 [red line with black line border].”

##### Species removed from *Hydrodessus*

### Amarodytes soekhnandanae

Taxon classificationAnimaliaColeopteraDytiscidae

(Makhan, 1994) comb. n.

Hydrodessus soekhnandanae Makhan, 1994: 117; Nilsson 2001: 236.

#### Type locality.

Suriname, Brokopondo District, Brownsweg.

#### Discussion.

This species was described by Makhan (1994) from a series from Suriname. Based on examination of the holotype, the species clearly does not belong to *Hydrodessus* since specimens have a distinctive pair of basal pronotal striae. As described by Makhan (1994), specimens have the pronotum with the “…base with two strongly incurvate plicae,” which are present in no other *Hydrodessus*. In fact, absence of these striae, or plicae, is one of the primary diagnostic features for the genus (Balfour-Browne 1953; Biström 1988; Young 1967; 1970), an important detail seemingly overlooked by Makhan (1994). Presence of these curved striae with simultaneous absence of basal elytral striae along with the coloration, lack of modified anterior clypeal margins, and other features, strongly suggest the species belongs to *Amarodytes* Régimbart, and it is transferred to that genus here (**comb. n.**). *Amarodytes* has not been revised, and there are numerous described species. It is possible the species is a junior synonym of another *Amarodytes* species.

#### Type specimen.

Holotype: ♂ in RMNH labeled, “Suriname District Brokopondo Brownsweg 7.8.1984 leg. D.Makhan/ Hydrodessus soekhnandanae det. D. Makhan 1994/ Holotype [red label].”

### List of species of *Hydrodessus*

*Hydrodessus amazonensis* Spangler, 1966: 380

*Hydrodessus angularis* Young, 1970: 155

*Hydrodessus biguttatus* (Guignot, 1957: 39)

*Hydrodessus fragrans* Spangler, 1985: 82, **syn. n.**

*Hydrodessus bimaculatus* **sp. n.**

*Hydrodessus brasiliensis* (Guignot, 1957:40)

*Hydrodessus brevis* **sp. n.**

*Hydrodessus concolorans* **sp. n.**

*Hydrodessus continuus* **sp. n.**

*Hydrodessus disjunctus* **sp. n.**

*Hydrodessus fasciatus* **sp. n.**

*Hydrodessus imparilis* **sp. n.**

*Hydrodessus jethoeae* Makhan, 1994: 119

*Hydrodessus keithi* **sp. n.**

*Hydrodessus kurti* **sp. n.**

*Hydrodessus kylei* **sp. n.**

*Hydrodessus laetus* **sp. n.**

*Hydrodessus latotibialis* **sp. n.**

*Hydrodessus maculatus* **sp. n.**

*Hydrodessus morsus* **sp. n.**

*Hydrodessus nanayensis* Spangler, 1966: 382

*Hydrodessus octospilus* (Guignot, 1957: 39)

*Hydrodessus robinae* Spangler, 1985: 85, **syn. n.**

*Hydrodessus palus* **sp. n.**

*Hydrodessus peloteretes* Spangler, 1985: 80

*Hydrodessus pereirai* (Guignot, 1957: 41)

*Hydrodessus phyllisae* Spangler, 1985: 86

*Hydrodessus rattanae* Makhan, 1994: 118

*Hydrodessus siolii* J. Balfour-Browne, 1953: 56

*Hydrodessus spanus* Spangler, 1985: 83

*Hydrodessus surinamensis* Young, 1970: 153

*Hydrodessus tenuatus* **sp. n.**

## Supplementary Material

XML Treatment for Hydrodessus

XML Treatment for Hydrodessus amazonensis

XML Treatment for Hydrodessus angularis

XML Treatment for Hydrodessus biguttatus

XML Treatment for Hydrodessus bimaculatus

XML Treatment for Hydrodessus brasiliensis

XML Treatment for Hydrodessus brevis

XML Treatment for Hydrodessus concolorans

XML Treatment for Hydrodessus continuus

XML Treatment for Hydrodessus disjunctus

XML Treatment for Hydrodessus fasciatus

XML Treatment for Hydrodessus imparilis

XML Treatment for Hydrodessus jethoeae

XML Treatment for Hydrodessus keithi

XML Treatment for Hydrodessus kurti

XML Treatment for Hydrodessus kylei

XML Treatment for Hydrodessus laetus

XML Treatment for Hydrodessus latotibialis

XML Treatment for Hydrodessus maculatus

XML Treatment for Hydrodessus morsus

XML Treatment for Hydrodessus nanayensis

XML Treatment for Hydrodessus octospilus

XML Treatment for Hydrodessus palus

XML Treatment for Hydrodessus peloteretes

XML Treatment for Hydrodessus pereirai

XML Treatment for Hydrodessus phyllisae

XML Treatment for Hydrodessus rattanae

XML Treatment for Hydrodessus siolii

XML Treatment for Hydrodessus spanus

XML Treatment for Hydrodessus surinamensis

XML Treatment for Hydrodessus tenuatus

XML Treatment for Amarodytes soekhnandanae
